# Genome-Wide Analysis Reveals Novel Genes Essential for Heme Homeostasis in *Caenorhabditis elegans*

**DOI:** 10.1371/journal.pgen.1001044

**Published:** 2010-07-29

**Authors:** Scott Severance, Abbhirami Rajagopal, Anita U. Rao, Gustavo C. Cerqueira, Makedonka Mitreva, Najib M. El-Sayed, Michael Krause, Iqbal Hamza

**Affiliations:** 1 Department of Animal and Avian Sciences, University of Maryland, College Park, Maryland, United States of America; 2 Department of Cell Biology and Molecular Genetics, University of Maryland, College Park, Maryland, United States of America; 3 Center for Bioinformatics and Computational Biology, University of Maryland, College Park, Maryland, United States of America; 4 The Genome Center, Department of Genetics, Washington University School of Medicine, St. Louis, Missouri, United States of America; 5 Laboratory of Molecular Biology, National Institute of Diabetes and Digestive and Kidney Diseases, National Institutes of Health, Bethesda, Maryland, United States of America; University of California San Diego, United States of America

## Abstract

Heme is a cofactor in proteins that function in almost all sub-cellular compartments and in many diverse biological processes. Heme is produced by a conserved biosynthetic pathway that is highly regulated to prevent the accumulation of heme—a cytotoxic, hydrophobic tetrapyrrole. *Caenorhabditis elegans* and related parasitic nematodes do not synthesize heme, but instead require environmental heme to grow and develop. Heme homeostasis in these auxotrophs is, therefore, regulated in accordance with available dietary heme. We have capitalized on this auxotrophy in *C. elegans* to study gene expression changes associated with precisely controlled dietary heme concentrations. RNA was isolated from cultures containing 4, 20, or 500 µM heme; derived cDNA probes were hybridized to Affymetrix *C. elegans* expression arrays. We identified 288 *heme-responsive gene*s (*hrg*s) that were differentially expressed under these conditions. Of these genes, 42% had putative homologs in humans, while genomes of medically relevant heme auxotrophs revealed homologs for 12% in both *Trypanosoma* and *Leishmania* and 24% in parasitic nematodes. Depletion of each of the 288 *hrg*s by RNA–mediated interference (RNAi) in a transgenic heme-sensor worm strain identified six genes that regulated heme homeostasis. In addition, seven membrane-spanning transporters involved in heme uptake were identified by RNAi knockdown studies using a toxic heme analog. Comparison of genes that were positive in both of the RNAi screens resulted in the identification of three genes in common that were vital for organismal heme homeostasis in *C. elegans*. Collectively, our results provide a catalog of genes that are essential for metazoan heme homeostasis and demonstrate the power of *C. elegans* as a genetic animal model to dissect the regulatory circuits which mediate heme trafficking in both vertebrate hosts and their parasites, which depend on environmental heme for survival.

## Author Summary

Heme is an iron-containing cofactor for proteins involved in many critical cellular processes. However, free heme is toxic to cells, suggesting that heme synthesis, acquisition, and transport is highly regulated. Efforts to understand heme trafficking in multicellular organisms have failed primarily due to the inability to separate the processes of endogenous heme synthesis from heme uptake and transport. *Caenorhabditis elegans* is unique among model organisms because it cannot synthesize heme but instead eats environmental heme to grow and develop normally. Thus, worms are an ideal genetic animal model to study heme homeostasis. This work identifies a novel list of 288 *heme-responsive genes* (*hrg*s) in *C. elegans* and a number of related genes in humans and medically relevant parasites. Knocking down the function of each of these *hrg*s reveals roles for several in heme uptake, transport, and detection within the organism. Our study provides insights into metazoan regulation of organismal heme homeostasis. The identification of parasite-specific *hrg* homologs may permit the selective design and screening of drugs that specifically target heme uptake pathways in parasites without affecting the host. Thus, this work has therapeutic implications for the treatment of human iron deficiency, one of the top ten mortality factors world-wide.

## Introduction

From a nutritional perspective, heme is a readily bioavailable source of iron for human consumption [1], [2]. From a cellular perspective, heme is an iron-containing porphyrin which serves as a prosthetic group in diverse biological processes ranging from gas-sensing to microRNA processing [3]. In most eukaryotes, heme is synthesized in the mitochondrial matrix by a defined biosynthetic pathway and subsequently exported as needed for heme-containing proteins that are found in the cytoplasm and membrane-bound organelles [3]. Given the hydrophobicity and cytotoxicity associated with free heme, it is likely that specific intracellular transport pathways exist to deliver heme for assimilation into hemoproteins found in various subcellular compartments [4].

Although the pathway and intermediates for heme biosynthesis and degradation have been well defined, the intracellular networks that mediate heme homeostasis in eukaryotes remain poorly understood [4]. Heme transport molecules in animals are likely to be divergent from bacterial and yeast proteins at the genetic level; bacterial and yeast heme-binding proteins have no obvious orthologs in mammals [5]–[8]. This is demonstrated by the identification of a heme exporter, the feline leukemia virus subgroup C cellular receptor (FLVCR), which does not show any obvious similarities to known bacterial heme transport proteins [9], [10]. Genetic ablation of FLVCR in mice resulted in severe macrocytic anemia with proerythroblast maturation arrest. That a viral receptor could be a potential heme exporter in developing erythroid cells underscores the divergence among heme transport proteins and emphasizes the importance of implementing unbiased genetic approaches to elucidate the heme homeostasis pathways in tractable model systems.

Progress in understanding heme homeostasis in most eukaryotic systems is hampered by the inability to separate heme biosynthesis from downstream intracellular transport pathways. To circumvent this issue, we established the genetically tractable nematode *Caenorhabditis elegans* as an animal model ideally suited in which to conduct heme studies. We have previously demonstrated that this roundworm does not synthesize heme but instead relies on environmental heme for survival [11]. Moreover, analyses of available genomes from related parasitic nematodes suggest that these helminths are also heme auxotrophs [11]. The *C. elegans* genome encodes a repertoire of hemoproteins that have vertebrate orthologs. It is likely that the pathways for heme trafficking and incorporation are conserved in *C. elegans*, parasitic worms, and vertebrates [4]. The validity of the *C. elegans* model system was recently underscored by the discovery of HRG-1 proteins that transport heme [12]. We identified *C. elegans hrg-1* and its paralog *hrg-4* from microarray experiments as genes that were highly upregulated by low heme [12]. Expression of these genes and their human homolog, *HRG-1*, in *Xenopus* oocytes resulted in strong heme-induced electrophysiological currents – an indication that the corresponding proteins were heme transporters. Additionally, depletion of *hrg-1* in worms led to aberrant heme homeostasis. Transient knockdown of *hrg-1* in zebrafish caused severe impairment in erythropoiesis along with brain and skeletal defects; these phenotypes were fully rescued by worm *hrg-1* [12]. Collectively, these studies further validated the advantage of *C. elegans* as a model *par excellence* to dissect the pathways responsible for heme transport and homeostasis in mammals. Moreover, *C. elegans* bridges the evolutionary divide to heme auxotrophic parasitic species and provides insight into helminthic-specific vulnerabilities in heme uptake and utilization that can be exploited for drug design [13], [14].

The current study specifically seeks to explain and draw conclusions from the genomic data that was generated from our microarray analysis. This expression array analysis using *C. elegans* wild-type worms grown in an axenic liquid medium at three different concentrations of heme was performed as a first step in the genome-wide identification of genes involved in heme homeostasis. Our results have identified several hundred *heme-responsive genes* (*hrg*s), some of which are evolutionarily conserved across metazoa while others are found only in nematodes. We anticipate that results from our genomic studies may be universally applicable and result in the discovery of heme homeostasis pathways in other metazoans.

## Results

### Strategy to profile genes that are transcriptionally regulated by heme in *C. elegans*

*C. elegans* lacks the highly conserved genes of heme biosynthesis but acquires heme from the environment for growth and development [11]. Worms cultured in axenic liquid mCeHR-2 medium in the presence of different amounts of heme revealed a characteristic growth curve [11]. The optimal concentration for worm growth and reproduction was found to be 20 µM heme, although animals grew and reproduced at concentrations ranging from ≥1.5 µM to <800 µM heme. Worms grown in the absence of exogenous heme arrested at the L4 larval stage, whereas concentrations of heme ≥800 µM caused the worms to arrest at the L2/L3 larval stages, possibly due to heme cytotoxicity. These results are consistent with metabolic labelling experiments in which the fluorescent heme analog, zinc mesoporphyrin IX (ZnMP), was used to demonstrate that the heme uptake system is regulated in *C. elegans* [12].

**Figure 1:**
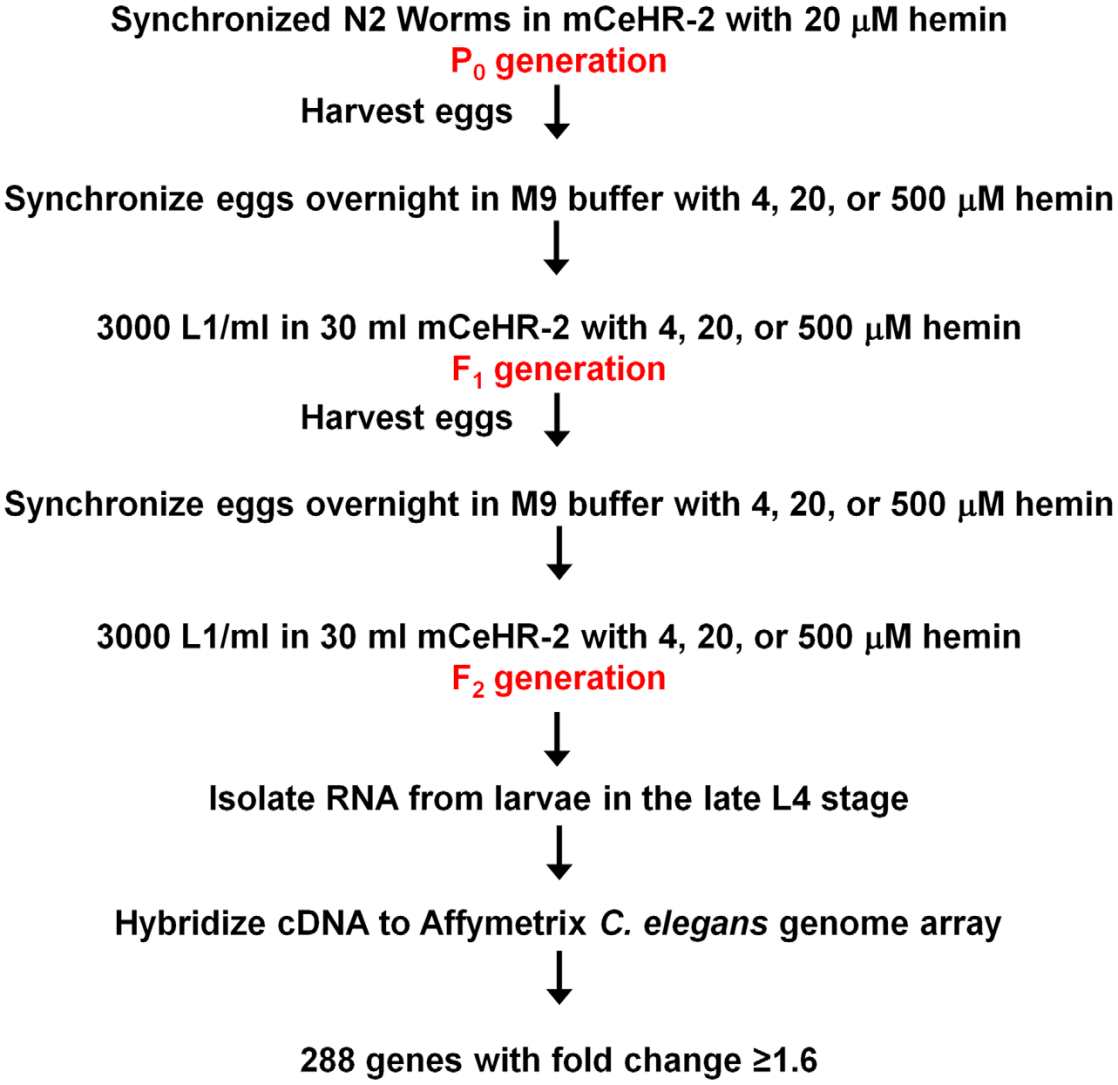
Profiling strategy for analysis of heme-responsive genes. Worms were grown in axenic liquid mCeHR-2 medium containing 4, 20, or 500 µM hemin chloride for two synchronized generations, and late L4 larvae were harvested to extract RNA for microarray analyses. cDNA was synthesized by reverse transcription and hybridized to Affymetrix *C. elegans* Genome Arrays containing 22,627 probe sets per chip. Affymetrix MAS 5.0 software and RMA were utilized to analyze the data. Data from worms grown at 4 and 500 µM heme were compared to control data from worms grown at 20 µM heme. The expression of 288 genes was either increased or decreased in response to heme by at least 1.6 fold.

To determine if there were transcriptionally regulated components of heme uptake, wild-type N2 worms were grown at 4, 20, or 500 µM heme in axenic liquid mCeHR-2 medium; 20 µM served as the reference sample. We chose 4 and 500 µM heme because these concentrations were on either side of the biphasic growth curve. More importantly, although worms grown at these heme concentrations exhibited a 16 h growth delay, they were morphologically indistinguishable from worms grown at 20 µM heme. In order to reduce variability due to carryover of maternal heme from the P_0_ hermaphrodites, worms were grown in their respective heme concentrations for two successive generations (Figure 1). Synchronized, late L4 larvae from the F_2_ generation were harvested for RNA isolation, and corresponding cDNA probes were generated and hybridized to Affymetrix *C. elegans* expression microarray chips. Three biological replicates were prepared for each heme concentration.

### Identification of *hrg*s in *C. elegans*

Statistical analyses of the microarray data were initially performed using the Affymetrix MAS 5.0 suite software (see Materials and Methods). Of the 22,627 probe sets, 835 probe sets revealed changes at either 4 or 500 µM heme compared to the control data from 20 µM heme. We identified 288 genes with a ≥1.6-fold change in expression. To improve and augment these analyses, we also subjected the microarray results to the Robust Multichip Average method (RMA from R package) with the goal of combining the results with those obtained by MAS 5.0. The RMA analysis (minimum change in expression ≥1.2 fold) identified an additional 82 *hrg*s. The MAS 5.0 and RMA analyses yielded a total of 370 candidate genes. Subsequently, duplicate genes were eliminated, the minimum cut-off value for RMA analysis was increased to ≥1.6 fold, and the average of the fold-change values was calculated for the replicates. This resulted in a list of candidate genes consisting of 266 genes identified using MAS 5.0 and 22 genes selected using the RMA method. The expression of these 288 genes, eight of which were previously identified as germline genes [15], revealed a ≥1.6-fold change at either 4 or 500 µM heme compared to the 20 µM controls. Consequently, all 288 genes were classified as *hrg*s (Table S1).

**Figure 2:**
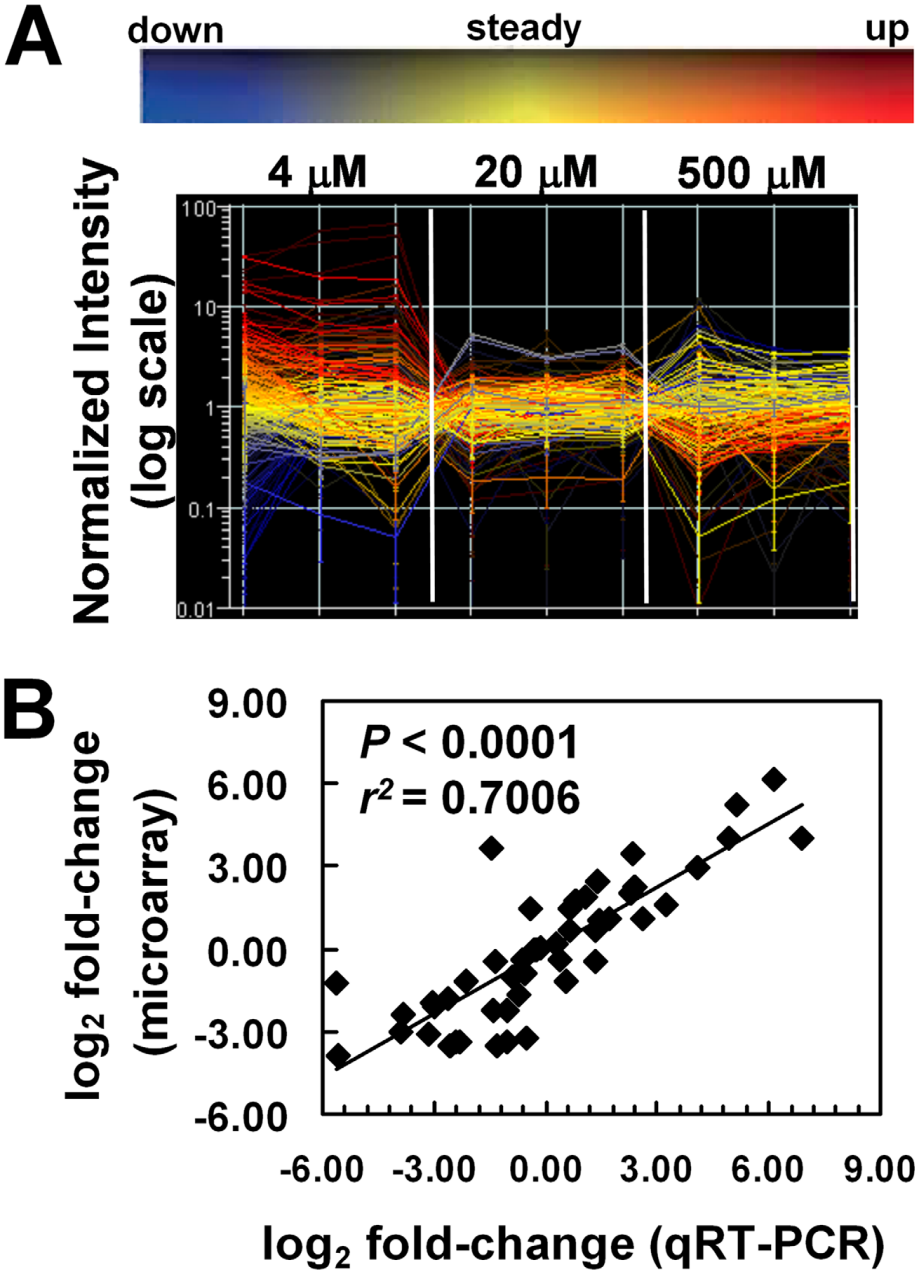
Confirmation of quality of microarray results. (A) Gene expression profile for the 288 *hrgs* identified from the microarrays. Changes in expression for the 288 *hrgs* are plotted for each biological replicate (thin vertical lines) of the three growth conditions with respect to heme concentration (4 µM, 20 µM, and 500 µM) as visualized with GeneSpring software (v7.2). Signal intensity values for genes in each biological replicate and each growth condition (as indicated) were normalized to the median value across the array after setting values <0.01 to 0.01 using GeneSpring software and the resulting values plotted on a log_10_ scale. Values for a single gene are connected by lines with the slope indicating any change in value across samples; line color coding reflects direction of change relative to the mean (as indicated by the color bar above the graph) and hue intensity reflecting statistical confidence in the value (confidence increases with increased brightness). (B) Validation of microarray results. Microarray data were verified by qRT-PCR of RNA from 24 genes of worms grown in mCeHR-2 medium supplemented with 4, 20, or 500 µM heme. RNA from 20 µM heme was used as the reference sample. Data were compared to internal GAPDH (*gpd-2*) control and the fold change was obtained using the 2 ^(-ΔΔCt)^ method. The significance was determined using GraphPad Instat (v. 3.06). The values for both the qRT-PCR analysis and the microarray experiment are provided in Table S4.

Normalized signal intensity values can be graphed to visualize the quality of microarray data generated by each replicate (Figure 2A). The value at which the colored lines cross each thin vertical line is the value of the normalized signal for that replicate. Accurate replicates should have nearly horizontal lines (all values approximately equal) within each condition that may then decrease or increase in the next condition if there is a change. In this experiment, analysis of each of the 288 *hrg*s revealed that individual biological replicates had nearly equal values with little variation within a particular heme concentration, indicating that changes in heme-dependent gene expression were uniform. A principal components analysis (PCA) for the *hrg*s showed that, with one exception, the quality of the microarray data was consistent across biological replicates for all three heme concentrations. The data obtained from one of the 4 µM heme replicates showed an inconsistent global gene expression pattern when compared to the other two replicates and was, therefore, excluded from further analysis (Figure S1).

**Figure 3:**
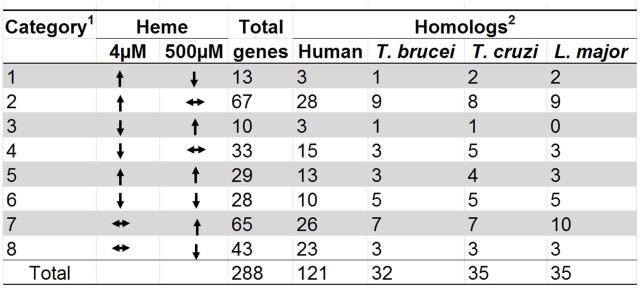
Heme-dependent changes in gene expression. ^1^The 288 *hrg*s were arranged into eight categories based on whether they were up-regulated, down-regulated, or unchanged at 4 µM or 500 µM heme when compared to control data from 20 µM heme. ^2^ *C. elegans* protein sequences for the 288 *hrg*s were used to perform reciprocal BLAST searches to identify putative homologs (E-value cut-off ≥10^−4^) in humans and protozoans.

The 288 *hrg*s were assigned to one of eight categories based on whether the gene expression was upregulated, downregulated, or unchanged in samples obtained from worms grown in 4 or 500 µM heme and compared to the 20 µM reference samples (Figure 3). Eighty genes were upregulated at 4 µM heme (Table S2). Seventy-five genes were upregulated at 500 µM heme (Table S3). Quantitative real-time PCR analysis (qRT-PCR) of three representative genes from each of the eight categories was performed to ensure that the changes observed in the microarray were reproducible. As determined by the significance (*P*<0.0001) and the Pearson's correlation coefficient, the qRT-PCR confirmed that the changes observed with the microarray results were consistent and, therefore, reliable (Figure 2B; Table S4).

### Comparative genome analyses of *hrg*s in vertebrates and parasites

Since identification of the *hrg*s common to both *C. elegans* and mammals might provide unique insights into the evolutionary conservation of heme homeostasis pathways in metazoans, we performed reciprocal BLAST searches to identify putative human orthologs of each of the 288 genes (Figure 3). Searches using protein sequences revealed that there were 121 putative human orthologs (minimum E-value  = 10^−4^) of *C. elegans hrg*s. The *hrg*s with human homologs were present among those upregulated in both extreme heme concentrations. Forty-four were upregulated at 4 µM heme and 42 were upregulated at 500 µM heme, while 28 were downregulated at 4 µM heme and 36 were downregulated at 500 µM heme (Table S1).

**Figure 4:**
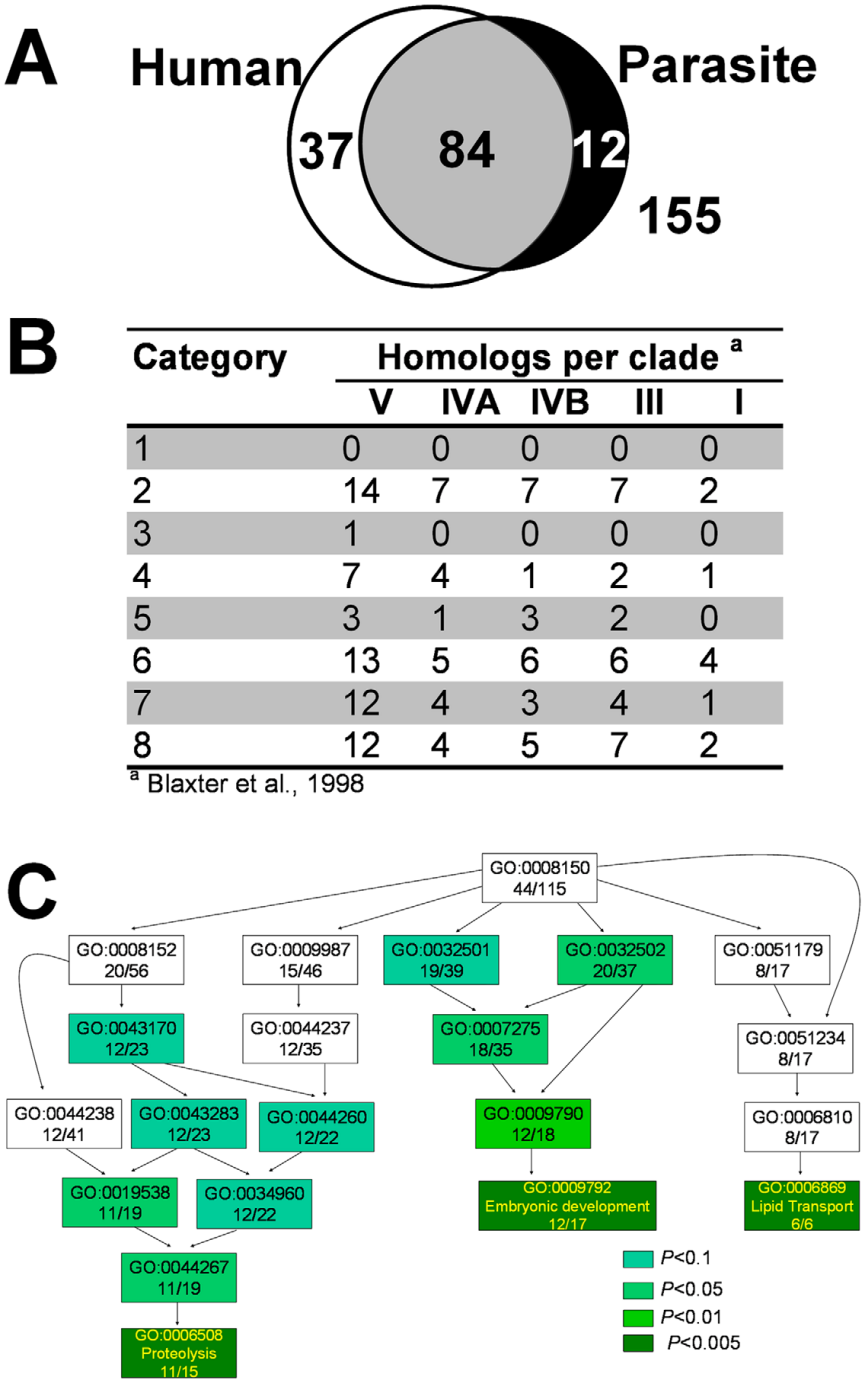
Comparative analysis of the heme-responsive genes. (A) Summary of overlap between *hrg*s across human and protozoan genomes. The sequences of proteins encoded by the 288 *hrgs* were obtained from Wormbase and used to search for homologs in the human genome and genomes of *Trypanosoma brucei, Tryoanosoma cruzi, and Leishmania major*. (B) Orthologs of *hrg*s in the genomes of parasitic nematodes. The 288 *C. elegans hrg* gene products were used to identify homologs in available parasitic nematode sequences. Based on 18S rRNA sequences, the phylum Nematoda is divided in five major clades; all five clades include parasites. Homologs were identified for 69 genes using amino acid sequences (at BLAST cut-off of 35 bits and 55% identity), and summarized on per clade level. A total of 440,012 peptides from 29 parasitic nematode species was used (clade I 29,203 peptides; clade III 145,044; clade IVA 13,636; clade IVB 92,514 and clade V 159,615 peptides). Nematode sequences used for this analysis are available on the parasitic nematodes website (http://www.nematode.net). (C) Gene ontology (GO) enrichment analysis of *hrgs* upregulated at 4 µM heme. Genes upregulated at 4 µM heme were analyzed using the Fisher's exact test and the topGO package from R. The most significant GO terms and their associated parent terms were used to construct a hierarchical graph such that the specificity of the terms increased from top to bottom. The text in each rectangle provides the GO ID and the ratio of the number of genes annotated with the GO term in the tested subset to that in the total gene set. The shade of green of each rectangle corresponds to the significance of the GO result. The complete table of *P-*values can be found in Table S6. Full GO terms are provided solely for genes with *P*<0.005.

We have previously demonstrated by biochemical enzyme assays and genomic analyses that several of the parasitic nematodes with sequenced genomes lack the genes for heme synthesis enzymes and, therefore, likely rely on environmental heme to sustain growth and development [11]. Similarly, the genomes of *Trypanosoma* and *Leishmania* appear to lack most of the genes for heme synthesis [16], [17]. This suggests that these protozoa may also acquire heme from their parasitized host. Figure 3 identifies the *hrg* homologs in *Trypanosoma brucei*, *Trypanosoma cruzi*, and *Leishmania major*. Of the 288 *hrg*s, only 12 genes were exclusive to these heme auxotrophs. Thirty-seven genes had homologs only in humans, and 84 genes were found in both human and parasitic genomes (Figure 4A). These results indicate that heme-regulated genes in *C. elegans* may have commonality with humans that are heme prototrophs and protozoan parasites which rely on environmental heme.

A small percentage of the 288 *hrg*s had homologs in parasitic nematodes (Figure 4B). To date, draft genomes of several parasitic nematodes have become available [18]–[21], in addition to the partial genomes available for over 30 parasitic species. For a summary of available genomes, see [22]. Using all available sequence data divided into taxonomically distinct clades [23], we identified homologs for 62 of the 288 *hrgs* in the clade V nematodes (*C. elegans* belongs to clade V) and homologs to only 10 genes in the clade I nematodes (where the basal nematode, the zoonotic parasite *Trichinella spiralis*, resides). While the number of identified putative orthologs was much higher for the crown lineages than in the basal nematodes that reside at the root of the nematode evolutionary tree, two of the eight categories (categories 1 and 3) had no homologs in any of the parasitic species. Categories 1 and 3 are represented by 13 and 10 sequences in *C. elegans*, respectively.

### *hrg*s are enriched in regulators for development- and transport-related processes

Gene ontology (GO) analysis [24] indicated that the *hrg*s identified from our microarray study were involved in processes as varied as embryonic development, electron transport, lipid metabolism, and iron-sulfur cluster assembly. Of the 288 genes in the study, 115 were annotated with a biological process (Table S5). Using the Fisher's exact test, a hierarchical graph was constructed with the most significant GO terms and their associated parent terms [25]. Highly significant GO terms (*P*<0.005) associated with the subset of genes that were upregulated at 4 µM heme were ‘embryonic development’, ‘lipid transport’, and ‘proteolysis’ (Figure 4C; Table S6); ‘responses to stress’ and environmental stimuli' were associated with genes that were downregulated at 4 µM heme (Figure S2 and Tables S7, S8, S9).

The Kyoto Encyclopedia of Genes and Genomes (KEGG) is also frequently used to analyze complex microarray data and make functional predictions [26]. Only 10 *hrg*s (∼3%) have been mapped to KEGG pathways (Table S10). These hits included genes for transporters and also for metabolism of sugars, an amino acid, and fatty acids. A majority of *hrg*s that we identified were uncharacterized with no assigned biological pathway.

**Figure 5:**
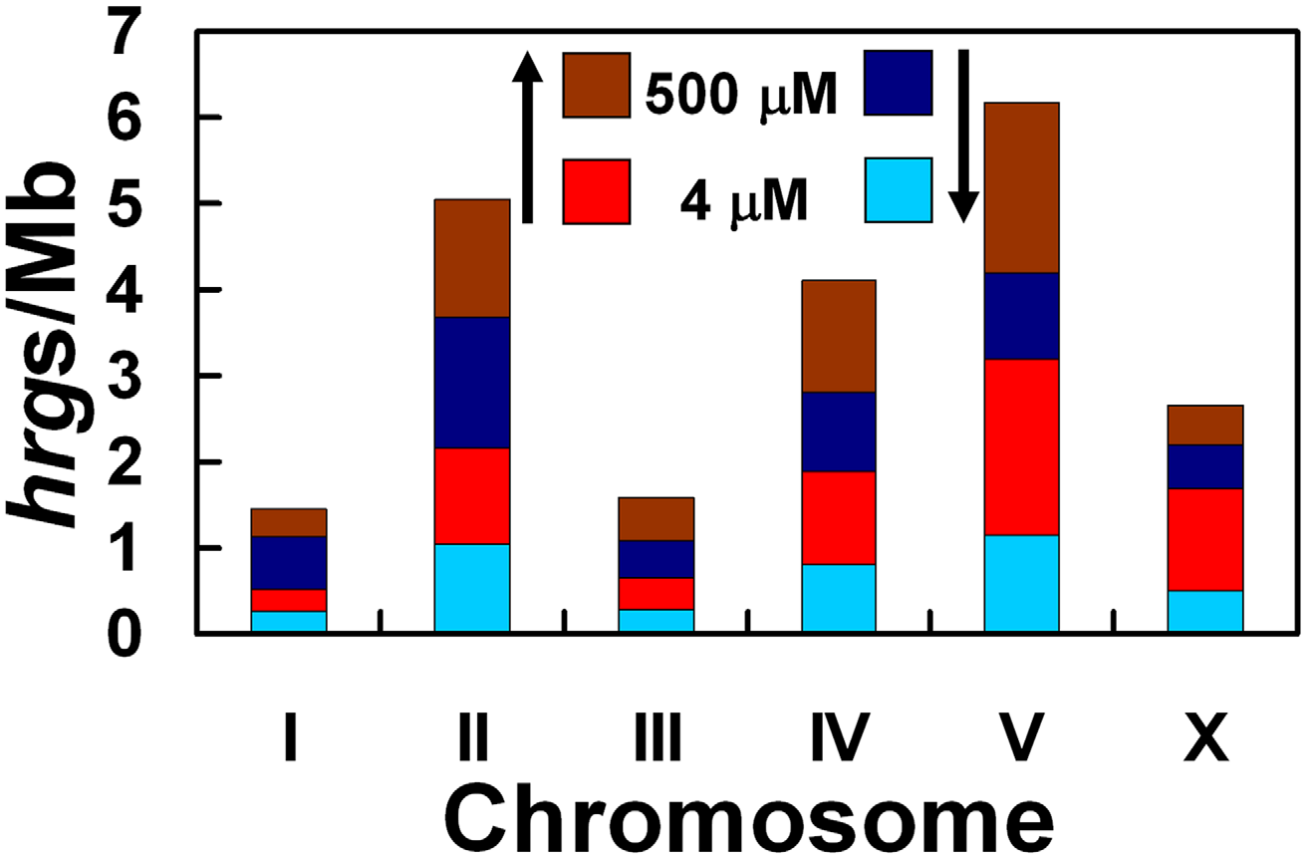
Depiction of the number of *hrgs* found on each chromosome relative to the number of megabases in that chromosome. The chromosome with the highest number of *hrg*s is chromosome V. Red and brown regions indicate that the expression of a gene was increased at 4 and 500 µM heme, respectively. Light blue and dark blue bars represent a decrease in gene expression at 4 and 500 µM heme, respectively.

**Figure 6:**
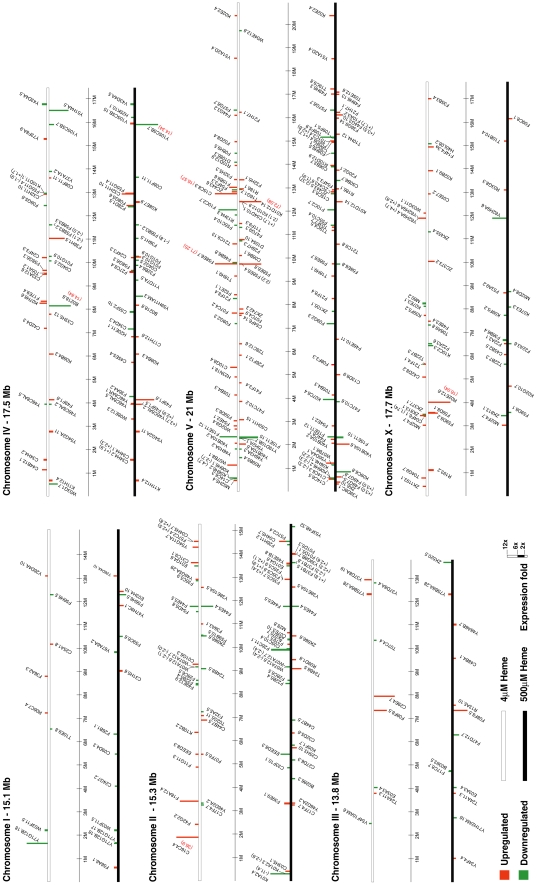
Chromosomal location and fold-change of each heme-responsive gene. *hrg*s were arranged on each of the six chromosomes, which are depicted as white or black bars. The transcript levels of genes placed on white bars were altered at 4 µM heme, while the expression of genes on black bars was significantly altered at 500 µM heme. Vertical bars are drawn to scale and represent genes that were up-regulated (red) or down-regulated (green).

Genome sequencing has demonstrated that chromosomes I, II, III, IV, and X in *C. elegans* each contain roughly equivalent numbers of genes (13–17%), whereas chromosome V has the most genes (25%) [27]. Furthermore, co-regulated or functionally related genes, especially those essential for interactions with the environment, tend to reside in local clusters on the chromosome [27]. We found that Chr I and Chr III each contained just 6% of the *hrg*s, but 35% of all *hrg*s were found on Chr V (Figure 5). Additionally, of the 129 *hrg*s on Chr V, 43 genes were upregulated at 4 µM heme while 41 genes were upregulated at 500 µM heme (Figure 6). Our analysis suggests that the genomic distribution of *hrg*s was non-random, reveals gene clustering, and indicates a common biological response to an environmental stimulus such as heme.

If the genomic distribution of *hrg*s is purposeful, we reasoned that perhaps the response of the promoters of the *hrg*s is directed by a *cis*-acting element within a cluster or elements that are common to all *hrg*s in a specific category. First we analyzed Categories 1 and 2 for overrepresented transcription factor binding sites using all sequences against a control set of random promoter sequences but failed to identify common *cis* elements. We reiterated our search to encompass the presumptive promoters (≥2 kb upstream) of all 288 *hrg*s using TRANSFAC [28]. Once again, no common elements were identified.

### Functional validation reveals novel *hrg*s that are essential for heme homeostasis in *C. elegans*

A number of genome-wide RNA-mediated interference (RNAi) experiments have been performed in *C. elegans*, and the data from all these experiments are available on Wormbase (http://www.wormbase.org/). Forty-six *hrg*s (16%) had a reported RNAi phenotype (Table S11). RNAi knockdown of these genes most often resulted in developmental defects such as sterility and embryonic lethality. These phenotypes were expected because heme is essential for growth and reproduction of *C. elegans*. The relatively small fraction of genes yielding a reported RNAi phenotype probably reflects redundancy of function among some of the *hrg*s and the limited phenotypic assays performed to date.

We have previously reported that transgenic worms expressing the *hrg-1::gfp* transcriptional fusion (strain IQ6011) specifically respond to heme in the growth medium. Thus, strain IQ6011 can be used as a whole animal heme sensor to interrogate changes in organismal heme homeostasis [12]. To identify the function of the *hrg*s in heme homeostasis, we established a functional RNAi screen using IQ6011 (see Materials and Methods for details). First, we generated a sequence-confirmed *hrg* mini-library in the *E. coli* feeding strain HT115(*DE3*) that expressed double-stranded RNA (dsRNA) against each of the 288 *hrg*s. Second, we established a sensitive GFP-based assay that conditionally screened for genetic modulators of heme homeostasis simultaneously in the presence of low (5 µM) or high (25 µM) heme. Third, we verified the positive candidate genes with a secondary screen to eliminate false positives using a *vha-6::gfp* transgenic worm that does not respond to heme and served as a negative control. Fourth, we confirmed the authenticity of each candidate gene by simultaneously measuring the GFP fluorescence intensity in IQ6011 and *vha-6::gfp* with a COPAS Biosort instrument that sorts each worm by its time of flight (axial length of object) and extinction (optical density of object).

**Figure 7:**
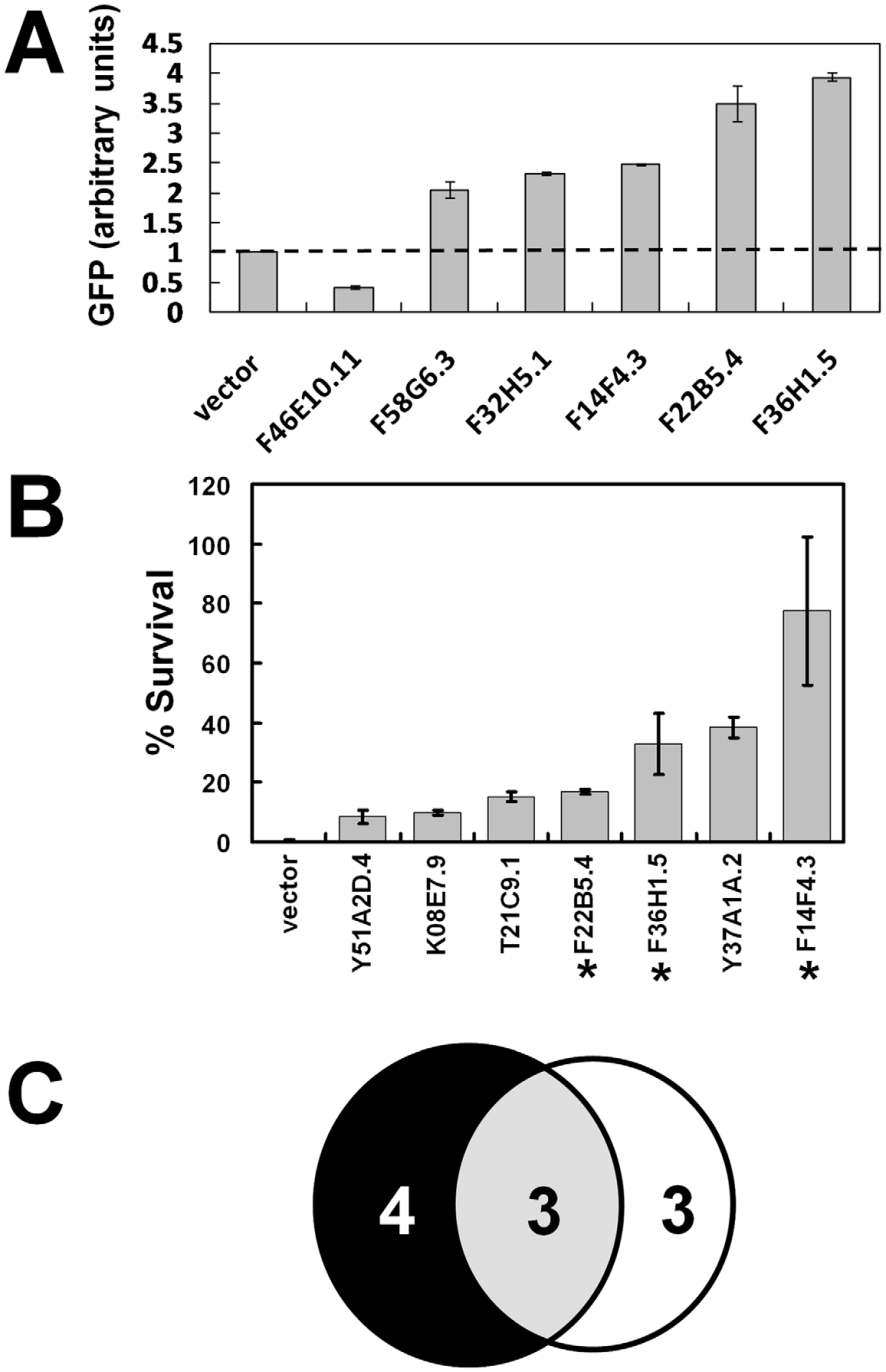
Functional validation of heme-responsive genes. (A) RNAi depletion in a transgenic heme-sensor strain. GFP quantification by COPAS BioSort in strain IQ6011 fed HT115(*DE3*) bacteria grown in 5 µM heme and induced to synthesize dsRNA. RNAi of 288 *hrg*s identified six genes which caused a 2-fold increase or 2-fold decrease in GFP levels. Each data point represents the mean ± SEM. Y-axis represents average GFP values of all the gravid worms (≥30 worms in each well) in duplicate wells normalized to the value of the GFP in worms that had fed on bacteria transformed with the empty vector. (B) RNAi depletion in the presence of a toxic heme analog. Forty-one candidate genes encoding proteins with TMD were screened by feeding RNAi to strain IQ6011 and assessing survival of progeny in the presence of the toxic heme analog GaPP. Both the total number of eggs and the number of viable larvae were counted after 5 days of exposure to 1.5 µM GaPP as described in Materials and Methods. Each data point represents the mean ± SEM of two separate experiments and is depicted as percentage of survival compared to control plates with no GaPP. Knockdown of 7 genes (shown) resulted in animal survival in the presence of GaPP. Asterisk indicates genes that also altered GFP levels in strain IQ6011 worms (Figure 7A). (C) Summary of overlap between genes identified in (A,B). Six of the 288 *hrg*s were identified as interfering with heme homeostasis, as evidenced by their altered GFP levels at 5 µM. Seven of the 41 *hrg*s that encode for proteins with putative TMD protect against GaPP toxicity, as evidenced by an increase in progeny survival. When knocked down by RNAi, only three *hrg*s both alter the ability to sense heme and protect against GaPP toxicity.

Synchronized IQ6011 worms were grown in mCeHR-2 medium supplemented with 10 µM heme to repress GFP and subsequently transferred to NGM agar plates for exposure to dsRNA produced by *E. coli* grown either in the presence of 5 µM or 25 µM heme on NGM agar plates. These experiments were performed in duplicate, and GFP levels and patterns in worms fed bacteria expressing each of the 288 *hrg*s were analyzed by eye. RNAi depletion of the 288 *hrg*s resulted in the identification of 32 genes that specifically upregulated or downregulated GFP expression in the IQ6011 heme-sensor strain but not in the *vha-6::gfp* control strain. These 32 genes were selected for further analysis by the COPAS BioSort. We identified six *hrg*s which caused either a two-fold increase or a two-fold decrease in GFP expression, (Figure 7A). A significant upregulation of GFP was observed at 5 µM when five *hrg*s that encoded either putative membrane-spanning proteins (F36H1.5/HRG-4, F14F4.3/MRP-5, F58G6.3/CTR-1, and F22B5.4/unnamed protein) or a putative lysosomal cysteine protease (F32H5.1/cathepsin-L) were depleted. In contrast, GFP was downregulated only when F46E10.11, which encodes an uncharacterized protein proposed to bind metals through cysteine residues, was depleted.

To identify potential heme transporters, we identified *hrg*s which encoded for proteins with transmembrane domains (TMD). TMHMM analysis predicted that 41 of the 288 *hrg*s encoded for proteins with at least one putative TMD (Table S12). Among these 41 genes were those encoding aquaglyceroporin-related proteins (*aqp-1* and *aqp-8* with six and four TMD, respectively) that transport small molecules such as glycerol, urea, and water; *cyp-33C9* (one TMD) which belongs to the cytochrome P450 family of heme binding proteins; heme permeases (*hrg-1* and *hrg-4* with 4 TMD); and ABC transporters (*mrp-5* and *pgp-1* with ≥12 TMD).

To narrow the list of candidate heme transporters, we used RNAi to deplete the 41 *hrg*s which encoded TMD proteins and exposed the worms to gallium protoporphyrin IX (GaPP), a toxic heme analog that causes severe defects in worm growth and development [11]. We reasoned that knockdown of a putative heme transporter in the presence of GaPP would result in a concomitant increase in worm survival due to a reduced ability to transport toxic GaPP [12]. We identified seven *hrg*s which, when depleted by RNAi, revealed greater survival of the F_1_ progeny at 1.5 µM GaPP, a concentration that is lethal to wild-type worms (Figure 7B). These seven genes included F36H1.5/*hrg-4*, F14F4.3/*mrp-5*, K08E7.9/*pgp-1*, Y51A2D.4/*hmit1.1*, Y37A1A.2, T21C9.1, and F22B5.4. Three genes – *hrg-4*, *mrp-5*, and F22B5.4 – were positive in both of the RNAi screens (Figure 7C). Taken together, our genomic studies identified a small subset of genes that are not only regulated by heme at the mRNA level but are also essential for heme transport and homeostasis at the functional level.

**Figure 8:**
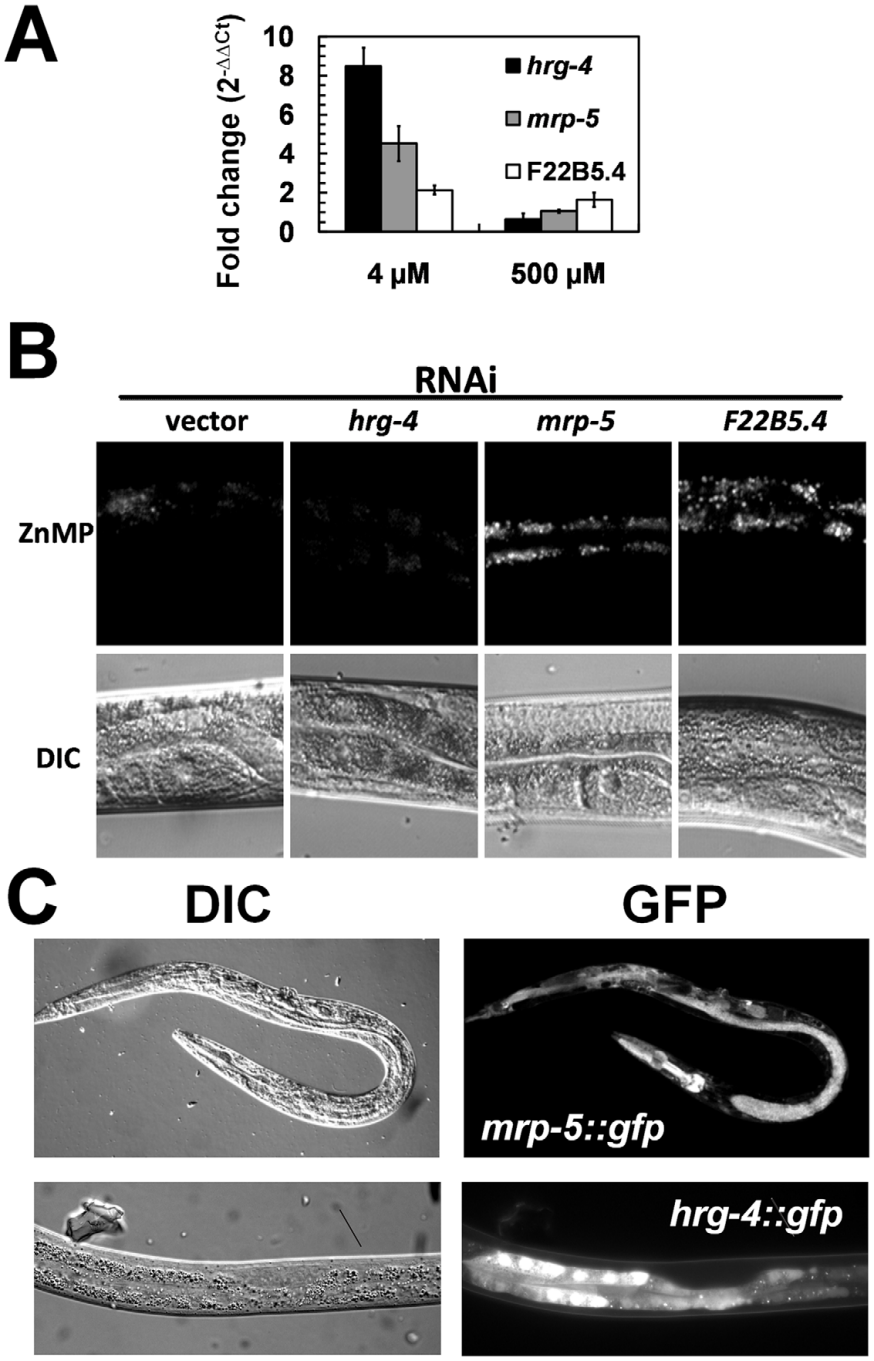
Characterization of the three candidate genes identified from the functional RNAi screens. (A) Heme-dependent expression. qRT-PCR was performed in triplicate for *hrg-4*, *mrp-5*, and F22B5.4 at the indicated heme concentrations, and the fold change (mean ± SEM) is reported for each gene at each concentration of heme. (B) Accumulation of ZnMP in worms depleted of candidate genes by RNAi. Synchronized wild-type strain worms in the L1 stage were transferred from mCeHR-2 medium containing 2 µM heme to plates seeded with a lawn of bacteria producing dsRNA against vector, *hrg-4*, *mrp-5*, or F22B5.4 and allowed to develop for ≈72 h to the late L4 stage. At this point, worms were incubated in mCeHR-2 medium containing 5 µM ZnMP overnight. Images of a region of worm intestine were captured using a Leica DMIRE2 microscope equipped with a Rhodamine filter and a CCD camera. (C) Expression of *hrg-4* and *mrp-5* GFP transcriptional reporters. Transgenic worms expressing GFP under the control of 3 kb of the *hrg-4* (bottom) or *mrp-5* (top) putative promoters. Transgenic worms were grown in mCeHR-2 medium containing 4 µM heme for one generation. Representative images of worms were obtained with a Leica DMIRE2 microscope using a CCD camera.

To better understand the role of *hrg-4*, *mrp-5*, and F22B5.4 in heme homeostasis, we determined their mRNA expression in response to heme and their ability to transport heme as a function of ZnMP accumulation [12]. qRT-PCR results indicated that all three genes were upregulated by heme but the magnitude of change in mRNA expression at 4 µM heme was significantly greater for *hrg-4* than *mrp-5* or F22B5.4 (8.5-fold versus 4.5- and 2-fold) (Figure 8A). Heme uptake assays with ZnMP revealed that *hrg-4* RNAi resulted in abrogation of ZnMP accumulation in the worm intestine compared to wild-type control worms (Figure 8B), a result consistent with our previous studies [12]. By contrast, ZnMP accumulation was dramatically increased by the knockdown of both *mrp-5* and F22B5.4. Although HRG-4 has been implicated in intestinal heme transport in *C. elegans*, no function has been attributed to either F22B5.4 or MRP-5 in WormBase. Membrane topology algorithms predicted that, unlike F22B5.4, which is predicted to contain a single TMD, HRG-4 and MRP-5 contain four and twelve TMD respectively, a characteristic feature of membrane transporters.

To correlate the intestinal ZnMP uptake studies with membrane transport, we examined the gene expression pattern of *hrg-4* and *mrp-5*. We generated transgenic worms that expressed *hrg-4::gfp* and *mrp-5::gfp* transcriptional fusions. *hrg-4::gfp* was expressed specifically in the intestinal cells of larvae and adults (Figure 8C), and was regulated by exogenous heme (not shown). Unlike *hrg-4::gfp*, we found that *mrp-5::gfp* was expressed in almost all worm tissues examined. Altogether, these studies identify HRG-4 and MRP-5 as membrane transporters that are essential for intestinal heme homeostasis in *C. elegans*.

## Discussion

A major impediment to the identification of heme uptake and transport pathways has been the inability to disassociate the tightly regulated process of heme synthesis from the downstream pathways for heme transport [3]. *C. elegans* is unique among the model organisms because it does not synthesize heme but, instead, relies solely on exogenous heme for normal growth and development [11]. Thus, worms allow the study of heme homeostatic mechanisms in response to fluctuations in environmental heme within the context of an intact, live animal.

Our study of the genome-wide transcriptional changes associated with heme availability represents, to the best of our knowledge, the first study of nutrient-gene interactions in *C. elegans* exploiting axenic liquid growth medium. The mCeHR-2 medium permits fine control of organismal heme levels as a function of heme in the growth medium, allowing us to identify 288 *heme-responsive genes* (*hrgs*). Some of the genes we identified were predictable, because they encode either known heme-binding proteins or permeases for transport of other small molecules. Other genes made sense in retrospect, such as glutathione transferases (GST). A recent proteomic analysis of *C. elegans* identified GST-19 as a highly abundant protein that was proposed to sequester heme when intracellular heme is in excess [29]. GSTs have also been shown to bind heme in helminths such as hookworms and Barber pole worms [30]–[32]. Our genomic analysis indicates that *gst-22* and *gst-16* were upregulated at 500 µM heme. Whether these GST proteins also bind heme remains to be determined.

GO and KEGG pathway analyses reveal that *hrg*s represent the full spectrum of biological processes. Interestingly, only a few *hrg*s are enzymes or proteins that are known to bind heme. We speculate that the transcriptional regulation by heme primarily targets the cellular pathways involved in heme homeostasis, including uptake and sequestration, rather than the genes which encode target hemoproteins. The vast majority of *hrg*s have no known function and, therefore, do not have any biological processes or pathways attributed to them. Furthermore, phenotypes from RNAi studies involving the 288 *hrg*s reported growth and developmental defects, plausibly because disruption of heme homeostasis will affect hemoprotein function in diverse biological pathways ranging from miRNA processing (DGCR8) to gas sensing (soluble guanylyl cyclases) to circadian clock control (Rev-erbα) [33]–[35].

The 288 *hrg*s we identified also provide the first insight into metazoan heme regulation. The fact that >40% of *hrg*s have human homologs suggests that our study may provide genetic insights into mammalian heme regulation. This is underscored by the presence of human homologs for genes that were positive in our functional RNAi screen. Indeed, recent studies using *C. elegans* as a model system have led to the identification of HRG-1 as the first *bona fide* metazoan heme importer that is conserved in vertebrates [12].

Analysis of the presumptive promoters of all 288 *hrg*s in eight categories identified no common *cis* elements [28]. A more detailed analysis of the 67 genes in Category 2, to which *hrg-1* and *hrg-4* belong, found no overrepresented transcription factor binding sites using all sequences against a control set of random promoter sequences. These *in silico* results corroborate our experimental studies (Chen, Sinclair, and Hamza, unpublished results) and further support the concept that regulation of organismal heme homeostasis is complex, multi-tiered, and effected by diverse cellular modulators.

Studies have demonstrated that the infectivity of hookworms, which feed on the blood of the host, is significantly lower in severely anemic hamsters fed a low-iron diet [13]. Furthermore, filarial nematodes, such as the causative agent of elephantiasis, harbor *Wolbachia* – an intracellular bacterial symbiont that contains the intact heme biosynthesis pathway [14], [36]. Thus, nematodes may have adapted to heme auxotrophy by evolving pathways to acquire heme either from the host (extracellular) or from the symbiotic relationship with the bacteria (intracellular). This auxotrophy can be exploited to develop drugs that block parasite-specific heme uptake or utilization. Indeed, genome database searches of heme auxotroph parasites led us to identify 12 *hrg* homologs in protozoans and 62 *hrg*s in clade V nematodes. This finding is significant because these genes may encode proteins involved in heme uptake and sequestration from the parasitized host. Further studies aimed at elucidating the role of these *hrg*s in heme metabolism may validate them as novel anti-parasitic drug targets.

We found that seven of the 41 *hrg*s that encode for proteins which contain putative TMDs showed different levels of resistance against GaPP toxicity. Among these were a heme permease (HRG-4), ABC transporters (PGP-1 and MRP-5), and Major Facilitator Superfamily transporters (HMIT-1.1 and Y37A1A.2). The remaining 247 *hrg*s encoded proteins without any predicted TMDs. These proteins may encode soluble effectors for heme transport such as chaperones or sequestering proteins. In support of this concept, cellular iron is stored in ferritin, a cytosolic multi-subunit protein; cytoplasmic copper is delivered to membrane bound P-type ATPases in the secretory pathway by the copper chaperone Atox1 [37], [38]. We propose that a similar network for trafficking intracellular heme and maintaining homeostasis is likely to exist in *C. elegans* and most metazoa [3].

Interestingly, HRG-4, MRP-5, and F22B5.4 were the only positive candidates identified in both the heme-sensor and GaPP functional RNAi screens. RNAi studies have implicated HRG-4 as a heme transporter in the *C. elegans* intestine [12], while the function of MRP-5 and the protein encoded by F22B5.4 are currently unknown. We speculate that MRP-5, a member of a family of membrane effluxers [39], may export heme from the intestinal cells to extra-intestinal cells. These results are consistent, in part, with the ubiquitous expression of *mrp-5::gfp* in worm tissues, and with the RNAi studies which show that *mrp-5* depletion results in accumulation of ZnMP in the worm intestine and resistance to GaPP toxicity. Unlike HRG-4 and MRP-5 which are transporters with multiple TMD, F22B5.4 encodes a predicted Type II membrane protein with a single TMD. Although our results clearly implicate a role for F22B5.4 as an essential component of heme homeostasis in *C. elegans*, it is unclear how this protein may function in heme homeostasis. Excitingly, microarray and RNAi studies identified F22B5.4 as a gene that is highly upregulated by the hypoxia-inducible factor (HIF) transcription complex, a master regulator of hypoxia response [40]–[42]. HIF is regulated by degradation through hydroxylation of proline residues, a process which requires the presence of oxygen, 2-oxoglutarate, and iron [43]. Given the dependence of *C. elegans* on heme for oxygen binding and sensing [44]–[46] and as a nutritional source of iron [11], it is conceivable that F22B5.4 may play an important role in coordinating heme transport and availability with oxygen metabolism.

In the current study we have identified a novel catalog of genes that are responsive to heme in *C. elegans*. Although it is unclear mechanistically how worms respond to heme at the mRNA level, a thorough study to identify the *cis* regulatory elements and the corresponding *trans* acting factors will significantly accelerate our understanding of how *C. elegans* adapts to environmental and nutritional changes. Using the facile and genetically tractable *C. elegans* model system, the RNAi screen with the *hrg* mini-library can be easily adapted for whole genome screens to identify regulatory pathways which govern how metazoans sense and respond to heme at an organismal level.

## Materials and Methods

### Biological materials, strains, and worm culture

*C. elegans* wild-type N2 strain worms were grown either in an axenic liquid mCeHR-2 medium [47] or on NGM agar plates spotted with *E. coli* OP50 or HT115(*DE3*) strains [48]. Synchronized, L1 larvae were obtained by bleaching P_0_ gravid worms grown in mCeHR-2 medium supplemented with hemin chloride [11]. Hemin chloride and gallium protoporphyrin IX were purchased from Frontier Scientific, Inc (Logan, UT). Plasmids for cloning and injecting into worms were part of the Fire Vector Kit (Addgene, Cambridge, MA). Primers designed to PCR amplify worm open reading frames were based on Wormbase predictions and ordered from IDT (Coralville, IA). The PCR products were TA cloned into the L4440 plasmid.

### Total RNA isolation

Equal numbers of F_1_ larvae in the L1 stage were inoculated in mCeHR-2 medium with 4, 20, or 500 µM hemin chloride and grown with gentle shaking at 20°C. Synchronized, F_2_ larvae in the L1 stage were obtained by hatching the eggs obtained from F_1_ gravid adults in M9 buffer containing 4, 20, or 500 µM hemin. Equal numbers of F_2_ larvae in the L1 stage were inoculated in mCeHR-2 medium supplemented with 4, 20, or 500 µM hemin. The F_2_ worms were allowed to develop to the late L4 stage, harvested, flash frozen in liquid nitrogen, and stored at −80°C. Frozen worm pellets were ground into a fine powder, and total RNA was extracted using Trizol (Invitrogen, Carlsbad, CA). RNA thus obtained was subjected to RNase-free DNase treatment for 1 h at 37°C and purified using the RNeasy kit (Qiagen, Germantown, MD). Total RNA from three biological replicates was used to make cDNA, which was then hybridized to *C. elegans* Whole Genome Arrays (Affymetrix, Santa Clara, CA).

### cDNA synthesis and quantitative real-time PCR

First strand cDNA was synthesized using 2 µg of total RNA using a Superscript II First Strand cDNA synthesis kit (Invitrogen). For quantitative real-time PCR (qRT-PCR), primers spanning at least one intron were designed using Primer Express (Applied Biosystems) and Beacon designer 4 (Premier Biosoft) programs. PCR was performed using the iCycler iQ Real-time PCR Detection System (BioRad) with 0.12 U/µl Taq DNA polymerase, 40 nM fluorescein (Invitrogen), and SYBR Green I Nucleic Acid Gel Stain (Invitrogen) diluted 1:10. The PCR amplification was run for 40 cycles. The PCR products were between 150 and 200 bp in length. Quality of the PCR products was determined by dissociation curve analysis and gel electrophoresis. Each experiment was performed in triplicate. Average C_T_ values were used for 2^−ΔΔCt^ calculations of relative fold changes in gene expression [49].

### Microarray data analysis

Expression data were normalized and analyzed using MAS 5.0 suite software (Affymetrix). Data from worms grown in mCeHR-2 medium with 4 and 500 µM hemin were compared to data from worms grown in medium containing 20 µM hemin (baseline samples). Microarray data were verified with the Robust Multichip Average Method (RMA, R package). Quantile normalization and background corrections were performed using perfect match probe intensities. Using an initial cut-off of ≥1.2-fold change in mRNA expression for RMA and a ≥1.6-fold change for MAS 5.0 resulted in the identification of 370 genes. Increasing the stringency to ≥1.6-fold change for both RMA and MAS 5.0 reduced the number of genes identified as heme responsive to 288 genes. To identify putative human orthologs, worm protein sequences were used to query human genome databases at NCBI by reciprocal BLAST analysis with an E-value cut-off ≥10^−4^. Sequences for each of these 288 genes were obtained from WormBase and further analyzed for topology (TMHMM 2.0, SOSUI), motifs (ELM, BLOCKS, Pfam), and pathway classification (GO and KEGG).

### Generation of the *hrg* mini-library

The Ahringer and Vidal feeding libraries were replicated to individual 96-well plates [50], [51]. Thirty-four clones in the initial list of 370 *hrg*s were absent from both libraries. To complete the *hrg* mini-library, we PCR amplified the missing genes from N2 worm genomic DNA and cloned the PCR fragments by TA cloning into the RNAi feeding vector pL4440. Only 19 of the 34 RNAi clones were in the final list of 288 *hrg*s. DNA for all 288 *hrg*s was sequenced to confirm authenticity.

### Analysis of RNAi on GFP expression in the IQ6011 and *vha-6::gfp* strains

NGM agar plates containing IPTG, carbenicillin, and tetracycline were seeded with HT115(*DE3*) bacteria expressing double-stranded RNA (dsRNA) against each clone in the *hrg* mini-library. Duplicate bacterial cultures of each clone had been grown for 5.5 h in LB containing carbenicillin and tetracycline and 5 µM or 25 µM heme. Plates were seeded with a lawn of bacteria and dsRNA induction occurred for ≈20 h at room temperature. Subsequently, forty L1 larvae from gravid IQ6011 worms which had been grown in liquid media supplemented with 10 µM heme were added to each well of the 12-well plates. Each 12-well plate had 10 wells seeded with experimental clones and one well seeded with each of the control clones – vector and *hrg-4*. The plates were incubated at 15°C overnight and then incubated at 20°C for three additional days. The GFP levels in gravid adults were observed visually using a Leica Microsystems MZ16FA stereoscope. The intensity and pattern of GFP in gravid worms feeding on bacteria producing dsRNA against each *hrg* was compared to the intensity and pattern of GFP in same-stage worms feeding on bacteria transformed with the empty vector. Worms that displayed altered GFP in both replicates were designated as potential modulators. Potential modulators were screened in a strain that produced GFP under the control of a promoter that was not responsive to heme (*vha-6::gfp*). Any clone that altered GFP levels in the *vha-6::gfp* strain worms was removed from the list of modulators, since the change in GFP was not in response to heme.

### GFP quantification in IQ6011 strain

A COPAS BioSort worm sorter (Union Biometrica, Holliston, MA) was used to measure GFP levels in live worms. Plates, bacteria, and worms were prepared and treated as described in the previous section. After 84 h on RNAi plates, P_0_ gravid and F_1_ L1-stage worms were washed from each well with 600 µL of M9 buffer containing 0.01% Tween-20, transferred to a 1.5-mL microcentrifuge tube, and allowed to settle for 5 min. The supernatant was removed and discarded. Each worm pellet was transferred to an individual well of a 96-well plate. Duplicate samples were transferred to successive wells in the 96-well plate and were separated from other samples by an empty well, which served to flush the flow cell where worms are analyzed and prevent contamination of subsequent samples. The contents of each well were washed, aspirated, and analyzed by a COPAS BioSort worm sorter. The GFP gain was set to 2, and the GFP PMT setting was 400. Using highly synchronized worms in the gravid stage, we had previously defined the gate settings in order to ensure that the data obtained from P_0_ gravid animals would be easily and quickly separated from the data obtained from worms in other developmental stages. Text file data was imported into Microsoft Excel and sorted based on the gating parameters recorded in the “Status Select” column. The worm sorter records a fluorescence profile of each worm in the form of a curve, which reflects the intensity of GFP from the mouth to the tail. The “Green” column recorded the GFP value of the area under the curve, reduced by a factor of 40,000. The background levels of GFP were subtracted from all values used to generate Figure 7A. The background level of GFP was equal to the GFP levels in IQ6011 worms feeding on HT115(*DE3*) bacteria transformed with the *gfp* RNAi vector. The COPAS BioSort detects very low levels of GFP in these worms. The mean of all values for each sample was determined, and the average of each duplicate was calculated. This mean was normalized to the average value for the GFP obtained from the vector-only sample, and reported in arbitrary units ± SEM for each clone analyzed.

### GaPP toxicity assays

Synchronized, F_1_ wild-type worms in the L1 larval stage were obtained from P_0_ worms grown in mCeHR-2 containing 1.5 µM hemin. Equal numbers of these F_1_ worms were placed on NGM agar plates containing 2 mM IPTG, 50 µg/mL carbenicillin, 12 µg/mL IPTG and plated with a lawn of HT115(*DE3*) RNAi feeding bacteria harboring the respective L4440 plasmid that had been grown in LB broth with carbenicillin and tetracycline [12]. Worms were fed on the RNAi bacteria for ≈60 h and allowed to develop to the late L4 stage. At this point, worms were transferred to fresh RNAi plates containing 1.5 µM GaPP. Worms developed to the gravid stage and laid eggs. After 24 h of egg-laying, the P_0_ worms (all in the gravid stage) were discarded in order to prevent additional eggs from being laid. On day 5, both the total number of surviving larvae and the number of unhatched eggs were counted. *P* values for statistical significance were calculated by using a one-way ANOVA with Student–Newman–Keuls multiple comparisons test by using GraphPad InStat v. 3.06 (GraphPad, San Diego, CA).

### ZnMP uptake assays

Equal numbers of synchronized N2 L1 larvae obtained from P_0_ worms grown in mCeHR-2 plus 2 µM hemin were exposed to the RNAi bacteria on NGM plates containing 2 mM IPTG for 72 h. This was followed by exposure to 5 µM ZnMP plus 1.5 µM hemin chloride for 16 h in mCeHR-2 medium. ZnMP fluorescence intensity was measured as described previously [12].

### Generation of GFP reporter constructs

GFP reporter fusion constructs were created using the Gateway cloning system (Invitrogen, Frederick, MD). The promoter of interest, *gfp* gene, and the 3′ untranslated region of the *unc-54* gene were cloned by recombination into the entry vectors pDONR P4-P1R, pDONR 221, and pDONR P2R-P3, respectively, using the Gateway BP Clonase kit. Sequence verified entry clones were then recombined into a destination vector pDEST R4-R3 using the Gateway LR Clonase II plus enzyme kit to produce the final recombinant plasmid.

### Production of transgenic worm strains

For microparticle bombardment, ≈5×10^6^ *unc-119* (*ed3*) gravid worms were co-bombarded with 10 µg of Gateway reporter construct and 5 µg of *unc-119* rescue plasmid (pDM016B) using the PDS-1000 particle delivery system (Bio-Rad, Hercules, CA). Worms were washed from bombardment plates and transferred to plates seeded with a lawn of *E. coli* strain JM109. After two-weeks at 25°C, multiple wild-type F_2_ worms were screened for gene integration either by PCR or transgene expression. Individual transgenic lines were isolated and transferred to axenic liquid mCeHR-2 medium supplemented with antibiotics. After two weeks of serial passages, worms were bleached and maintained as transgenic strains in axenic liquid mCeHR-2 medium.

### Gene Expression Omnibus information

The microarray data was submitted to GEO on Aug 6, 2007. The GEO accession number is GSE8696 and available at

http://www.ncbi.nlm.nih.gov/geo/query/acc.cgi?acc=GSE8696.

## Supporting Information

Figure S1 Heat map for the heme microarrays.
A compilation of heat maps generated following normalization of the data (see Figure 2 legend) using GeneSpring (v7.2) for each category of the 288 *hrgs* with data from all nine chips represented. The up and down arrows indicate upregulation or downregulation in 4 or 500 µM heme when compared to 20 µM heme. Yellow represents no change in signal intensity, blue indicates a decrease, and red indicates an increase in signal intensity. The data from the first replicate sample from 4 µM heme, indicated with an asterisk at the top of the column, were inconsistent with the data from the other two biological replicates as determined by both principal components analysis and K-means clustering of the data.
(2.43 MB TIF)

Figure S2 Gene ontology (GO) enrichment analysis of heme-responsive genes.
(A) *hrg*s downregulated at 4 µM heme. (B) *hrg*s upregulated at 500 µM heme. (C) *hrg*s downregulated at 500 µM heme. Of the 288 *hrg*s identified in the study, 115 were annotated with a biological process. Genes were analyzed using the Fisher's exact test and the topGO package from R. The most significant GO terms and their associated parent terms were used to construct a hierarchical graph such that the specificity of the terms increased as we moved from top to bottom. The text in each rectangle provides the GO ID and the ratio of the number of genes annotated with the GO term in the tested subset to that in the total gene set. The shade of green of each rectangle corresponds to the significance of the GO result. Full GO terms are provided solely for genes with *P*<0.005. The complete table of *P-*values and a full description of the GO term associated with each gene can be found in Tables S7, S8, and S9.
(0.08 MB PDF)

Table S1 The 288 heme-responsive genes identified by the microarray.
Data was collected using the Affymetrix *C. elegans* whole genome array and analyzed by both Affymetrix MAS 5.0 software and RMA. Each entry in the table represents a gene whose expression changed at least 1.6 fold at one or both of the experimental heme concentrations. The table has six columns for each *hrg*. The “Description” column lists the unique Gene ID assigned by Wormbase to every gene in the *C. elegans* genome. The “Gene name” column provides the name of a gene, when one has been assigned. The first “4 µM” column gives the value of the change of expression of each gene, and the second “4 µM” column indicates whether the gene expression was increased (up) or decreased (down). If the column is blank, then the change was less than 1.6-fold. The pattern for the “500 µM” columns is the same as for the “4 µM” columns.
(0.10 MB PDF)

Table S2 Heme-responsive genes whose expression is upregulated greater than 1.6 fold in worms grown at 4 µM heme.
The gene ID (description), gene name, and amount of change at 4 µM compared to the control (20 µM) are provided for each gene whose expression increased at 4 µM.
(0.08 MB PDF)

Table S3 Heme-responsive genes whose expression is upregulated greater than 1.6 fold in worms grown at 500 µM heme.
The gene ID (description), gene name, and amount of change at 500 µM compared to the control (20 µM) are provided for each gene whose expression increased at 500 µM.
(0.73 MB TIF)

Table S4 Heme-responsive genes used to corroborate the microarray results. Three genes were selected from each of the eight categories designed to show whether the expression of a gene increased, decreased, or did not change at a given heme concentration compared to the 20 µM control.
(1.15 MB TIF)

Table S5 Heme-responsive genes with known Gene Ontology terms.
Of the 288 *hrgs* whose expression changed significantly in response to heme, the results of a gene ontology analysis were used to assign a known biological process and molecular function to 63 genes.
(1.02 MB TIF)

Table S6 Gene Ontology analysis of heme-responsive genes upregulated at 4 µM heme.
Each GO ID is assigned a unique function or association. Both are listed here, even if the GO ID was not used in the GO analysis figure. Green shading indicates that term was included in the corresponding GO enrichment figures.
(0.06 MB PDF)

Table S7 Gene Ontology analysis of heme-responsive genes downregulated at 4 µM heme.
Each GO ID is assigned a unique function or association. Both are listed here, even if the GO ID was not used in the GO analysis figure. Green shading indicates that term was included in the corresponding GO enrichment figures.
(0.06 MB PDF)

Table S8 Gene Ontology analysis of heme-responsive genes upregulated at 500 µM heme.
Each GO ID is assigned a unique function or association. Both are listed here, even if the GO ID was not used in the GO analysis figure. Green shading indicates that term was included in the corresponding GO enrichment figures.
(0.09 MB PDF)

Table S9 Gene Ontology analysis of heme-responsive genes downregulated at 500 µM heme.
Each GO ID is assigned a unique function or association. Both are listed here, even if the GO ID was not used in the GO analysis figure. Green shading indicates that term was included in the corresponding GO enrichment figures.
(0.09 MB PDF)

Table S10 Heme-responsive genes assigned to a biological pathway by KEGG analysis.
The algorithms available on the Kyoto Encyclopedia of Genes and Genomes website were used to make functional predictions for each of the 288 *hrg*s identified in the microarray. Ten *hrg*s were mapped to KEGG pathways.
(0.59 MB TIF)

Table S11 Previously reported RNAi phenotypes of heme-responsive genes.
Phenotypes observed when *hrg*s were knocked down in experiments performed by other laboratories and compiled on Wormbase.
(0.85 MB TIF)

Table S12 Heme-responsive genes with predicted TMDs.
Worm protein sequences obtained from Wormbase were analyzed using TMHMM 2.0 and SOSUI to identify 41 proteins with putative hydrophobic membrane-spanning domains (TMDs). The 41 genes with putative TMDs have been arranged according to the number of TMDs. The change in levels of gene expression at 4 and 500 µM heme is indicated. Negative fold change implies down regulation.
(0.74 MB TIF)
