# Supplementary figures and images for: Genome-Wide Analysis Reveals Novel Genes Essential for Heme Homeostasis in Caenorhabditis elegans
Source: PLoS Genet. 2010 Jul 29;6(7):e1001044. doi: 10.1371/journal.pgen.1001044 (PMC2912396; doi:10.1371/journal.pgen.1001044)

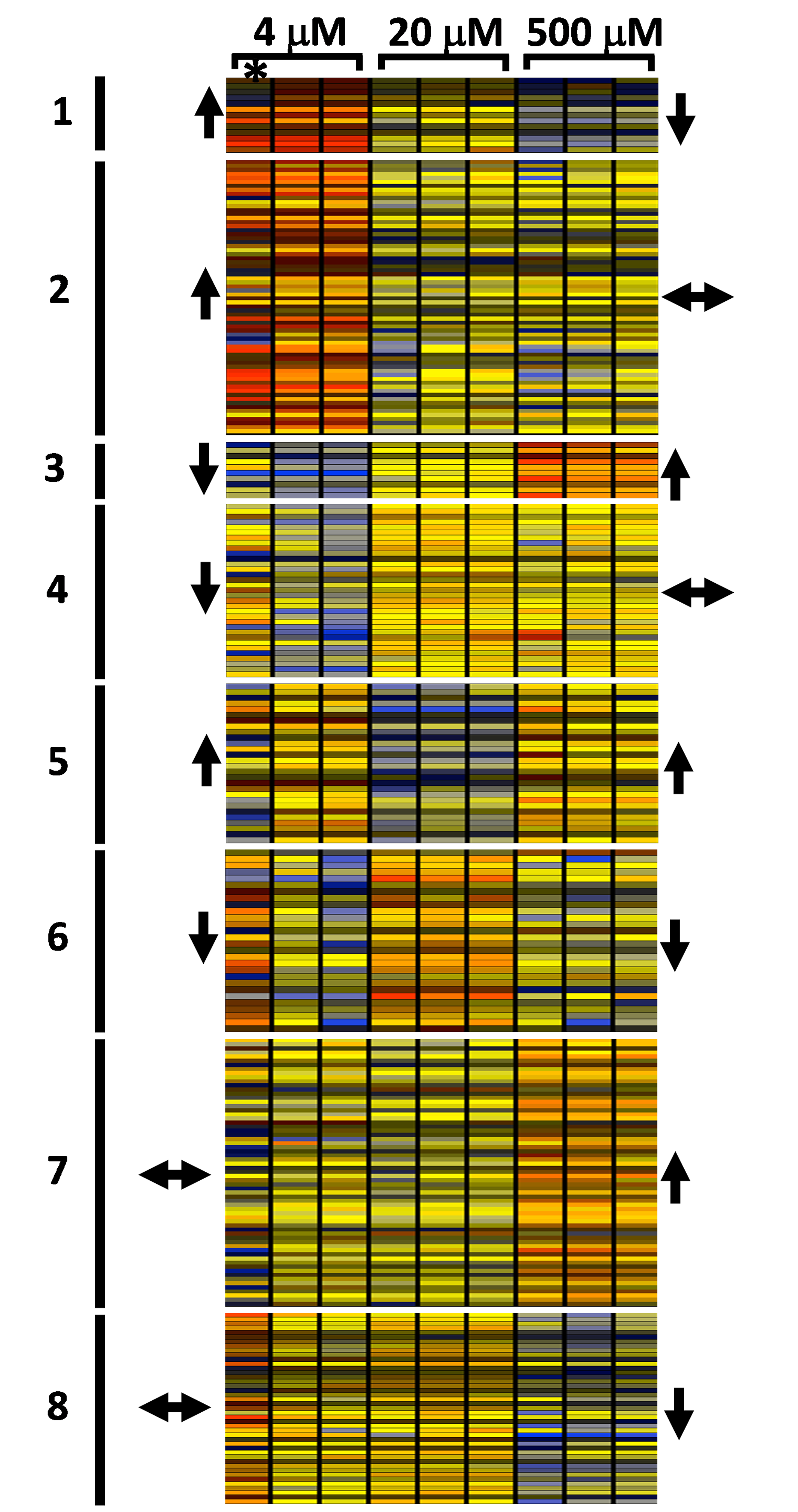

Supplement: Figure S1 — Heat map for the heme microarrays. A compilation of heat maps generated following normalization of the data (see Figure 2 legend) using GeneSpring (v7.2) for each category of the 288 hrgs with data from all nine chips represented. The up and down arrows indicate upregulation or downregulation in 4 or 500 µM heme when compared to 20 µM heme. Yellow represents no change in signal intensity, blue indicates a decrease, and red indicates an increase in signal intensity. The data from the first replicate sample from 4 µM heme, indicated with an asterisk at the top of the column, were inconsistent with the data from the other two biological replicates as determined by both principal components analysis and K-means clustering of the data. (2.43 MB TIF) [file pgen.1001044.s001.tif]

A

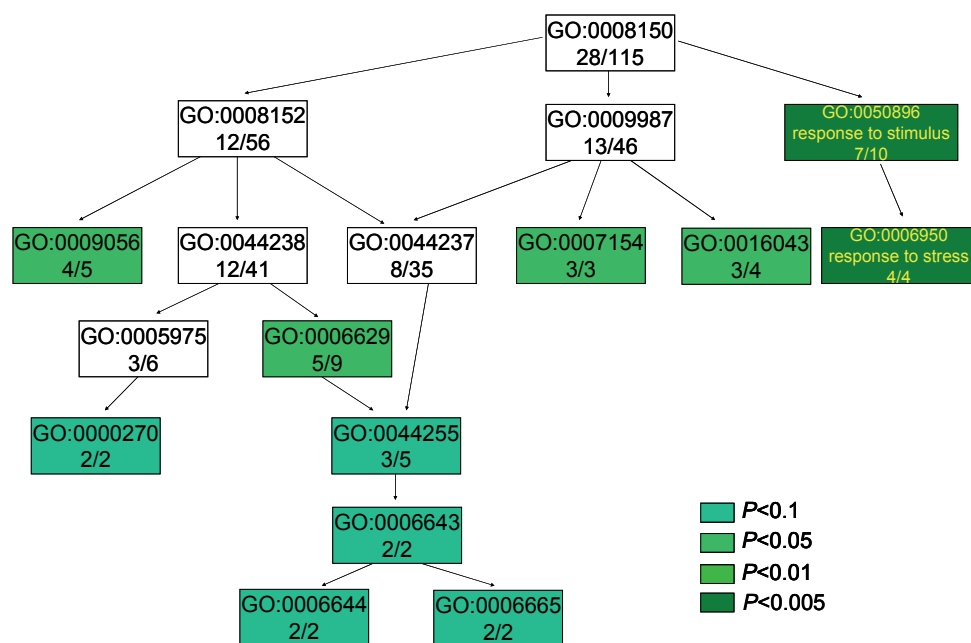

**B**

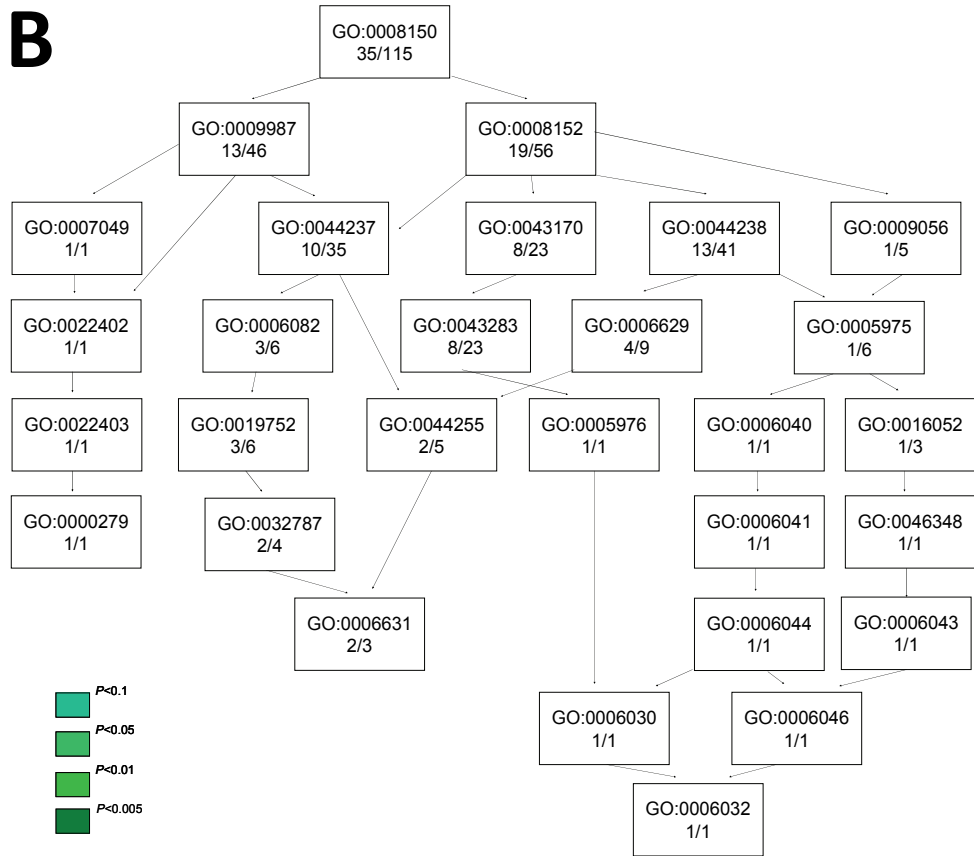

C

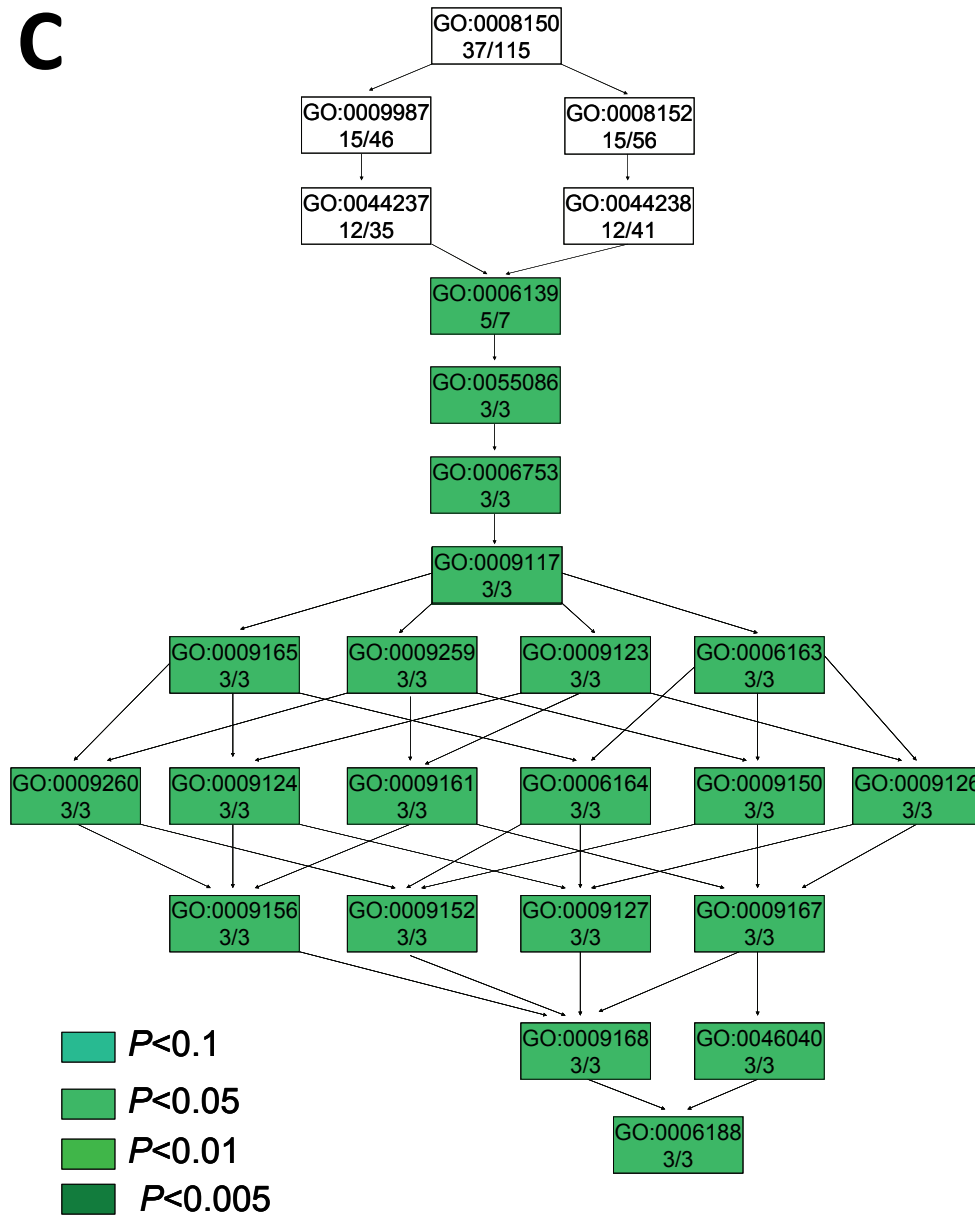

Supplement: Figure S2 — Gene ontology (GO) enrichment analysis of heme-responsive genes. (A) hrgs downregulated at 4 µM heme. (B) hrgs upregulated at 500 µM heme. (C) hrgs downregulated at 500 µM heme. Of the 288 hrgs identified in the study, 115 were annotated with a biological process. Genes were analyzed using the Fisher's exact test and the topGO package from R. The most significant GO terms and their associated parent terms were used to construct a hierarchical graph such that the specificity of the terms increased as we moved from top to bottom. The text in each rectangle provides the GO ID and the ratio of the number of genes annotated with the GO term in the tested subset to that in the total gene set. The shade of green of each rectangle corresponds to the significance of the GO result. Full GO terms are provided solely for genes with P<0.005. The complete table of P-values and a full description of the GO term associated with each gene can be found in Tables S7, S8, and S9. (0.08 MB PDF) [file pgen.1001044.s002.pdf]

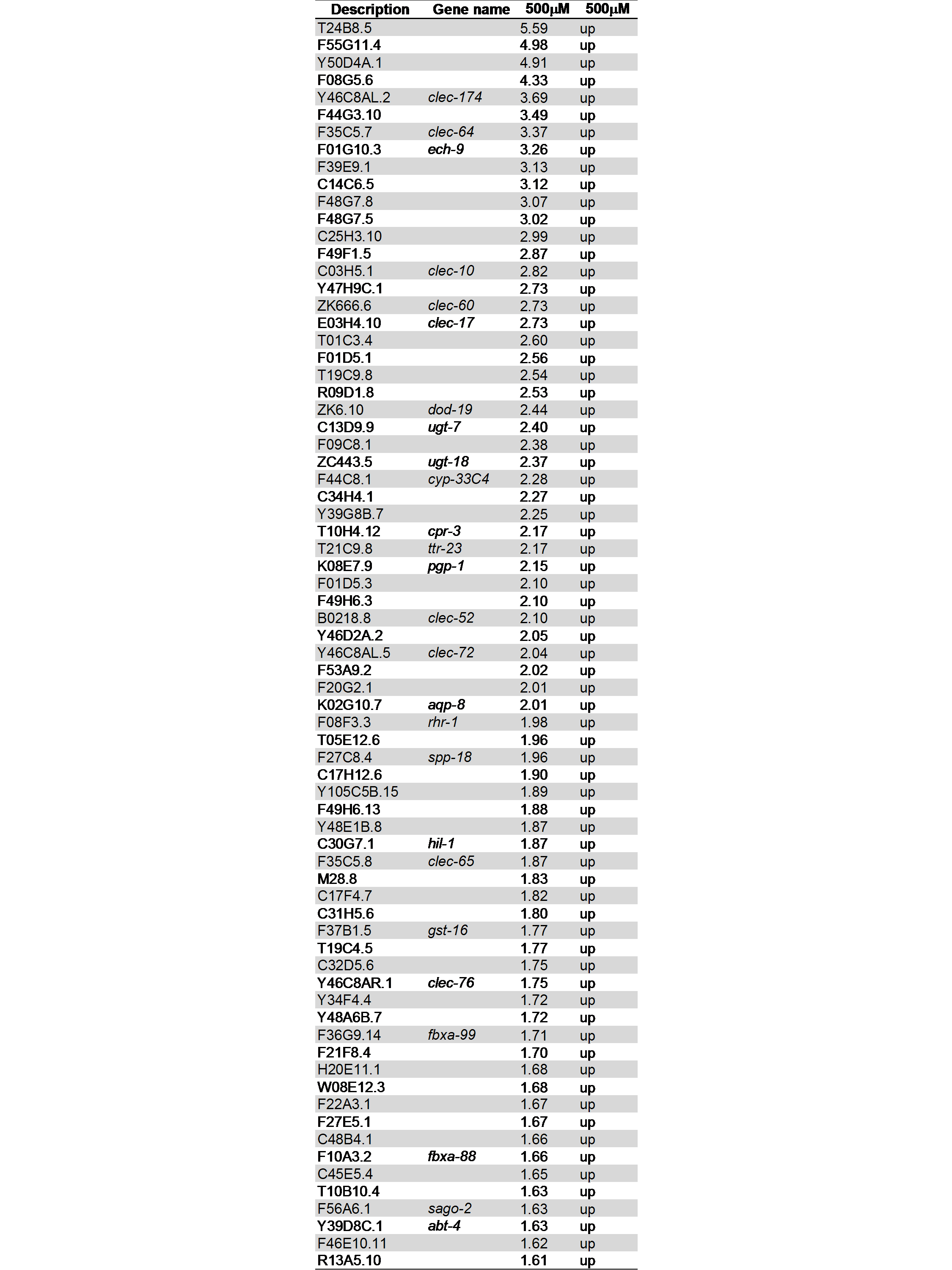

Supplement: Table S3 — Heme-responsive genes whose expression is upregulated greater than 1.6 fold in worms grown at 500 µM heme. The gene ID (description), gene name, and amount of change at 500 µM compared to the control (20 µM) are provided for each gene whose expression increased at 500 µM. (0.73 MB TIF) [file pgen.1001044.s005.tif]

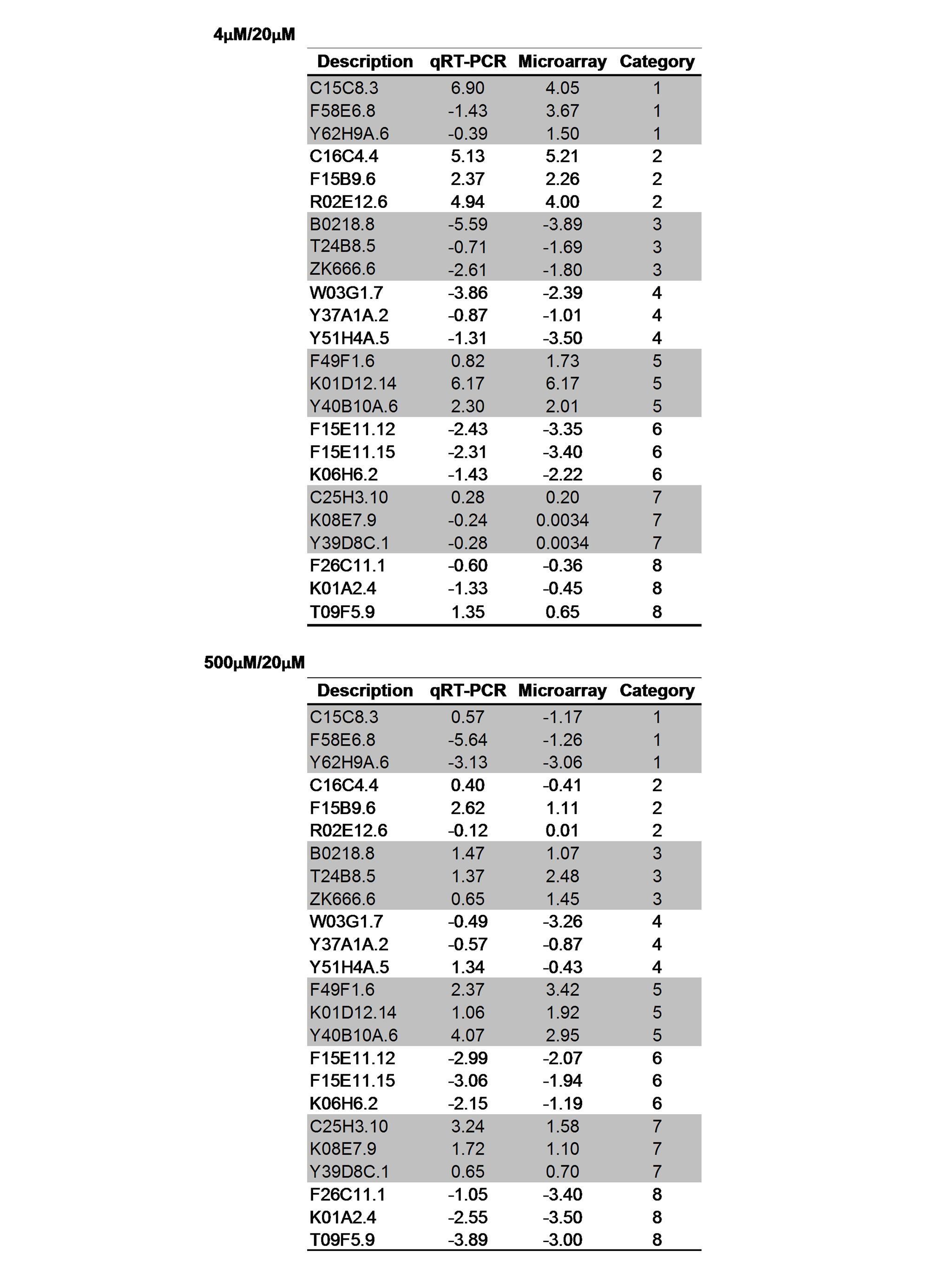

Supplement: Table S4 — Heme-responsive genes used to corroborate the microarray results. Three genes were selected from each of the eight categories designed to show whether the expression of a gene increased, decreased, or did not change at a given heme concentration compared to the 20 µM control. (1.15 MB TIF) [file pgen.1001044.s006.tif]

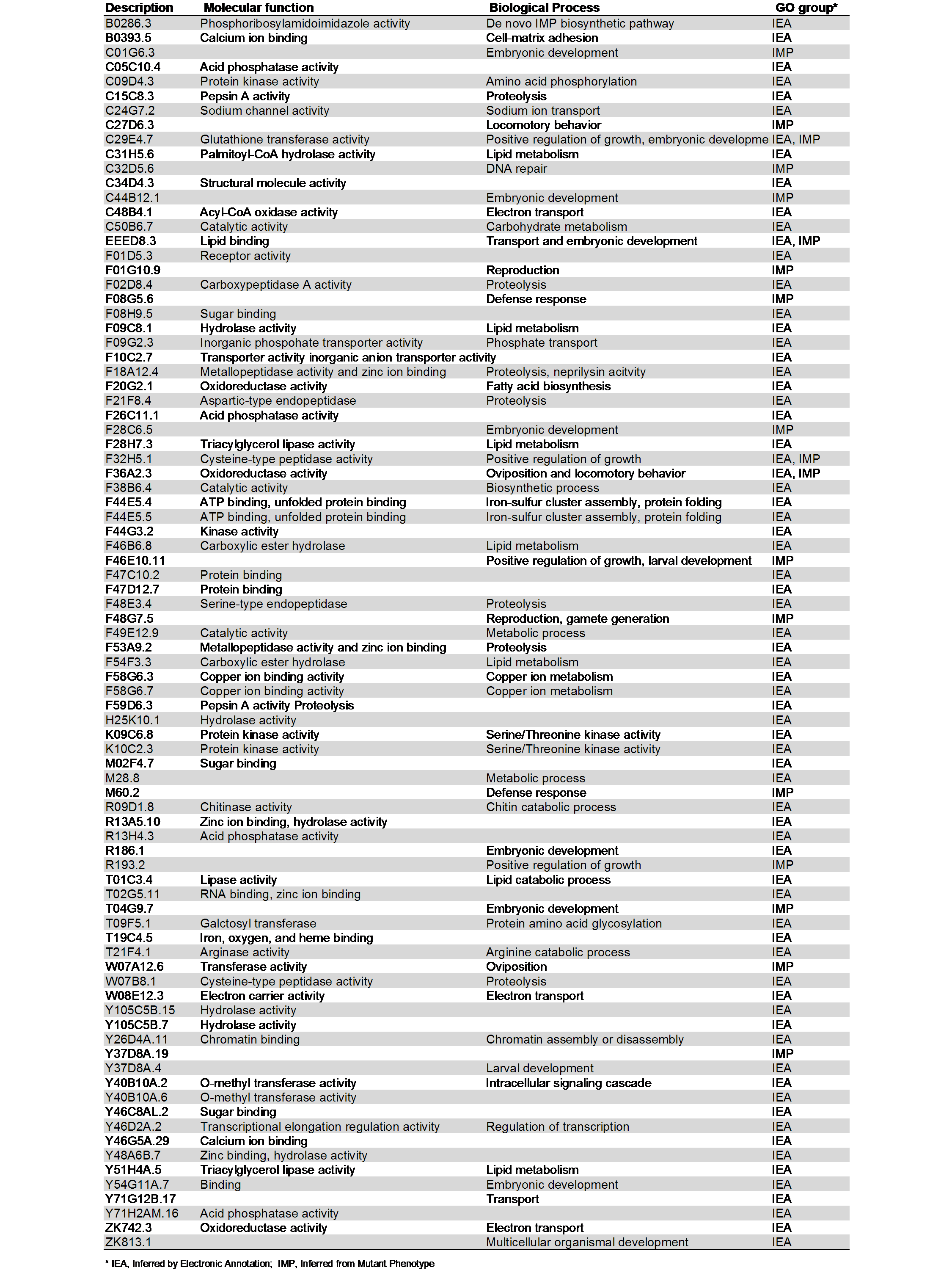

Supplement: Table S5 — Heme-responsive genes with known Gene Ontology terms. Of the 288 hrgs whose expression changed significantly in response to heme, the results of a gene ontology analysis were used to assign a known biological process and molecular function to 63 genes. (1.02 MB TIF) [file pgen.1001044.s007.tif]

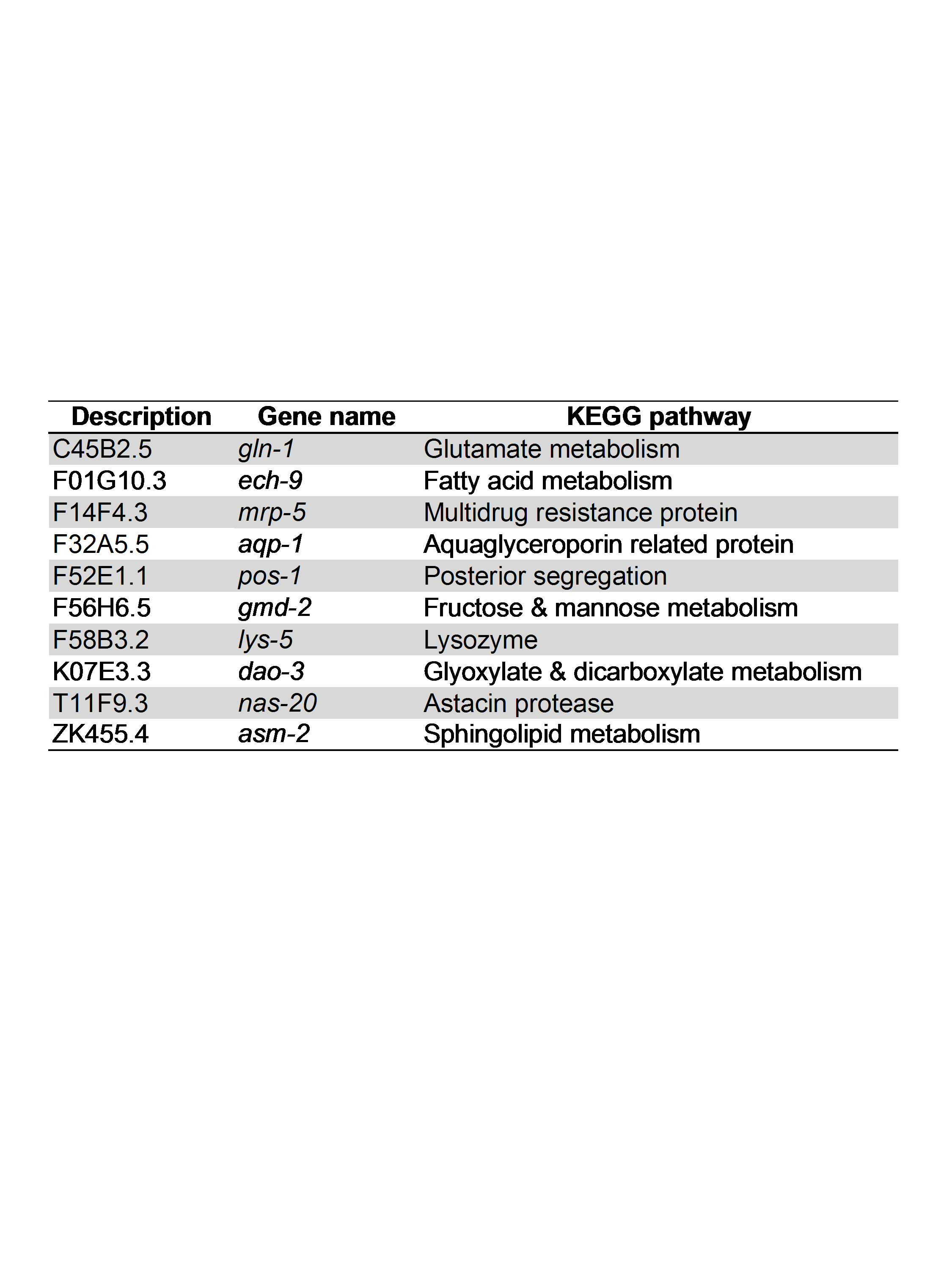

Supplement: Table S10 — Heme-responsive genes assigned to a biological pathway by KEGG analysis. The algorithms available on the Kyoto Encyclopedia of Genes and Genomes website were used to make functional predictions for each of the 288 hrgs identified in the microarray. Ten hrgs were mapped to KEGG pathways. (0.59 MB TIF) [file pgen.1001044.s012.tif]

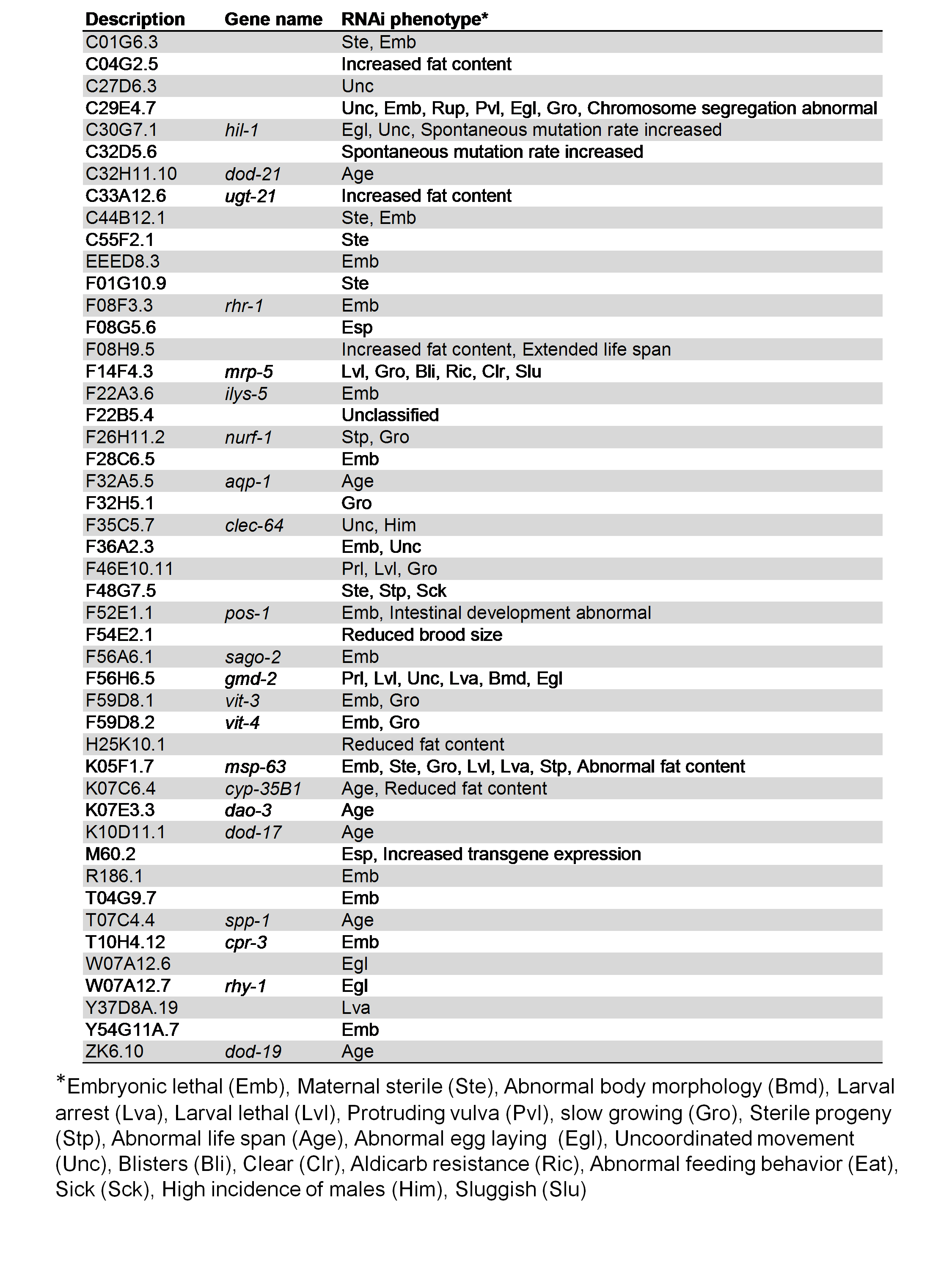

Supplement: Table S11 — Previously reported RNAi phenotypes of heme-responsive genes. Phenotypes observed when hrgs were knocked down in experiments performed by other laboratories and compiled on Wormbase. (0.85 MB TIF) [file pgen.1001044.s013.tif]

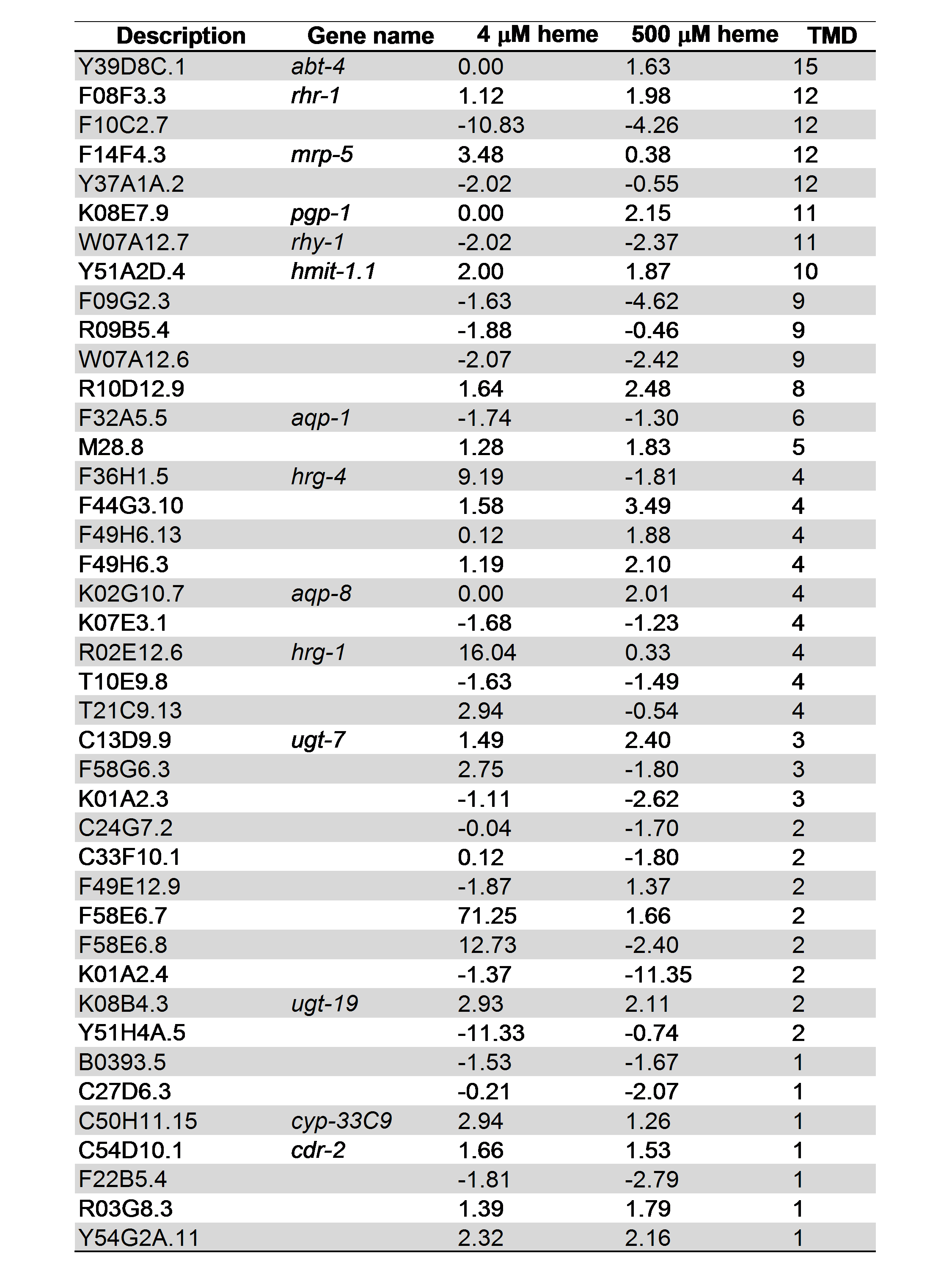

Supplement: Table S12 — Heme-responsive genes with predicted TMDs. Worm protein sequences obtained from Wormbase were analyzed using TMHMM 2.0 and SOSUI to identify 41 proteins with putative hydrophobic membrane-spanning domains (TMDs). The 41 genes with putative TMDs have been arranged according to the number of TMDs. The change in levels of gene expression at 4 and 500 µM heme is indicated. Negative fold change implies down regulation. (0.74 MB TIF) [file pgen.1001044.s014.tif]
