# Supplementary material for: Genome-Wide Analysis Reveals Novel Genes Essential for Heme Homeostasis in Caenorhabditis elegans
Source: PLoS Genet. 2010 Jul 29;6(7):e1001044. doi: 10.1371/journal.pgen.1001044 (PMC2912396; doi:10.1371/journal.pgen.1001044)
Supplement: Table S1 — The 288 heme-responsive genes identified by the microarray. Data was collected using the Affymetrix C. elegans whole genome array and analyzed by both Affymetrix MAS 5.0 software and RMA. Each entry in the table represents a gene whose expression changed at least 1.6 fold at one or both of the experimental heme concentrations. The table has six columns for each hrg. The “Description” column lists the unique Gene ID assigned by Wormbase to every gene in the C. elegans genome. The “Gene name” column provides the name of a gene, when one has been assigned. The first “4 µM” column gives the value of the change of expression of each gene, and the second “4 µM” column indicates whether the gene expression was increased (up) or decreased (down). If the column is blank, then the change was less than 1.6-fold. The pattern for the “500 µM” columns is the same as for the “4 µM” columns. (0.10 MB PDF) [file pgen.1001044.s003.pdf]

Description	Gene name	4μM	500μM	4μM	500μM
C08F11.11		1.62	-2.02	up	down
C15C8.3		16.57	-2.25	up	down
C44B7.5		3.78	-2.14	up	down
EEED8.3		2.00	-5.55	up	down
F36H1.5	*hrg-4*	9.19	-1.81	up	down
F57C2.4		2.94	-2.64	up	down
F58E6.8		12.73	-2.40	up	down
F58G6.3		2.75	-1.80	up	down
F59D8.1	*vit-3*	4.35	-1.74	up	down
K09F5.2	*vit-1*	1.65	-1.80	up	down
W07B8.1		5.86	-1.91	up	down
Y62H9A.6		2.83	-8.36	up	down
ZK813.1		3.74	-3.37	up	down
C01G6.3		3.62	-0.34	up	
C04F6.1		1.74	-0.47	up	
C05E7.2		1.74	1.35	up	
C08B6.1		1.84	0.19	up	
C10G8.4		4.20	1.51	up	
C16C4.4	*math-14*	36.92	-1.33	up	
C25A1.8	*clec-87*	2.15	0.35	up	
C29E4.7	*gsto-1*	12.15	-1.32	up	
C31C9.1	*tag-10*	4.59	-0.49	up	
C33A12.6	*ugt-21*	2.87	1.14	up	
C33H5.13		1.62	1.39	up	
C42D4.3		1.93	1.39	up	
C42D8.2		2.62	-0.71	up	
C44B12.1		3.14	-1.03	up	
C50H11.15	*cyp-33C9*	2.94	1.26	up	
C54D10.1	*cdr-2*	1.66	1.53	up	
C54F6.14	*ftn-1*	1.66	0.88	up	
D1054.10		3.28	-1.44	up	
F07C4.2	*clec-45*	3.51	0.48	up	
F07C4.9	*clec-46*	2.93	0.53	up	
F07F6.5	*dct-5*	1.68	1.35	up	
F11G11.3	*gst-6*	1.88	1.59	up	
F14F4.3	*mrp-5*	3.48	0.38	up	
F15B9.6		4.78	1.49	up	
F17E9.4		3.78	-0.79	up	
F18A12.4		10.87	0.15	up	
F26F12.1		1.72	-0.54	up	
F32H5.1		3.14	0.36	up	
F32H5.3		2.00	1.49	up	
F35B3.4		2.14	1.48	up	
F36A2.3		1.93	0.33	up	
F38A3.1	*col-81*	1.68	-0.48	up	
F41F3.4	*col-139*	1.61	-0.25	up	
F42G2.2		2.30	1.18	up	
F44G3.2		1.80	-1.18	up	
F47C10.2	*btb-21*	2.07	1.39	up	
F48E3.4		1.80	-0.79	up	
F52E1.1	*pos-1*	2.00	-0.44	up	
F58E6.4		2.20	0.13	up	
F59D6.3		2.22	-0.66	up	
F59D8.2	*vit-4*	5.80	-1.34	up	
H23N18.1	*ugt-13*	1.77	-0.17	up	
K01D12.9		2.07	0.85	up	
K02B9.1	*meg-1*	3.07	-1.10	up	
K07H8.6	*vit-6*	4.45	-0.88	up	
K10B2.2		2.84	0.25	up	
K10D11.1	*dod-17*	1.68	0.62	up	
M02H5.4	*nhr-202*	2.30	1.24	up	
R02E12.6	*hrg-1*	16.04	0.33	up	
R06C7.4	*cpg-3*	1.87	0.20	up	
R193.2		2.82	-0.35	up	
T02G5.11		1.96	0.33	up	
T04G9.7		2.94	-0.63	up	
T05A1.2	*col-122*	1.81	-0.74	up	
T11F9.3	*nas-20*	1.87	-0.88	up	
T18H9.1	*grd-6*	1.87	1.86	up	
T21C9.13		2.94	-0.54	up	
T21F4.1		1.80	1.43	up	
T28C12.6		1.80	1.57	up	

Description	Gene name	4μM	500μM	4μM	500μM
W02G9.4		1.68	0.33	up	
W03G11.1	*col-181*	1.71	-0.51	up	
Y37D8A.19		4.45	-0.68	up	
Y54G11A.7		3.62	0.36	up	
Y62H9A.4		4.66	0.66	up	
Y73F8A.9	*pqn-91*	1.74	0.59	up	
ZC373.2		4.01	-0.77	up	
ZK1193.1	*col-19*	2.28	-0.22	up	
ZK742.3		2.55	1.55	up	
B0218.8	*clec-52*	-14.84	2.10	down	up
C17F4.7		-1.91	1.82	down	up
F08G5.6		-2.07	4.33	down	up
F21F8.4		-2.65	1.70	down	up
F55G11.4		-1.88	4.98	down	up
T24B8.5		-3.22	5.59	down	up
Y46C8AL.2	*clec-174*	-1.76	3.69	down	up
Y46C8AL.5	*clec-72*	-1.69	2.04	down	up
Y46D2A.2		-1.76	2.05	down	up
ZK666.6	*clec-60*	-3.49	2.73	down	up
D1086.3		-1.87	-1.41	down	
F28H7.3		-1.68	0.52	down	
F32A5.5	*aqp-1*	-1.74	-1.30	down	
F46B6.8		-2.94	0.49	down	
F47G9.3	*cutl-18*	-1.94	-1.29	down	
F49E12.9		-1.87	1.37	down	
F54D5.8	*dnj-13*	-1.68	-1.42	down	
F54F3.3		-3.26	1.60	down	
F58B3.3	*lys-6*	-2.00	-1.02	down	
F59B10.5		-1.62	-1.39	down	
H40L08.2		-2.23	0.38	down	
K07E3.1		-1.68	-1.23	down	
K10C2.3		-3.14	-1.49	down	
M60.2		-2.02	0.36	down	
R09B5.4	*fpn-1.2*	-1.88	-0.46	down	
R13H4.3		-1.62	0.36	down	
T07C4.4	*spp-1*	-1.80	-0.44	down	
T08A9.8	*spp-4*	-1.80	-0.41	down	
T10E9.8		-1.63	-1.49	down	
T16G1.7		-2.07	-0.36	down	
W03G1.7		-5.25	-0.10	down	
W04E12.8	*clec-50*	-1.68	1.13	down	
Y19D10B.7		-2.01	-0.31	down	
Y37A1A.2		-2.02	-0.55	down	
Y37D8A.4		-1.75	-1.52	down	
Y46G5A.29		-1.75	-1.58	down	
Y46H3A.2	*hsp-16.41*	-1.80	-0.08	down	
Y46H3A.3	*hsp-16.2*	-1.74	-0.09	down	
Y51H4A.5		-11.33	-0.74	down	
Y54F10AM.6		-1.75	-0.64	down	
Y5H2A.1		-2.02	-1.40	down	
ZK377.1	*wrt-6*	-1.96	-1.59	down	
ZK455.4	*asm-2*	-1.80	1.13	down	
C04H5.7		2.80	5.78	up	up
C24F3.3	*nas-12*	3.05	1.83	up	up
C32H11.10	*dod-21*	2.42	7.04	up	up
C34H4.2		1.81	1.86	up	up
F02D8.4		1.76	1.70	up	up
F08F8.5	*numr-1*	7.66	8.23	up	up
F21H7.1	*gst-22*	1.63	2.42	up	up
F35C5.9	*clec-66*	1.93	2.08	up	up
F49F1.6		3.32	10.71	up	up
F54E2.1		2.22	1.70	up	up
F58E6.7		71.25	1.66	up	up
K01D12.14	*cdr-5*	72.08	3.79	up	up
K02E2.4	*ins-35*	1.63	3.09	up	up
K08B4.3	*ugt-19*	2.93	2.11	up	up
K11H12.4		1.93	1.71	up	up
M02F4.7	*clec-265*	2.16	2.86	up	up
R10D12.9		1.64	2.48	up	up
R186.1		2.81	1.70	up	up
T05B4.3	*phat-4*	1.81	1.70	up	up
T24A11.3	*toh-1*	1.80	1.63	up	up

Description	Gene name	4μM	500μM	4μM	500μM
Y26D4A.10		1.75	2.20	up	up
Y38E10A.5	*clec-4*	2.00	2.10	up	up
Y40B10A.2		1.63	1.84	up	up
Y40B10A.6		4.04	7.74	up	up
Y51A2D.4	*hmit-1.1*	2.00	1.87	up	up
Y54G2A.11		2.32	2.16	up	up
Y75B8A.28		2.75	1.70	up	up
ZC443.6	*ugt-16*	3.28	1.91	up	up
C04G2.5		-1.70	-1.97	down	down
C14C6.3		-2.32	-2.20	down	down
E01G4.6		-2.62	-2.17	down	down
E03A3.4	*his-70*	-2.39	-2.07	down	down
F01G10.9		-1.78	-2.03	down	down
F08H9.5	*clec-227*	-1.68	-2.10	down	down
F09G2.3		-1.63	-4.62	down	down
F10C2.7		-10.83	-4.26	down	down
F15E11.12		-10.20	-4.21	down	down
F15E11.15		-10.58	-3.85	down	down
F22A3.6	*ilys-5*	-2.49	-1.87	down	down
F22B5.4		-1.81	-2.79	down	down
F28C6.5		-1.63	-1.64	down	down
F44E5.4		-7.21	-4.14	down	down
F44E5.5		-5.25	-4.16	down	down
F55C10.4		-1.87	-2.31	down	down
F56H6.5	*gmd-2*	-2.35	-4.15	down	down
F57G8.7		-2.22	-2.70	down	down
F58B3.2	*lys-5*	-2.07	-1.83	down	down
K06H6.1		-2.67	-2.22	down	down
K06H6.2		-4.67	-2.28	down	down
T22B7.3		-2.39	-1.80	down	down
W03F11.5		-1.68	-1.71	down	down
W07A12.6	*oac-54*	-2.07	-2.42	down	down
W07A12.7	*rhy-1*	-2.02	-2.37	down	down
Y105C5B.7		-3.14	-14.34	down	down
Y43D4A.5		-2.77	-1.78	down	down
Y71G12B.18		-12.25	-2.15	down	down
C03H5.1	*clec-10*	1.16	2.82		up
C13D9.9	*ugt-7*	1.49	2.40		up
C14C6.5		-1.23	3.12		up
C17H12.6		1.28	1.90		up
C25H3.10		1.15	2.99		up
C30G7.1	*hil-1*	1.23	1.87		up
C31H5.6		0.04	1.80		up
C32D5.6		1.23	1.75		up
C34H4.1		1.46	2.27		up
C45B2.5	*gln-1*	1.32	1.68		up
C45E5.4		1.32	1.65		up
C48B4.1		-1.20	1.66		up
E03H4.10	*clec-17*	1.16	2.73		up
F01D5.1		-1.11	2.56		up
F01D5.3		-1.52	2.10		up
F01G10.3	*ech-9*	-0.04	3.26		up
F08F3.3	*rhr-1*	1.12	1.98		up
F09C8.1		-1.47	2.38		up
F10A3.2	*fbxa-88*	-1.11	1.66		up
F20G2.1		1.29	2.01		up
F22A3.1		-0.22	1.67		up
F25A2.1		1.58	4.91		up
F27C8.4	*spp-18*	-1.52	1.96		up
F27E5.1		1.28	1.67		up
F35C5.7	*clec-64*	0.43	3.37		up
F35C5.8	*clec-65*	1.32	1.87		up
F36G9.14	*fbxa-99*	1.00	1.71		up
F37B1.5	*gst-16*	1.37	1.77		up
F39E9.1		1.37	3.13		up
F44C8.1	*cyp-33C4*	0.28	2.28		up
F44G3.10		1.58	3.49		up
F46E10.11		1.19	1.62		up
F48G7.5		1.11	3.02		up
F48G7.8		0.16	3.07		up
F49F1.5		-1.28	2.87		up
F49H6.13		0.12	1.88		up

Description	Gene name	4μM	500μM	4μM	500μM
F49H6.3		1.19	2.10		up
F53A9.2		1.21	2.02		up
F56A6.1	*sago-2*	-1.15	1.63		up
H20E11.1		1.12	1.68		up
K02G10.7	*aqp-8*	0.00	2.01		up
K08E7.9	*pgp-1*	0.00	2.15		up
M28.8		1.28	1.83		up
R03G8.3		1.39	1.79		up
R09D1.8		0.44	2.53		up
R13A5.10		0.13	1.61		up
T01C3.4		-1.28	2.60		up
T03F7.7		1.42	1.64		up
T05E12.6		1.23	1.96		up
T10B10.4		1.23	1.63		up
T10H4.12	*cpr-3*	1.28	2.17		up
T19C4.5		-0.08	1.77		up
T19C9.8		1.53	2.54		up
T21C9.8	*ttr-23*	1.47	2.17		up
W08E12.3		1.42	1.68		up
Y105C5B.15		-1.37	1.89		up
Y34F4.4		1.46	1.72		up
Y39D8C.1	*abt-4*	0.00	1.63		up
Y39G8B.7		-1.46	2.25		up
Y46C8AR.1	*clec-76*	1.53	1.75		up
Y47H9C.1		-1.07	2.73		up
Y48A6B.7		-1.11	1.72		up
Y48E1B.8		-0.04	1.87		up
ZC443.5	*ugt-18*	1.21	2.37		up
ZK6.10	*dod-19*	1.15	2.44		up
B0286.3		-0.12	-1.97		down
B0393.5		-1.53	-1.67		down
C05C10.4		-1.28	-2.14		down
C06B3.7		1.21	-2.21		down
C07E3.10		-1.57	-1.69		down
C09D4.3		-1.24	-1.67		down
C24G7.2		-0.04	-1.70		down
C27D6.3		-0.21	-2.07		down
C33F10.1		0.12	-1.80		down
C34D4.3		1.37	-3.61		down
C50B6.7		1.23	-2.53		down
C55F2.1		-0.43	-2.17		down
F17C8.7		-0.30	-1.91		down
F26B1.1		1.35	-2.42		down
F26C11.1		-1.28	-10.56		down
F26H11.2	*nurf-1*	-1.17	-1.79		down
F28D1.5	*thn-2*	-1.28	-3.51		down
F32B6.4		-0.09	-1.66		down
F36A4.2		0.04	-2.75		down
F37B1.8	*gst-19*	1.39	-2.30		down
F38B6.4		-1.07	-2.03		down
F47C10.6	*ugt-32*	-1.46	-2.11		down
F47D12.7		-0.04	-3.49		down
F59C6.6	*nlp-4*	-1.52	-2.23		down
H25K10.1		-1.57	-1.74		down
K01A2.3		-1.11	-2.62		down
K01A2.4		-1.37	-11.35		down
K05F1.7	*msp-63*	1.49	-1.80		down
K07C6.4	*cyp-35B1*	-1.55	-6.77		down
K07E3.3	*dao-3*	-1.37	-1.73		down
K09C6.8		-0.40	-8.49		down
M02D8.4		-1.42	-1.75		down
T09F5.1		-1.52	-1.71		down
T09F5.9	*clec-47*	1.57	-8.01		down
Y11D7A.5		-1.53	-1.72		down
Y53F4B.32	*gst-29*	0.04	-1.97		down
Y59H11AM.3		1.57	-4.39		down
Y67A6A.2		-1.37	-1.75		down
Y71G12B.17		0.00	-1.73		down
Y71H2AM.16		-1.52	-2.15		down
ZK105.1		-0.16	-1.71		down
ZK520.5	*cyn-2*	-0.08	-4.81		down
ZK970.7		-0.08	-1.67		down
