# Supplementary material for: Genome-Wide Analysis Reveals Novel Genes Essential for Heme Homeostasis in Caenorhabditis elegans
Source: PLoS Genet. 2010 Jul 29;6(7):e1001044. doi: 10.1371/journal.pgen.1001044 (PMC2912396; doi:10.1371/journal.pgen.1001044)
Supplement: Table S2 — Heme-responsive genes whose expression is upregulated greater than 1.6 fold in worms grown at 4 µM heme. The gene ID (description), gene name, and amount of change at 4 µM compared to the control (20 µM) are provided for each gene whose expression increased at 4 µM. (0.08 MB PDF) [file pgen.1001044.s004.pdf]

Description	Gene name	4μM	4μM
K01D12.14	*cdr-5*	72.08	up
F58E6.7		71.25	up
C16C4.4	*math-14*	36.92	up
C15C8.3		16.57	up
R02E12.6	*hrg-1*	16.04	up
F58E6.8		12.73	up
C29E4.7	*gsto-1*	12.15	up
F18A12.4		10.87	up
F36H1.5	*hrg-4*	9.19	up
F08F8.5	*numr-1*	7.66	up
W07B8.1		5.86	up
F59D8.2	*vit-4*	5.80	up
F15B9.6		4.78	up
Y62H9A.4		4.66	up
C31C9.1	*tag-10*	4.59	up
Y37D8A.19		4.45	up
K07H8.6	*vit-6*	4.45	up
F59D8.1	*vit-3*	4.35	up
C10G8.4		4.20	up
Y40B10A.6		4.04	up
ZC373.2		4.01	up
C44B7.5		3.78	up
F17E9.4		3.78	up
ZK813.1		3.74	up
C01G6.3		3.62	up
Y54G11A.7		3.62	up
F07C4.2	*clec-45*	3.51	up
F14F4.3	*mrp-5*	3.48	up
F49F1.6		3.32	up
ZC443.6	*ugt-16*	3.28	up
D1054.10		3.28	up
C44B12.1		3.14	up
F32H5.1		3.14	up
K02B9.1	*meg-1*	3.07	up
C24F3.3	*nas-12*	3.05	up
F57C2.4		2.94	up
C50H11.15	*cyp-33C9*	2.94	up
T04G9.7		2.94	up
T21C9.13		2.94	up
K08B4.3	*ugt-19*	2.93	up
F07C4.9	*clec-46*	2.93	up
C33A12.6	*ugt-21*	2.87	up
K10B2.2		2.84	up
Y62H9A.6		2.83	up
R193.2		2.82	up
R186.1		2.81	up
C04H5.7		2.80	up
Y75B8A.28		2.75	up
F58G6.3		2.75	up
C42D8.2		2.62	up
ZK742.3		2.55	up
C32H11.10	*dod-21*	2.42	up
Y54G2A.11		2.32	up
F42G2.2		2.30	up
M02H5.4	*nhr-202*	2.30	up

Description	Gene name	4μM	4μM
ZK1193.1	*col-19*	2.28	up
F54E2.1		2.22	up
F59D6.3		2.22	up
F58E6.4		2.20	up
M02F4.7	*clec-265*	2.16	up
C25A1.8	*clec-87*	2.15	up
F35B3.4		2.14	up
F47C10.2	*btb-21*	2.07	up
K01D12.9		2.07	up
Y38E10A.5	*clec-4*	2.00	up
Y51A2D.4	*hmit-1.1*	2.00	up
F32H5.3		2.00	up
EEED8.3		2.00	up
F52E1.1	*pos-1*	2.00	up
T02G5.11		1.96	up
F35C5.9	*clec-66*	1.93	up
K11H12.4		1.93	up
C42D4.3		1.93	up
F36A2.3		1.93	up
F11G11.3	*gst-6*	1.88	up
T18H9.1	*grd-6*	1.87	up
R06C7.4	*cpg-3*	1.87	up
T11F9.3	*nas-20*	1.87	up
C08B6.1		1.84	up
C34H4.2		1.81	up
T05B4.3	*phat-4*	1.81	up
T05A1.2	*col-122*	1.81	up
T24A11.3	*toh-1*	1.80	up
F44G3.2		1.80	up
F48E3.4		1.80	up
T21F4.1		1.80	up
T28C12.6		1.80	up
H23N18.1	*ugt-13*	1.77	up
F02D8.4		1.76	up
Y26D4A.10		1.75	up
C05E7.2		1.74	up
Y73F8A.9	*pqn-91*	1.74	up
C04F6.1		1.74	up
F26F12.1		1.72	up
W03G11.1	*col-181*	1.71	up
F07F6.5	*dct-5*	1.68	up
K10D11.1	*dod-17*	1.68	up
W02G9.4		1.68	up
F38A3.1	*col-81*	1.68	up
C54D10.1	*cdr-2*	1.66	up
C54F6.14	*ftn-1*	1.66	up
K09F5.2	*vit-1*	1.65	up
R10D12.9		1.64	up
F21H7.1	*gst-22*	1.63	up
K02E2.4	*ins-35*	1.63	up
Y40B10A.2		1.63	up
C33H5.13		1.62	up
C08F11.11		1.62	up
F41F3.4	*col-139*	1.61	up
