# Supplementary material for: Genome-Wide Analysis Reveals Novel Genes Essential for Heme Homeostasis in Caenorhabditis elegans
Source: PLoS Genet. 2010 Jul 29;6(7):e1001044. doi: 10.1371/journal.pgen.1001044 (PMC2912396; doi:10.1371/journal.pgen.1001044)
Supplement: Table S6 — Gene Ontology analysis of heme-responsive genes upregulated at 4 µM heme. Each GO ID is assigned a unique function or association. Both are listed here, even if the GO ID was not used in the GO analysis figure. Green shading indicates that term was included in the corresponding GO enrichment figures. (0.06 MB PDF) [file pgen.1001044.s008.pdf]

Term	Annotated	Significant	Expected	Rank in classic Fisher	classic Fisher	elimFisher
lipid transport	6	6	2.3	1	0.0025	0.0025
proteolysis	15	11	5.74	2	0.0037	0.0037
embryonic development ending in birth or...	17	12	6.5	3	0.0038	0.0038
embryonic development	18	12	6.89	4	0.008	1
developmental process	37	20	14.16	5	0.0145	0.2978
multicellular organismal development	35	18	13.39	6	0.044	0.575
protein metabolic process	19	11	7.27	7	0.049	1
cellular protein metabolic process	19	11	7.27	8	0.049	1
cellular biopolymer metabolic process	22	12	8.42	9	0.0676	0.9457
cellular macromolecule metabolic process	22	12	8.42	10	0.0676	0.9457
multicellular organismal process	39	19	14.92	11	0.074	0.6319
macromolecule metabolic process	23	12	8.8	12	0.0986	0.965
biopolymer metabolic process	23	12	8.8	13	0.0986	0.965
regulation of growth rate	19	10	7.27	14	0.1252	0.1252
positive regulation of growth rate	19	10	7.27	15	0.1252	0.1252
biological regulation	24	12	9.18	16	0.1373	0.1373
regulation of growth	20	10	7.65	17	0.1744	0.1744
positive regulation of growth	20	10	7.65	18	0.1744	0.1744
positive regulation of biological proces...	20	10	7.65	19	0.1744	0.1744
growth	23	11	8.8	20	0.2065	0.2065
regulation of biological process	23	11	8.8	21	0.2065	0.2065
anatomical structure development	7	4	2.68	22	0.251	0.251
anatomical structure morphogenesis	5	3	1.91	23	0.2843	0.2843
transport	17	8	6.5	24	0.292	0.9476
localization	17	8	6.5	25	0.292	0.9476
establishment of localization	17	8	6.5	26	0.292	0.9476
body morphogenesis	3	2	1.15	27	0.3257	0.3257
transition metal ion transport	1	1	0.38	28	0.3826	0.3826
cell fate specification	1	1	0.38	29	0.3826	0.3826
morphogenesis of an epithelium	1	1	0.38	30	0.3826	0.3826
iron ion transport	1	1	0.38	31	0.3826	0.3826
cellular ion homeostasis	1	1	0.38	32	0.3826	0.3826
cellular iron ion homeostasis	1	1	0.38	33	0.3826	0.3826
pattern specification process	1	1	0.38	34	0.3826	0.3826
protein-based cuticle development	1	1	0.38	35	0.3826	0.3826
embryonic pattern specification	1	1	0.38	36	0.3826	0.3826
di-, tri-valent inorganic cation transpo...	1	1	0.38	37	0.3826	0.3826
cellular homeostasis	1	1	0.38	38	0.3826	0.3826
cellular cation homeostasis	1	1	0.38	39	0.3826	0.3826
cellular di-, tri-valent inorganic catio...	1	1	0.38	40	0.3826	0.3826
cell differentiation	1	1	0.38	41	0.3826	0.3826
collagen and cuticulin-based cuticle dev...	1	1	0.38	42	0.3826	0.3826
regulation of locomotion	1	1	0.38	43	0.3826	0.3826
positive regulation of locomotion	1	1	0.38	44	0.3826	0.3826
cuticle development	1	1	0.38	45	0.3826	0.3826
homeostatic process	1	1	0.38	46	0.3826	0.3826
cell fate commitment	1	1	0.38	47	0.3826	0.3826
cellular developmental process	1	1	0.38	48	0.3826	0.3826
chemical homeostasis	1	1	0.38	49	0.3826	0.3826
ion homeostasis	1	1	0.38	50	0.3826	0.3826
di-, tri-valent inorganic cation homeost...	1	1	0.38	51	0.3826	0.3826
iron ion homeostasis	1	1	0.38	52	0.3826	0.3826
cation homeostasis	1	1	0.38	53	0.3826	0.3826
cellular chemical homeostasis	1	1	0.38	54	0.3826	0.3826
transmembrane transport	1	1	0.38	55	0.3826	0.3826
regulation of biological quality	1	1	0.38	56	0.3826	0.3826
aging	11	5	4.21	57	0.4171	0.4171
determination of adult life span	11	5	4.21	58	0.4171	0.4171
multicellular organismal aging	11	5	4.21	59	0.4171	0.4171
locomotion	11	5	4.21	60	0.4171	0.4171
reproductive developmental process	2	1	0.77	61	0.6209	0.6209
cation transport	2	1	0.77	62	0.6209	0.6209
sex differentiation	2	1	0.77	63	0.6209	0.6209
metal ion transport	2	1	0.77	64	0.6209	0.6209
hermaphrodite genitalia development	2	1	0.77	65	0.6209	0.6209
organ development	2	1	0.77	66	0.6209	0.6209
system development	2	1	0.77	67	0.6209	0.6209
genitalia development	2	1	0.77	68	0.6209	0.6209
nematode larval development	5	2	1.91	69	0.6366	0.6366
larval development	5	2	1.91	70	0.6366	0.6366
post-embryonic development	5	2	1.91	71	0.6366	0.6366
behavior	6	2	2.3	72	0.7455	0.7455

Term	Annotated	Significant	Expected	Rank in classic Fisher	classic Fisher	elimFisher
oviposition	6	2	2.3	73	0.7455	0.7455
reproductive behavior	6	2	2.3	74	0.7455	0.7455
multicellular organism reproduction	6	2	2.3	75	0.7455	0.7455
reproductive behavior in a multicellular...	6	2	2.3	76	0.7455	0.7455
reproductive process in a multicellular ...	6	2	2.3	77	0.7455	0.7455
transcription	3	1	1.15	78	0.7685	0.7685
transcription, DNA-dependent	3	1	1.15	79	0.7685	0.7685
regulation of transcription, DNA-depende...	3	1	1.15	80	0.7685	0.7685
ion transport	3	1	1.15	81	0.7685	0.7685
regulation of biosynthetic process	3	1	1.15	82	0.7685	0.7685
regulation of gene expression	3	1	1.15	83	0.7685	0.7685
regulation of macromolecule biosynthetic...	3	1	1.15	84	0.7685	0.7685
RNA metabolic process	3	1	1.15	85	0.7685	0.7685
regulation of nucleobase, nucleoside, nu...	3	1	1.15	86	0.7685	0.7685
regulation of metabolic process	3	1	1.15	87	0.7685	0.7685
regulation of cellular metabolic process	3	1	1.15	88	0.7685	0.7685
regulation of cellular biosynthetic proc...	3	1	1.15	89	0.7685	0.7685
RNA biosynthetic process	3	1	1.15	90	0.7685	0.7685
regulation of transcription	3	1	1.15	91	0.7685	0.7685
regulation of RNA metabolic process	3	1	1.15	92	0.7685	0.7685
regulation of macromolecule metabolic pr...	3	1	1.15	93	0.7685	0.7685
metabolic process	56	20	21.43	94	0.77	0.9863
cellular metabolic process	35	12	13.39	95	0.7839	0.9999
reproduction	13	4	4.97	96	0.8125	0.8125
reproductive process	7	2	2.68	97	0.8258	0.8258
gene expression	4	1	1.53	98	0.8595	0.8595
cellular biopolymer biosynthetic process	4	1	1.53	99	0.8595	0.8595
biopolymer biosynthetic process	4	1	1.53	100	0.8595	0.8595
cellular process	46	15	17.6	101	0.888	0.9994
macromolecule biosynthetic process	5	1	1.91	102	0.9152	0.9152
cellular macromolecule biosynthetic proc...	5	1	1.91	103	0.9152	0.9152
regulation of cellular process	5	1	1.91	104	0.9152	0.9152
response to stimulus	10	2	3.83	105	0.9498	0.9498
primary metabolic process	41	12	15.69	106	0.9544	1
nucleobase, nucleoside, nucleotide and n...	7	1	2.68	107	0.9696	0.9696
biosynthetic process	11	1	4.21	108	0.9964	0.9964
cellular biosynthetic process	11	1	4.21	109	0.9964	0.9964
biological_process	115	44	44	110	1	1
peptidoglycan metabolic process	2	0	0.77	111	1	1
M phase	1	0	0.38	112	1	1
gastrulation with mouth forming first	1	0	0.38	113	1	1
carbohydrate metabolic process	6	0	2.3	114	1	1
polysaccharide metabolic process	1	0	0.38	115	1	1
monosaccharide metabolic process	1	0	0.38	116	1	1
fucose metabolic process	1	0	0.38	117	1	1
L-fucose biosynthetic process	1	0	0.38	118	1	1
mannose metabolic process	1	0	0.38	119	1	1
chitin metabolic process	1	0	0.38	120	1	1
chitin catabolic process	1	0	0.38	121	1	1
amino sugar metabolic process	1	0	0.38	122	1	1
glucosamine metabolic process	1	0	0.38	123	1	1
glucosamine catabolic process	1	0	0.38	124	1	1
N-acetylglucosamine metabolic process	1	0	0.38	125	1	1
N-acetylglucosamine catabolic process	1	0	0.38	126	1	1
cellular alcohol metabolic process	1	0	0.38	127	1	1
organic acid metabolic process	6	0	2.3	128	1	1
purine base metabolic process	1	0	0.38	129	1	1
purine nucleotide metabolic process	3	0	1.15	130	1	1
purine nucleotide biosynthetic process	3	0	1.15	131	1	1
IMP biosynthetic process	3	0	1.15	132	1	1
'de novo' IMP biosynthetic process	2	0	0.77	133	1	1
DNA packaging	2	0	0.77	134	1	1
establishment or maintenance of chromati...	2	0	0.77	135	1	1
chromatin assembly or disassembly	2	0	0.77	136	1	1
nucleosome assembly	2	0	0.77	137	1	1
translation	1	0	0.38	138	1	1
protein folding	1	0	0.38	139	1	1
protein modification process	3	0	1.15	140	1	1
protein amino acid phosphorylation	1	0	0.38	141	1	1
protein amino acid glycosylation	1	0	0.38	142	1	1
cellular amino acid and derivative metab...	2	0	0.77	143	1	1
amino acid metabolic process	2	0	0.77	144	1	1
asparagine metabolic process	1	0	0.38	145	1	1
asparagine biosynthetic process	1	0	0.38	146	1	1
glutamine metabolic process	1	0	0.38	147	1	1
glutamine biosynthetic process	1	0	0.38	148	1	1
lipid metabolic process	9	0	3.44	149	1	1

Term	Annotated	Significant	Expected	Rank in classic Fisher	classic Fisher	elimFisher
fatty acid metabolic process	3	0	1.15	150	1	1
fatty acid biosynthetic process	1	0	0.38	151	1	1
acyl-CoA metabolic process	1	0	0.38	152	1	1
membrane lipid metabolic process	2	0	0.77	153	1	1
phospholipid metabolic process	2	0	0.77	154	1	1
sphingolipid metabolic process	2	0	0.77	155	1	1
sphingomyelin metabolic process	2	0	0.77	156	1	1
sphingomyelin catabolic process	2	0	0.77	157	1	1
cellular aromatic compound metabolic pro...	2	0	0.77	158	1	1
coenzyme metabolic process	2	0	0.77	159	1	1
group transfer coenzyme metabolic proces...	1	0	0.38	160	1	1
nucleoside phosphate metabolic process	3	0	1.15	161	1	1
folic acid and derivative metabolic proc...	1	0	0.38	162	1	1
phosphorus metabolic process	2	0	0.77	163	1	1
phosphate metabolic process	2	0	0.77	164	1	1
nitrogen compound metabolic process	2	0	0.77	165	1	1
sodium ion transport	1	0	0.38	166	1	1
phosphate transport	1	0	0.38	167	1	1
anion transport	1	0	0.38	168	1	1
response to stress	4	0	1.53	169	1	1
defense response	2	0	0.77	170	1	1
ER-nuclear signaling pathway	2	0	0.77	171	1	1
response to unfolded protein	2	0	0.77	172	1	1
organelle organization	2	0	0.77	173	1	1
cell wall organization	2	0	0.77	174	1	1
cell cycle	1	0	0.38	175	1	1
chromosome segregation	1	0	0.38	176	1	1
meiosis	1	0	0.38	177	1	1
cell communication	3	0	1.15	178	1	1
cell adhesion	1	0	0.38	179	1	1
cell-matrix adhesion	1	0	0.38	180	1	1
signal transduction	2	0	0.77	181	1	1
intracellular signaling cascade	2	0	0.77	182	1	1
gastrulation	1	0	0.38	183	1	1
lipid biosynthetic process	1	0	0.38	184	1	1
amino acid biosynthetic process	2	0	0.77	185	1	1
catabolic process	5	0	1.91	186	1	1
glutamine family amino acid metabolic pr...	1	0	0.38	187	1	1
aspartate family amino acid metabolic pr...	1	0	0.38	188	1	1
aspartate family amino acid biosynthetic...	1	0	0.38	189	1	1
glutamine family amino acid biosynthetic...	1	0	0.38	190	1	1
glycoprotein metabolic process	1	0	0.38	191	1	1
glycoprotein biosynthetic process	1	0	0.38	192	1	1
coenzyme biosynthetic process	1	0	0.38	193	1	1
nucleobase metabolic process	1	0	0.38	194	1	1
purine base biosynthetic process	1	0	0.38	195	1	1
nucleotide metabolic process	3	0	1.15	196	1	1
nucleoside monophosphate metabolic proce...	3	0	1.15	197	1	1
nucleoside monophosphate biosynthetic pr...	3	0	1.15	198	1	1
purine nucleoside monophosphate metaboli...	3	0	1.15	199	1	1
purine nucleoside monophosphate biosynth...	3	0	1.15	200	1	1
purine ribonucleotide metabolic process	3	0	1.15	201	1	1
purine ribonucleotide biosynthetic proce...	3	0	1.15	202	1	1
ribonucleoside monophosphate biosyntheti...	3	0	1.15	203	1	1
ribonucleoside monophosphate metabolic p...	3	0	1.15	204	1	1
nucleotide biosynthetic process	3	0	1.15	205	1	1
purine ribonucleoside monophosphate meta...	3	0	1.15	206	1	1
purine ribonucleoside monophosphate bios...	3	0	1.15	207	1	1
nucleotide-sugar metabolic process	1	0	0.38	208	1	1
nucleotide-sugar biosynthetic process	1	0	0.38	209	1	1
peptidoglycan catabolic process	2	0	0.77	210	1	1
ribonucleotide metabolic process	3	0	1.15	211	1	1
ribonucleotide biosynthetic process	3	0	1.15	212	1	1
cellular amine metabolic process	2	0	0.77	213	1	1
amine biosynthetic process	2	0	0.77	214	1	1
phospholipid catabolic process	2	0	0.77	215	1	1
folic acid and derivative biosynthetic p...	1	0	0.38	216	1	1
response to biotic stimulus	2	0	0.77	217	1	1
gas transport	1	0	0.38	218	1	1
oxygen transport	1	0	0.38	219	1	1
monovalent inorganic cation transport	1	0	0.38	220	1	1
inorganic anion transport	1	0	0.38	221	1	1
lipid catabolic process	2	0	0.77	222	1	1
cellular component organization	4	0	1.53	223	1	1
carbohydrate biosynthetic process	1	0	0.38	224	1	1
carbohydrate catabolic process	3	0	1.15	225	1	1

Term	Annotated	Significant	Expected	Rank in classic Fisher	classic Fisher	elimFisher
organic acid biosynthetic process	1	0	0.38	226	1	1
phosphorylation	1	0	0.38	227	1	1
protein processing	1	0	0.38	228	1	1
intein-mediated protein splicing	1	0	0.38	229	1	1
cell wall catabolic process	2	0	0.77	230	1	1
molting cycle, protein-based cuticle	1	0	0.38	231	1	1
molting cycle, collagen and cuticulin-ba...	1	0	0.38	232	1	1
hexose metabolic process	1	0	0.38	233	1	1
hexose biosynthetic process	1	0	0.38	234	1	1
GDP-mannose metabolic process	1	0	0.38	235	1	1
carboxylic acid metabolic process	6	0	2.3	236	1	1
cell cycle process	1	0	0.38	237	1	1
cell cycle phase	1	0	0.38	238	1	1
cellular component assembly	2	0	0.77	239	1	1
biological adhesion	1	0	0.38	240	1	1
sphingolipid catabolic process	2	0	0.77	241	1	1
protein splicing	1	0	0.38	242	1	1
endoplasmic reticulum unfolded protein r...	2	0	0.77	243	1	1
chromatin assembly	2	0	0.77	244	1	1
cell-substrate adhesion	1	0	0.38	245	1	1
monocarboxylic acid metabolic process	4	0	1.53	246	1	1
cellular response to stress	2	0	0.77	247	1	1
nucleobase, nucleoside and nucleotide bi...	1	0	0.38	248	1	1
cellular response to unfolded protein	2	0	0.77	249	1	1
cellular macromolecular complex subunit ...	2	0	0.77	250	1	1
cellular macromolecular complex assembly	2	0	0.77	251	1	1
cellular carbohydrate biosynthetic proce...	1	0	0.38	252	1	1
cellular nitrogen compound metabolic pro...	2	0	0.77	253	1	1
nucleobase, nucleoside, nucleotide and n...	1	0	0.38	254	1	1
nucleosome organization	2	0	0.77	255	1	1
response to endoplasmic reticulum stress	2	0	0.77	256	1	1
multicellular organism growth	1	0	0.38	257	1	1
regulation of multicellular organism gro...	1	0	0.38	258	1	1
positive regulation of multicellular org...	1	0	0.38	259	1	1
response to chemical stimulus	2	0	0.77	260	1	1
molting cycle	1	0	0.38	261	1	1
GDP-L-fucose biosynthetic process	1	0	0.38	262	1	1
fucose biosynthetic process	1	0	0.38	263	1	1
L-fucose metabolic process	1	0	0.38	264	1	1
pigment metabolic process	1	0	0.38	265	1	1
biopolymer modification	3	0	1.15	266	1	1
biopolymer glycosylation	1	0	0.38	267	1	1
post-translational protein modification	2	0	0.77	268	1	1
macromolecular complex subunit organizat...	2	0	0.77	269	1	1
cell wall metabolic process	2	0	0.77	270	1	1
cellular lipid catabolic process	2	0	0.77	271	1	1
cellular catabolic process	2	0	0.77	272	1	1
cellular lipid metabolic process	5	0	1.91	273	1	1
cellular carbohydrate metabolic process	1	0	0.38	274	1	1
nitrogen compound biosynthetic process	2	0	0.77	275	1	1
meiotic chromosome segregation	1	0	0.38	276	1	1
external encapsulating structure organiz...	2	0	0.77	277	1	1
IMP metabolic process	3	0	1.15	278	1	1
nucleobase biosynthetic process	1	0	0.38	279	1	1
pigment biosynthetic process	1	0	0.38	280	1	1
alcohol biosynthetic process	1	0	0.38	281	1	1
amino sugar catabolic process	1	0	0.38	282	1	1
monosaccharide biosynthetic process	1	0	0.38	283	1	1
GDP-L-fucose metabolic process	1	0	0.38	284	1	1
carboxylic acid biosynthetic process	1	0	0.38	285	1	1
membrane lipid catabolic process	2	0	0.77	286	1	1
heterocycle metabolic process	2	0	0.77	287	1	1
embryonic morphogenesis	1	0	0.38	288	1	1
cofactor metabolic process	2	0	0.77	289	1	1
cofactor biosynthetic process	1	0	0.38	290	1	1
regulation of multicellular organismal p...	1	0	0.38	291	1	1
positive regulation of multicellular org...	1	0	0.38	292	1	1
chromosome organization	2	0	0.77	293	1	1
meiotic cell cycle	1	0	0.38	294	1	1
M phase of meiotic cell cycle	1	0	0.38	295	1	1
cellular response to stimulus	2	0	0.77	296	1	1
response to protein stimulus	2	0	0.77	297	1	1
nucleobase, nucleoside and nucleotide me...	3	0	1.15	298	1	1
oxidation reduction	1	0	0.38	299	1	1
macromolecular complex assembly	2	0	0.77	300	1	1
protein-DNA complex assembly	2	0	0.77	301	1	1
glycosylation	1	0	0.38	302	1	1
