# Supplementary material for: Genome-Wide Analysis Reveals Novel Genes Essential for Heme Homeostasis in Caenorhabditis elegans
Source: PLoS Genet. 2010 Jul 29;6(7):e1001044. doi: 10.1371/journal.pgen.1001044 (PMC2912396; doi:10.1371/journal.pgen.1001044)
Supplement: Table S7 — Gene Ontology analysis of heme-responsive genes downregulated at 4 µM heme. Each GO ID is assigned a unique function or association. Both are listed here, even if the GO ID was not used in the GO analysis figure. Green shading indicates that term was included in the corresponding GO enrichment figures. (0.06 MB PDF) [file pgen.1001044.s009.pdf]

Term	Annotated	Significant	Expected	Rank in classic Fisher	classic Fisher	elimFisher
response to stimulus	10	7	2.43	1	0.0018	0.114
response to stress	4	4	0.97	2	0.003	0.003
catabolic process	5	4	1.22	3	0.0122	0.0122
cell communication	3	3	0.73	4	0.0133	0.0133
lipid metabolic process	9	5	2.19	5	0.0375	0.0375
cellular component organization	4	3	0.97	6	0.0442	0.0442
peptidoglycan metabolic process	2	2	0.49	7	0.0577	0.0577
membrane lipid metabolic process	2	2	0.49	8	0.0577	0.0577
phospholipid metabolic process	2	2	0.49	9	0.0577	0.0577
sphingolipid metabolic process	2	2	0.49	10	0.0577	0.0577
sphingomyelin metabolic process	2	2	0.49	11	0.0577	0.0577
sphingomyelin catabolic process	2	2	0.49	12	0.0577	0.0577
defense response	2	2	0.49	13	0.0577	0.0577
ER-nuclear signaling pathway	2	2	0.49	14	0.0577	0.0577
response to unfolded protein	2	2	0.49	15	0.0577	0.0577
cell wall organization	2	2	0.49	16	0.0577	0.0577
signal transduction	2	2	0.49	17	0.0577	0.0577
intracellular signaling cascade	2	2	0.49	18	0.0577	0.0577
peptidoglycan catabolic process	2	2	0.49	19	0.0577	0.0577
phospholipid catabolic process	2	2	0.49	20	0.0577	0.0577
response to biotic stimulus	2	2	0.49	21	0.0577	0.0577
lipid catabolic process	2	2	0.49	22	0.0577	0.0577
cell wall catabolic process	2	2	0.49	23	0.0577	0.0577
sphingolipid catabolic process	2	2	0.49	24	0.0577	0.0577
endoplasmic reticulum unfolded protein r...	2	2	0.49	25	0.0577	0.0577
cellular response to stress	2	2	0.49	26	0.0577	0.0577
cellular response to unfolded protein	2	2	0.49	27	0.0577	0.0577
response to endoplasmic reticulum stress	2	2	0.49	28	0.0577	0.0577
response to chemical stimulus	2	2	0.49	29	0.0577	0.0577
cell wall metabolic process	2	2	0.49	30	0.0577	0.0577
cellular lipid catabolic process	2	2	0.49	31	0.0577	0.0577
cellular catabolic process	2	2	0.49	32	0.0577	0.0577
external encapsulating structure organiz...	2	2	0.49	33	0.0577	0.0577
membrane lipid catabolic process	2	2	0.49	34	0.0577	0.0577
cellular response to stimulus	2	2	0.49	35	0.0577	0.0577
response to protein stimulus	2	2	0.49	36	0.0577	0.0577
reproductive process	7	4	1.7	37	0.0587	0.0587
cellular lipid metabolic process	5	3	1.22	38	0.0921	0.0921
carbohydrate catabolic process	3	2	0.73	39	0.1465	0.1465
carbohydrate metabolic process	6	3	1.46	40	0.1538	0.1538
behavior	6	3	1.46	41	0.1538	0.1538
oviposition	6	3	1.46	42	0.1538	0.1538
reproductive behavior	6	3	1.46	43	0.1538	0.1538
multicellular organism reproduction	6	3	1.46	44	0.1538	0.1538
reproductive behavior in a multicellular...	6	3	1.46	45	0.1538	0.1538
reproductive process in a multicellular ...	6	3	1.46	46	0.1538	0.1538
reproduction	13	5	3.17	47	0.1775	0.1775
anatomical structure development	7	3	1.7	48	0.2251	0.2251
gastrulation with mouth forming first	1	1	0.24	49	0.2435	0.2435
monosaccharide metabolic process	1	1	0.24	50	0.2435	0.2435
fucose metabolic process	1	1	0.24	51	0.2435	0.2435
L-fucose biosynthetic process	1	1	0.24	52	0.2435	0.2435
mannose metabolic process	1	1	0.24	53	0.2435	0.2435
cellular alcohol metabolic process	1	1	0.24	54	0.2435	0.2435
protein folding	1	1	0.24	55	0.2435	0.2435
fatty acid biosynthetic process	1	1	0.24	56	0.2435	0.2435
phosphate transport	1	1	0.24	57	0.2435	0.2435
anion transport	1	1	0.24	58	0.2435	0.2435
gastrulation	1	1	0.24	59	0.2435	0.2435
lipid biosynthetic process	1	1	0.24	60	0.2435	0.2435
nucleotide-sugar metabolic process	1	1	0.24	61	0.2435	0.2435
nucleotide-sugar biosynthetic process	1	1	0.24	62	0.2435	0.2435
inorganic anion transport	1	1	0.24	63	0.2435	0.2435
carbohydrate biosynthetic process	1	1	0.24	64	0.2435	0.2435
organic acid biosynthetic process	1	1	0.24	65	0.2435	0.2435
protein processing	1	1	0.24	66	0.2435	0.2435
intein-mediated protein splicing	1	1	0.24	67	0.2435	0.2435
molting cycle, protein-based cuticle	1	1	0.24	68	0.2435	0.2435
molting cycle, collagen and cuticulin-ba...	1	1	0.24	69	0.2435	0.2435
hexose metabolic process	1	1	0.24	70	0.2435	0.2435
hexose biosynthetic process	1	1	0.24	71	0.2435	0.2435
GDP-mannose metabolic process	1	1	0.24	72	0.2435	0.2435
protein splicing	1	1	0.24	73	0.2435	0.2435
cellular carbohydrate biosynthetic proce...	1	1	0.24	74	0.2435	0.2435

Term	Annotated	Significant	Expected	Rank in classic Fisher	classic Fisher	elimFisher
multicellular organism growth	1	1	0.24	75	0.2435	0.2435
regulation of multicellular organism gro...	1	1	0.24	76	0.2435	0.2435
positive regulation of multicellular org...	1	1	0.24	77	0.2435	0.2435
molting cycle	1	1	0.24	78	0.2435	0.2435
GDP-L-fucose biosynthetic process	1	1	0.24	79	0.2435	0.2435
fucose biosynthetic process	1	1	0.24	80	0.2435	0.2435
L-fucose metabolic process	1	1	0.24	81	0.2435	0.2435
cellular carbohydrate metabolic process	1	1	0.24	82	0.2435	0.2435
alcohol biosynthetic process	1	1	0.24	83	0.2435	0.2435
monosaccharide biosynthetic process	1	1	0.24	84	0.2435	0.2435
GDP-L-fucose metabolic process	1	1	0.24	85	0.2435	0.2435
carboxylic acid biosynthetic process	1	1	0.24	86	0.2435	0.2435
embryonic morphogenesis	1	1	0.24	87	0.2435	0.2435
regulation of multicellular organismal p...	1	1	0.24	88	0.2435	0.2435
positive regulation of multicellular org...	1	1	0.24	89	0.2435	0.2435
oxidation reduction	1	1	0.24	90	0.2435	0.2435
primary metabolic process	41	12	9.98	91	0.244	0.244
cellular process	46	13	11.2	92	0.2806	0.2806
anatomical structure morphogenesis	5	2	1.22	93	0.3532	0.3532
regulation of cellular process	5	2	1.22	94	0.3532	0.3532
reproductive developmental process	2	1	0.49	95	0.4293	0.4293
DNA packaging	2	1	0.49	96	0.4293	0.4293
establishment or maintenance of chromati...	2	1	0.49	97	0.4293	0.4293
chromatin assembly or disassembly	2	1	0.49	98	0.4293	0.4293
nucleosome assembly	2	1	0.49	99	0.4293	0.4293
organelle organization	2	1	0.49	100	0.4293	0.4293
sex differentiation	2	1	0.49	101	0.4293	0.4293
cellular component assembly	2	1	0.49	102	0.4293	0.4293
chromatin assembly	2	1	0.49	103	0.4293	0.4293
cellular macromolecular complex subunit ...	2	1	0.49	104	0.4293	0.4293
cellular macromolecular complex assembly	2	1	0.49	105	0.4293	0.4293
nucleosome organization	2	1	0.49	106	0.4293	0.4293
hermaphrodite genitalia development	2	1	0.49	107	0.4293	0.4293
post-translational protein modification	2	1	0.49	108	0.4293	0.4293
macromolecular complex subunit organizat...	2	1	0.49	109	0.4293	0.4293
organ development	2	1	0.49	110	0.4293	0.4293
system development	2	1	0.49	111	0.4293	0.4293
genitalia development	2	1	0.49	112	0.4293	0.4293
chromosome organization	2	1	0.49	113	0.4293	0.4293
macromolecular complex assembly	2	1	0.49	114	0.4293	0.4293
protein-DNA complex assembly	2	1	0.49	115	0.4293	0.4293
multicellular organismal process	39	10	9.5	116	0.4941	0.4941
growth	23	6	5.6	117	0.5101	0.5101
aging	11	3	2.68	118	0.5307	0.5307
determination of adult life span	11	3	2.68	119	0.5307	0.5307
multicellular organismal aging	11	3	2.68	120	0.5307	0.5307
protein modification process	3	1	0.73	121	0.5707	0.5707
fatty acid metabolic process	3	1	0.73	122	0.5707	0.5707
ion transport	3	1	0.73	123	0.5707	0.5707
body morphogenesis	3	1	0.73	124	0.5707	0.5707
biopolymer modification	3	1	0.73	125	0.5707	0.5707
regulation of growth	20	5	4.87	126	0.5711	0.5711
positive regulation of growth	20	5	4.87	127	0.5711	0.5711
positive regulation of biological proces...	20	5	4.87	128	0.5711	0.5711
monocarboxylic acid metabolic process	4	1	0.97	129	0.678	0.678
multicellular organismal development	35	8	8.52	130	0.6804	0.6804
cellular metabolic process	35	8	8.52	131	0.6804	0.6804
regulation of biological process	23	5	5.6	132	0.718	0.718
protein metabolic process	19	4	4.63	133	0.7378	0.7378
regulation of growth rate	19	4	4.63	134	0.7378	0.7378
positive regulation of growth rate	19	4	4.63	135	0.7378	0.7378
cellular protein metabolic process	19	4	4.63	136	0.7378	0.7378
developmental process	37	8	9.01	137	0.7559	0.7559
biological regulation	24	5	5.84	138	0.759	0.759
nematode larval development	5	1	1.22	139	0.7592	0.7592
larval development	5	1	1.22	140	0.7592	0.7592
post-embryonic development	5	1	1.22	141	0.7592	0.7592
proteolysis	15	3	3.65	142	0.7645	0.7645
biosynthetic process	11	2	2.68	143	0.8032	0.8032
locomotion	11	2	2.68	144	0.8032	0.8032
cellular biosynthetic process	11	2	2.68	145	0.8032	0.8032
organic acid metabolic process	6	1	1.46	146	0.8205	0.8205
carboxylic acid metabolic process	6	1	1.46	147	0.8205	0.8205
metabolic process	56	12	13.63	148	0.8232	0.8232

Term	Annotated	Significant	Expected	Rank in classic Fisher	classic Fisher	elim Fisher
transport	17	3	4.14	149	0.8423	0.8423
localization	17	3	4.14	150	0.8423	0.8423
establishment of localization	17	3	4.14	151	0.8423	0.8423
cellular biopolymer metabolic process	22	4	5.36	152	0.8479	0.8479
cellular macromolecule metabolic process	22	4	5.36	153	0.8479	0.8479
nucleobase, nucleoside, nucleotide and n...	7	1	1.7	154	0.8666	0.8666
macromolecule metabolic process	23	4	5.6	155	0.8753	0.8753
biopolymer metabolic process	23	4	5.6	156	0.8753	0.8753
embryonic development	18	2	4.38	157	0.9668	0.9668
embryonic development ending in birth or...	17	1	4.14	158	0.9943	0.9943
biological_process	115	28	28	159	1	1
transition metal ion transport	1	0	0.24	160	1	1
M phase	1	0	0.24	161	1	1
cell fate specification	1	0	0.24	162	1	1
morphogenesis of an epithelium	1	0	0.24	163	1	1
polysaccharide metabolic process	1	0	0.24	164	1	1
chitin metabolic process	1	0	0.24	165	1	1
chitin catabolic process	1	0	0.24	166	1	1
amino sugar metabolic process	1	0	0.24	167	1	1
glucosamine metabolic process	1	0	0.24	168	1	1
glucosamine catabolic process	1	0	0.24	169	1	1
N-acetylglucosamine metabolic process	1	0	0.24	170	1	1
N-acetylglucosamine catabolic process	1	0	0.24	171	1	1
purine base metabolic process	1	0	0.24	172	1	1
purine nucleotide metabolic process	3	0	0.73	173	1	1
purine nucleotide biosynthetic process	3	0	0.73	174	1	1
IMP biosynthetic process	3	0	0.73	175	1	1
'de novo' IMP biosynthetic process	2	0	0.49	176	1	1
transcription	3	0	0.73	177	1	1
transcription, DNA-dependent	3	0	0.73	178	1	1
regulation of transcription, DNA-depende...	3	0	0.73	179	1	1
translation	1	0	0.24	180	1	1
protein amino acid phosphorylation	1	0	0.24	181	1	1
protein amino acid glycosylation	1	0	0.24	182	1	1
cellular amino acid and derivative metab...	2	0	0.49	183	1	1
amino acid metabolic process	2	0	0.49	184	1	1
asparagine metabolic process	1	0	0.24	185	1	1
asparagine biosynthetic process	1	0	0.24	186	1	1
glutamine metabolic process	1	0	0.24	187	1	1
glutamine biosynthetic process	1	0	0.24	188	1	1
acyl-CoA metabolic process	1	0	0.24	189	1	1
cellular aromatic compound metabolic pro...	2	0	0.49	190	1	1
coenzyme metabolic process	2	0	0.49	191	1	1
group transfer coenzyme metabolic proces...	1	0	0.24	192	1	1
nucleoside phosphate metabolic process	3	0	0.73	193	1	1
folic acid and derivative metabolic proc...	1	0	0.24	194	1	1
phosphorus metabolic process	2	0	0.49	195	1	1
phosphate metabolic process	2	0	0.49	196	1	1
nitrogen compound metabolic process	2	0	0.49	197	1	1
cation transport	2	0	0.49	198	1	1
sodium ion transport	1	0	0.24	199	1	1
iron ion transport	1	0	0.24	200	1	1
lipid transport	6	0	1.46	201	1	1
cellular ion homeostasis	1	0	0.24	202	1	1
cellular iron ion homeostasis	1	0	0.24	203	1	1
cell cycle	1	0	0.24	204	1	1
chromosome segregation	1	0	0.24	205	1	1
meiosis	1	0	0.24	206	1	1
cell adhesion	1	0	0.24	207	1	1
cell-matrix adhesion	1	0	0.24	208	1	1
pattern specification process	1	0	0.24	209	1	1
protein-based cuticle development	1	0	0.24	210	1	1
amino acid biosynthetic process	2	0	0.49	211	1	1
macromolecule biosynthetic process	5	0	1.22	212	1	1
glutamine family amino acid metabolic pr...	1	0	0.24	213	1	1
aspartate family amino acid metabolic pr...	1	0	0.24	214	1	1
aspartate family amino acid biosynthetic...	1	0	0.24	215	1	1
glutamine family amino acid biosynthetic...	1	0	0.24	216	1	1
glycoprotein metabolic process	1	0	0.24	217	1	1
glycoprotein biosynthetic process	1	0	0.24	218	1	1
coenzyme biosynthetic process	1	0	0.24	219	1	1
nucleobase metabolic process	1	0	0.24	220	1	1
purine base biosynthetic process	1	0	0.24	221	1	1
nucleotide metabolic process	3	0	0.73	222	1	1
nucleoside monophosphate metabolic proce...	3	0	0.73	223	1	1
nucleoside monophosphate biosynthetic pr...	3	0	0.73	224	1	1
purine nucleoside monophosphate metaboli...	3	0	0.73	225	1	1

Term	Annotated	Significant	Expected	Rank in classic Fisher	classic Fisher	elim Fisher
purine nucleoside monophosphate biosynth...	3	0	0.73	226	1	1
purine ribonucleotide metabolic process	3	0	0.73	227	1	1
purine ribonucleotide biosynthetic proce...	3	0	0.73	228	1	1
ribonucleoside monophosphate biosyntheti...	3	0	0.73	229	1	1
ribonucleoside monophosphate metabolic p...	3	0	0.73	230	1	1
nucleotide biosynthetic process	3	0	0.73	231	1	1
purine ribonucleoside monophosphate meta...	3	0	0.73	232	1	1
purine ribonucleoside monophosphate bios...	3	0	0.73	233	1	1
ribonucleotide metabolic process	3	0	0.73	234	1	1
ribonucleotide biosynthetic process	3	0	0.73	235	1	1
cellular amine metabolic process	2	0	0.49	236	1	1
amine biosynthetic process	2	0	0.49	237	1	1
folic acid and derivative biosynthetic p...	1	0	0.24	238	1	1
embryonic pattern specification	1	0	0.24	239	1	1
regulation of biosynthetic process	3	0	0.73	240	1	1
gene expression	4	0	0.97	241	1	1
regulation of gene expression	3	0	0.73	242	1	1
regulation of macromolecule biosynthetic...	3	0	0.73	243	1	1
gas transport	1	0	0.24	244	1	1
oxygen transport	1	0	0.24	245	1	1
monovalent inorganic cation transport	1	0	0.24	246	1	1
di-, tri-valent inorganic cation transpo...	1	0	0.24	247	1	1
RNA metabolic process	3	0	0.73	248	1	1
phosphorylation	1	0	0.24	249	1	1
regulation of nucleobase, nucleoside, nu...	3	0	0.73	250	1	1
regulation of metabolic process	3	0	0.73	251	1	1
cellular homeostasis	1	0	0.24	252	1	1
cell cycle process	1	0	0.24	253	1	1
cell cycle phase	1	0	0.24	254	1	1
biological adhesion	1	0	0.24	255	1	1
metal ion transport	2	0	0.49	256	1	1
cellular cation homeostasis	1	0	0.24	257	1	1
cellular di-, tri-valent inorganic catio...	1	0	0.24	258	1	1
cell differentiation	1	0	0.24	259	1	1
regulation of cellular metabolic process	3	0	0.73	260	1	1
regulation of cellular biosynthetic proc...	3	0	0.73	261	1	1
cell-substrate adhesion	1	0	0.24	262	1	1
RNA biosynthetic process	3	0	0.73	263	1	1
nucleobase, nucleoside and nucleotide bi...	1	0	0.24	264	1	1
cellular nitrogen compound metabolic pro...	2	0	0.49	265	1	1
cellular macromolecule biosynthetic proc...	5	0	1.22	266	1	1
nucleobase, nucleoside, nucleotide and n...	1	0	0.24	267	1	1
cellular biopolymer biosynthetic process	4	0	0.97	268	1	1
collagen and cuticulin-based cuticle dev...	1	0	0.24	269	1	1
regulation of locomotion	1	0	0.24	270	1	1
positive regulation of locomotion	1	0	0.24	271	1	1
cuticle development	1	0	0.24	272	1	1
pigment metabolic process	1	0	0.24	273	1	1
homeostatic process	1	0	0.24	274	1	1
biopolymer biosynthetic process	4	0	0.97	275	1	1
biopolymer glycosylation	1	0	0.24	276	1	1
nitrogen compound biosynthetic process	2	0	0.49	277	1	1
meiotic chromosome segregation	1	0	0.24	278	1	1
cell fate commitment	1	0	0.24	279	1	1
regulation of transcription	3	0	0.73	280	1	1
IMP metabolic process	3	0	0.73	281	1	1
nucleobase biosynthetic process	1	0	0.24	282	1	1
pigment biosynthetic process	1	0	0.24	283	1	1
amino sugar catabolic process	1	0	0.24	284	1	1
heterocycle metabolic process	2	0	0.49	285	1	1
cellular developmental process	1	0	0.24	286	1	1
chemical homeostasis	1	0	0.24	287	1	1
ion homeostasis	1	0	0.24	288	1	1
cofactor metabolic process	2	0	0.49	289	1	1
cofactor biosynthetic process	1	0	0.24	290	1	1
regulation of RNA metabolic process	3	0	0.73	291	1	1
meiotic cell cycle	1	0	0.24	292	1	1
M phase of meiotic cell cycle	1	0	0.24	293	1	1
di-, tri-valent inorganic cation homeost...	1	0	0.24	294	1	1
iron ion homeostasis	1	0	0.24	295	1	1
cation homeostasis	1	0	0.24	296	1	1
cellular chemical homeostasis	1	0	0.24	297	1	1
transmembrane transport	1	0	0.24	298	1	1
nucleobase, nucleoside and nucleotide me...	3	0	0.73	299	1	1
regulation of macromolecule metabolic pr...	3	0	0.73	300	1	1
regulation of biological quality	1	0	0.24	301	1	1
glycosylation	1	0	0.24	302	1	1
