# Supplementary material for: Genome-Wide Analysis Reveals Novel Genes Essential for Heme Homeostasis in Caenorhabditis elegans
Source: PLoS Genet. 2010 Jul 29;6(7):e1001044. doi: 10.1371/journal.pgen.1001044 (PMC2912396; doi:10.1371/journal.pgen.1001044)
Supplement: Table S8 — Gene Ontology analysis of heme-responsive genes upregulated at 500 µM heme. Each GO ID is assigned a unique function or association. Both are listed here, even if the GO ID was not used in the GO analysis figure. Green shading indicates that term was included in the corresponding GO enrichment figures. (0.09 MB PDF) [file pgen.1001044.s010.pdf]

Term	Annotated	Significant	Expected	Rank in classic Fisher	classic Fisher	elim Fisher
fatty acid metabolic process	3	2	0.91	1	0.22	0.22
organic acid metabolic process	6	3	1.83	2	0.26	0.26
carboxylic acid metabolic process	6	3	1.83	3	0.26	0.26
lipid metabolic process	9	4	2.74	4	0.27	0.27
metabolic process	56	19	17.04	5	0.28	0.28
M phase	1	1	0.3	6	0.3	0.3
polysaccharide metabolic process	1	1	0.3	7	0.3	0.3
chitin metabolic process	1	1	0.3	8	0.3	0.3
chitin catabolic process	1	1	0.3	9	0.3	0.3
amino sugar metabolic process	1	1	0.3	10	0.3	0.3
glucosamine metabolic process	1	1	0.3	11	0.3	0.3
glucosamine catabolic process	1	1	0.3	12	0.3	0.3
N-acetylglucosamine metabolic process	1	1	0.3	13	0.3	0.3
N-acetylglucosamine catabolic process	1	1	0.3	14	0.3	0.3
translation	1	1	0.3	15	0.3	0.3
glutamine metabolic process	1	1	0.3	16	0.3	0.3
glutamine biosynthetic process	1	1	0.3	17	0.3	0.3
acyl-CoA metabolic process	1	1	0.3	18	0.3	0.3
cell cycle	1	1	0.3	19	0.3	0.3
chromosome segregation	1	1	0.3	20	0.3	0.3
meiosis	1	1	0.3	21	0.3	0.3
glutamine family amino acid metabolic pr...	1	1	0.3	22	0.3	0.3
glutamine family amino acid biosynthetic...	1	1	0.3	23	0.3	0.3
gas transport	1	1	0.3	24	0.3	0.3
oxygen transport	1	1	0.3	25	0.3	0.3
cell cycle process	1	1	0.3	26	0.3	0.3
cell cycle phase	1	1	0.3	27	0.3	0.3
meiotic chromosome segregation	1	1	0.3	28	0.3	0.3
amino sugar catabolic process	1	1	0.3	29	0.3	0.3
meiotic cell cycle	1	1	0.3	30	0.3	0.3
M phase of meiotic cell cycle	1	1	0.3	31	0.3	0.3
transmembrane transport	1	1	0.3	32	0.3	0.3
gene expression	4	2	1.22	33	0.36	0.36
monocarboxylic acid metabolic process	4	2	1.22	34	0.36	0.36
cellular biopolymer biosynthetic process	4	2	1.22	35	0.36	0.36
biopolymer biosynthetic process	4	2	1.22	36	0.36	0.36
macromolecule metabolic process	23	8	7	37	0.39	0.39
biopolymer metabolic process	23	8	7	38	0.39	0.39
macromolecule biosynthetic process	5	2	1.52	39	0.48	0.48
cellular macromolecule biosynthetic proc...	5	2	1.52	40	0.48	0.48
cellular lipid metabolic process	5	2	1.52	41	0.48	0.48
primary metabolic process	41	13	12.48	42	0.49	0.49
proteolysis	15	5	4.57	43	0.5	0.5
DNA packaging	2	1	0.61	44	0.52	0.52
establishment or maintenance of chromati...	2	1	0.61	45	0.52	0.52
chromatin assembly or disassembly	2	1	0.61	46	0.52	0.52
nucleosome assembly	2	1	0.61	47	0.52	0.52
cellular amino acid and derivative metab...	2	1	0.61	48	0.52	0.52
amino acid metabolic process	2	1	0.61	49	0.52	0.52
coenzyme metabolic process	2	1	0.61	50	0.52	0.52
nitrogen compound metabolic process	2	1	0.61	51	0.52	0.52
defense response	2	1	0.61	52	0.52	0.52
organelle organization	2	1	0.61	53	0.52	0.52
amino acid biosynthetic process	2	1	0.61	54	0.52	0.52
cellular amine metabolic process	2	1	0.61	55	0.52	0.52
amine biosynthetic process	2	1	0.61	56	0.52	0.52
cellular component assembly	2	1	0.61	57	0.52	0.52
chromatin assembly	2	1	0.61	58	0.52	0.52
cellular macromolecular complex subunit ...	2	1	0.61	59	0.52	0.52
cellular macromolecular complex assembly	2	1	0.61	60	0.52	0.52
cellular nitrogen compound metabolic pro...	2	1	0.61	61	0.52	0.52
nucleosome organization	2	1	0.61	62	0.52	0.52
macromolecular complex subunit organizat...	2	1	0.61	63	0.52	0.52
nitrogen compound biosynthetic process	2	1	0.61	64	0.52	0.52
cofactor metabolic process	2	1	0.61	65	0.52	0.52
chromosome organization	2	1	0.61	66	0.52	0.52
macromolecular complex assembly	2	1	0.61	67	0.52	0.52
protein-DNA complex assembly	2	1	0.61	68	0.52	0.52
cellular biopolymer metabolic process	22	7	6.7	69	0.53	0.53
cellular macromolecule metabolic process	22	7	6.7	70	0.53	0.53
protein metabolic process	19	6	5.78	71	0.55	0.55
cellular protein metabolic process	19	6	5.78	72	0.55	0.55
transport	17	5	5.17	73	0.64	0.64
localization	17	5	5.17	74	0.64	0.64
establishment of localization	17	5	5.17	75	0.64	0.64

Table 8

Term	Annotated	Significant	Expected	Rank in classic Fisher	classic Fisher	elim Fisher
transcription	3	1	0.91	76	0.67	0.67
transcription, DNA-dependent	3	1	0.91	77	0.67	0.67
regulation of transcription, DNA-depende...	3	1	0.91	78	0.67	0.67
regulation of biosynthetic process	3	1	0.91	79	0.67	0.67
regulation of gene expression	3	1	0.91	80	0.67	0.67
regulation of macromolecule biosynthetic...	3	1	0.91	81	0.67	0.67
carbohydrate catabolic process	3	1	0.91	82	0.67	0.67
RNA metabolic process	3	1	0.91	83	0.67	0.67
regulation of nucleobase, nucleoside, nu...	3	1	0.91	84	0.67	0.67
regulation of metabolic process	3	1	0.91	85	0.67	0.67
regulation of cellular metabolic process	3	1	0.91	86	0.67	0.67
regulation of cellular biosynthetic proc...	3	1	0.91	87	0.67	0.67
RNA biosynthetic process	3	1	0.91	88	0.67	0.67
regulation of transcription	3	1	0.91	89	0.67	0.67
regulation of RNA metabolic process	3	1	0.91	90	0.67	0.67
regulation of macromolecule metabolic pr...	3	1	0.91	91	0.67	0.67
cellular metabolic process	35	10	10.65	92	0.69	0.69
biosynthetic process	11	3	3.35	93	0.71	0.71
locomotion	11	3	3.35	94	0.71	0.71
cellular biosynthetic process	11	3	3.35	95	0.71	0.71
cellular process	46	13	14	96	0.73	0.73
response to stress	4	1	1.22	97	0.77	0.77
cellular component organization	4	1	1.22	98	0.77	0.77
embryonic development ending in birth or...	17	4	5.17	99	0.83	0.83
nematode larval development	5	1	1.52	100	0.84	0.84
larval development	5	1	1.52	101	0.84	0.84
catabolic process	5	1	1.52	102	0.84	0.84
post-embryonic development	5	1	1.52	103	0.84	0.84
regulation of cellular process	5	1	1.52	104	0.84	0.84
embryonic development	18	4	5.48	105	0.87	0.87
response to stimulus	10	2	3.04	106	0.87	0.87
carbohydrate metabolic process	6	1	1.83	107	0.89	0.89
behavior	6	1	1.83	108	0.89	0.89
oviposition	6	1	1.83	109	0.89	0.89
reproductive behavior	6	1	1.83	110	0.89	0.89
multicellular organism reproduction	6	1	1.83	111	0.89	0.89
reproductive behavior in a multicellular...	6	1	1.83	112	0.89	0.89
reproductive process in a multicellular ...	6	1	1.83	113	0.89	0.89
aging	11	2	3.35	114	0.9	0.9
determination of adult life span	11	2	3.35	115	0.9	0.9
multicellular organismal aging	11	2	3.35	116	0.9	0.9
nucleobase, nucleoside, nucleotide and n...	7	1	2.13	117	0.93	0.93
reproductive process	7	1	2.13	118	0.93	0.93
reproduction	13	2	3.96	119	0.95	0.95
multicellular organismal development	35	7	10.65	120	0.97	0.97
multicellular organismal process	39	8	11.87	121	0.97	0.97
developmental process	37	7	11.26	122	0.98	0.98
regulation of biological process	23	3	7	123	0.99	0.99
regulation of growth rate	19	2	5.78	124	0.99	0.99
positive regulation of growth rate	19	2	5.78	125	0.99	0.99
biological regulation	24	3	7.3	126	0.99	0.99
regulation of growth	20	2	6.09	127	1	1
positive regulation of growth	20	2	6.09	128	1	1
positive regulation of biological proces...	20	2	6.09	129	1	1
growth	23	2	7	130	1	1
biological_process	115	35	35	131	1	1
transition metal ion transport	1	0	0.3	132	1	1
peptidoglycan metabolic process	2	0	0.61	133	1	1
gastrulation with mouth forming first	1	0	0.3	134	1	1
cell fate specification	1	0	0.3	135	1	1
morphogenesis of an epithelium	1	0	0.3	136	1	1
reproductive developmental process	2	0	0.61	137	1	1
monosaccharide metabolic process	1	0	0.3	138	1	1
fucose metabolic process	1	0	0.3	139	1	1
L-fucose biosynthetic process	1	0	0.3	140	1	1
mannose metabolic process	1	0	0.3	141	1	1
cellular alcohol metabolic process	1	0	0.3	142	1	1
purine base metabolic process	1	0	0.3	143	1	1
purine nucleotide metabolic process	3	0	0.91	144	1	1
purine nucleotide biosynthetic process	3	0	0.91	145	1	1
IMP biosynthetic process	3	0	0.91	146	1	1
'de novo' IMP biosynthetic process	2	0	0.61	147	1	1
protein folding	1	0	0.3	148	1	1
protein modification process	3	0	0.91	149	1	1
protein amino acid phosphorylation	1	0	0.3	150	1	1

Table 8 (contd)

Term	Annotated	Significant	Expected	Rank in classic Fisher	classic Fisher	elim Fisher
protein amino acid glycosylation	1	0	0.3	151	1	1
asparagine metabolic process	1	0	0.3	152	1	1
asparagine biosynthetic process	1	0	0.3	153	1	1
fatty acid biosynthetic process	1	0	0.3	154	1	1
membrane lipid metabolic process	2	0	0.61	155	1	1
phospholipid metabolic process	2	0	0.61	156	1	1
sphingolipid metabolic process	2	0	0.61	157	1	1
sphingomyelin metabolic process	2	0	0.61	158	1	1
sphingomyelin catabolic process	2	0	0.61	159	1	1
cellular aromatic compound metabolic pro...	2	0	0.61	160	1	1
group transfer coenzyme metabolic proces...	1	0	0.3	161	1	1
nucleoside phosphate metabolic process	3	0	0.91	162	1	1
folic acid and derivative metabolic proc...	1	0	0.3	163	1	1
phosphorus metabolic process	2	0	0.61	164	1	1
phosphate metabolic process	2	0	0.61	165	1	1
ion transport	3	0	0.91	166	1	1
cation transport	2	0	0.61	167	1	1
sodium ion transport	1	0	0.3	168	1	1
phosphate transport	1	0	0.3	169	1	1
anion transport	1	0	0.3	170	1	1
iron ion transport	1	0	0.3	171	1	1
lipid transport	6	0	1.83	172	1	1
cellular ion homeostasis	1	0	0.3	173	1	1
cellular iron ion homeostasis	1	0	0.3	174	1	1
ER-nuclear signaling pathway	2	0	0.61	175	1	1
response to unfolded protein	2	0	0.61	176	1	1
cell wall organization	2	0	0.61	177	1	1
cell communication	3	0	0.91	178	1	1
cell adhesion	1	0	0.3	179	1	1
cell-matrix adhesion	1	0	0.3	180	1	1
signal transduction	2	0	0.61	181	1	1
intracellular signaling cascade	2	0	0.61	182	1	1
gastrulation	1	0	0.3	183	1	1
pattern specification process	1	0	0.3	184	1	1
sex differentiation	2	0	0.61	185	1	1
protein-based cuticle development	1	0	0.3	186	1	1
lipid biosynthetic process	1	0	0.3	187	1	1
aspartate family amino acid metabolic pr...	1	0	0.3	188	1	1
aspartate family amino acid biosynthetic...	1	0	0.3	189	1	1
glycoprotein metabolic process	1	0	0.3	190	1	1
glycoprotein biosynthetic process	1	0	0.3	191	1	1
coenzyme biosynthetic process	1	0	0.3	192	1	1
nucleobase metabolic process	1	0	0.3	193	1	1
purine base biosynthetic process	1	0	0.3	194	1	1
nucleotide metabolic process	3	0	0.91	195	1	1
nucleoside monophosphate metabolic proce...	3	0	0.91	196	1	1
nucleoside monophosphate biosynthetic pr...	3	0	0.91	197	1	1
purine nucleoside monophosphate metaboli...	3	0	0.91	198	1	1
purine nucleoside monophosphate biosynth...	3	0	0.91	199	1	1
purine ribonucleotide metabolic process	3	0	0.91	200	1	1
purine ribonucleotide biosynthetic proce...	3	0	0.91	201	1	1
ribonucleoside monophosphate biosyntheti...	3	0	0.91	202	1	1
ribonucleoside monophosphate metabolic p...	3	0	0.91	203	1	1
nucleotide biosynthetic process	3	0	0.91	204	1	1
purine ribonucleoside monophosphate meta...	3	0	0.91	205	1	1
purine ribonucleoside monophosphate bios...	3	0	0.91	206	1	1
nucleotide-sugar metabolic process	1	0	0.3	207	1	1
nucleotide-sugar biosynthetic process	1	0	0.3	208	1	1
peptidoglycan catabolic process	2	0	0.61	209	1	1
ribonucleotide metabolic process	3	0	0.91	210	1	1
ribonucleotide biosynthetic process	3	0	0.91	211	1	1
phospholipid catabolic process	2	0	0.61	212	1	1
folic acid and derivative biosynthetic p...	1	0	0.3	213	1	1
response to biotic stimulus	2	0	0.61	214	1	1
anatomical structure morphogenesis	5	0	1.52	215	1	1
embryonic pattern specification	1	0	0.3	216	1	1
body morphogenesis	3	0	0.91	217	1	1
monovalent inorganic cation transport	1	0	0.3	218	1	1
di-, tri-valent inorganic cation transpo...	1	0	0.3	219	1	1
inorganic anion transport	1	0	0.3	220	1	1
lipid catabolic process	2	0	0.61	221	1	1
carbohydrate biosynthetic process	1	0	0.3	222	1	1
organic acid biosynthetic process	1	0	0.3	223	1	1
phosphorylation	1	0	0.3	224	1	1
protein processing	1	0	0.3	225	1	1
intein-mediated protein splicing	1	0	0.3	226	1	1

Table 8 (contd)

Term	Annotated	Significant	Expected	Rank in classic Fisher	classic Fisher	elim Fisher
cell wall catabolic process	2	0	0.61	227	1	1
molting cycle, protein-based cuticle	1	0	0.3	228	1	1
molting cycle, collagen and cuticulin-ba...	1	0	0.3	229	1	1
hexose metabolic process	1	0	0.3	230	1	1
hexose biosynthetic process	1	0	0.3	231	1	1
GDP-mannose metabolic process	1	0	0.3	232	1	1
cellular homeostasis	1	0	0.3	233	1	1
biological adhesion	1	0	0.3	234	1	1
metal ion transport	2	0	0.61	235	1	1
cellular cation homeostasis	1	0	0.3	236	1	1
cellular di-, tri-valent inorganic catio...	1	0	0.3	237	1	1
sphingolipid catabolic process	2	0	0.61	238	1	1
cell differentiation	1	0	0.3	239	1	1
protein splicing	1	0	0.3	240	1	1
endoplasmic reticulum unfolded protein r...	2	0	0.61	241	1	1
cell-substrate adhesion	1	0	0.3	242	1	1
cellular response to stress	2	0	0.61	243	1	1
nucleobase, nucleoside and nucleotide bi...	1	0	0.3	244	1	1
cellular response to unfolded protein	2	0	0.61	245	1	1
cellular carbohydrate biosynthetic proce...	1	0	0.3	246	1	1
nucleobase, nucleoside, nucleotide and n...	1	0	0.3	247	1	1
response to endoplasmic reticulum stress	2	0	0.61	248	1	1
multicellular organism growth	1	0	0.3	249	1	1
collagen and cuticulin-based cuticle dev...	1	0	0.3	250	1	1
regulation of locomotion	1	0	0.3	251	1	1
regulation of multicellular organism gro...	1	0	0.3	252	1	1
positive regulation of locomotion	1	0	0.3	253	1	1
positive regulation of multicellular org...	1	0	0.3	254	1	1
hermaphrodite genitalia development	2	0	0.61	255	1	1
response to chemical stimulus	2	0	0.61	256	1	1
molting cycle	1	0	0.3	257	1	1
cuticle development	1	0	0.3	258	1	1
GDP-L-fucose biosynthetic process	1	0	0.3	259	1	1
fucose biosynthetic process	1	0	0.3	260	1	1
L-fucose metabolic process	1	0	0.3	261	1	1
pigment metabolic process	1	0	0.3	262	1	1
homeostatic process	1	0	0.3	263	1	1
biopolymer modification	3	0	0.91	264	1	1
biopolymer glycosylation	1	0	0.3	265	1	1
post-translational protein modification	2	0	0.61	266	1	1
cell wall metabolic process	2	0	0.61	267	1	1
cellular lipid catabolic process	2	0	0.61	268	1	1
cellular catabolic process	2	0	0.61	269	1	1
cellular carbohydrate metabolic process	1	0	0.3	270	1	1
cell fate commitment	1	0	0.3	271	1	1
external encapsulating structure organiz...	2	0	0.61	272	1	1
IMP metabolic process	3	0	0.91	273	1	1
nucleobase biosynthetic process	1	0	0.3	274	1	1
pigment biosynthetic process	1	0	0.3	275	1	1
alcohol biosynthetic process	1	0	0.3	276	1	1
monosaccharide biosynthetic process	1	0	0.3	277	1	1
GDP-L-fucose metabolic process	1	0	0.3	278	1	1
carboxylic acid biosynthetic process	1	0	0.3	279	1	1
membrane lipid catabolic process	2	0	0.61	280	1	1
heterocycle metabolic process	2	0	0.61	281	1	1
organ development	2	0	0.61	282	1	1
embryonic morphogenesis	1	0	0.3	283	1	1
system development	2	0	0.61	284	1	1
genitalia development	2	0	0.61	285	1	1
anatomical structure development	7	0	2.13	286	1	1
cellular developmental process	1	0	0.3	287	1	1
chemical homeostasis	1	0	0.3	288	1	1
ion homeostasis	1	0	0.3	289	1	1
cofactor biosynthetic process	1	0	0.3	290	1	1
regulation of multicellular organismal p...	1	0	0.3	291	1	1
positive regulation of multicellular org...	1	0	0.3	292	1	1
cellular response to stimulus	2	0	0.61	293	1	1
response to protein stimulus	2	0	0.61	294	1	1
di-, tri-valent inorganic cation homeost...	1	0	0.3	295	1	1
iron ion homeostasis	1	0	0.3	296	1	1
cation homeostasis	1	0	0.3	297	1	1
cellular chemical homeostasis	1	0	0.3	298	1	1
nucleobase, nucleoside and nucleotide me...	3	0	0.91	299	1	1
oxidation reduction	1	0	0.3	300	1	1
regulation of biological quality	1	0	0.3	301	1	1
glycosylation	1	0	0.3	302	1	1

Table 8 (contd)
