# Supplementary material for: Genome-Wide Analysis Reveals Novel Genes Essential for Heme Homeostasis in Caenorhabditis elegans
Source: PLoS Genet. 2010 Jul 29;6(7):e1001044. doi: 10.1371/journal.pgen.1001044 (PMC2912396; doi:10.1371/journal.pgen.1001044)
Supplement: Table S9 — Gene Ontology analysis of heme-responsive genes downregulated at 500 µM heme. Each GO ID is assigned a unique function or association. Both are listed here, even if the GO ID was not used in the GO analysis figure. Green shading indicates that term was included in the corresponding GO enrichment figures. (0.09 MB PDF) [file pgen.1001044.s011.pdf]

Term	Annotated	Significant	Expected	Rank in classic Fisher	classic Fisher	elim Fisher
purine nucleotide metabolic process	3	3	0.97	1	0.031	0.031
purine nucleotide biosynthetic process	3	3	0.97	2	0.031	0.031
IMP biosynthetic process	3	3	0.97	3	0.031	0.031
nucleoside phosphate metabolic process	3	3	0.97	4	0.031	0.031
nucleotide metabolic process	3	3	0.97	5	0.031	0.031
nucleoside monophosphate metabolic proce...	3	3	0.97	6	0.031	0.031
nucleoside monophosphate biosynthetic pr...	3	3	0.97	7	0.031	0.031
purine nucleoside monophosphate metaboli...	3	3	0.97	8	0.031	0.031
purine nucleoside monophosphate biosynth...	3	3	0.97	9	0.031	0.031
purine ribonucleotide metabolic process	3	3	0.97	10	0.031	0.031
purine ribonucleotide biosynthetic proce...	3	3	0.97	11	0.031	0.031
ribonucleoside monophosphate biosyntheti...	3	3	0.97	12	0.031	0.031
ribonucleoside monophosphate metabolic p...	3	3	0.97	13	0.031	0.031
nucleotide biosynthetic process	3	3	0.97	14	0.031	0.031
purine ribonucleoside monophosphate meta...	3	3	0.97	15	0.031	0.031
purine ribonucleoside monophosphate bios...	3	3	0.97	16	0.031	0.031
ribonucleotide metabolic process	3	3	0.97	17	0.031	0.031
ribonucleotide biosynthetic process	3	3	0.97	18	0.031	0.031
IMP metabolic process	3	3	0.97	19	0.031	0.031
nucleobase, nucleoside and nucleotide me...	3	3	0.97	20	0.031	0.031
nucleobase, nucleoside, nucleotide and n...	7	5	2.25	21	0.034	0.034
carbohydrate metabolic process	6	4	1.93	22	0.083	0.083
biosynthetic process	11	6	3.54	23	0.094	0.094
cellular biosynthetic process	11	6	3.54	24	0.094	0.094
'de novo' IMP biosynthetic process	2	2	0.64	25	0.102	0.102
cellular aromatic compound metabolic pro...	2	2	0.64	26	0.102	0.102
phosphorus metabolic process	2	2	0.64	27	0.102	0.102
phosphate metabolic process	2	2	0.64	28	0.102	0.102
heterocycle metabolic process	2	2	0.64	29	0.102	0.102
reproduction	13	6	4.18	30	0.201	0.201
protein modification process	3	2	0.97	31	0.242	0.242
ion transport	3	2	0.97	32	0.242	0.242
biopolymer modification	3	2	0.97	33	0.242	0.242
growth	23	9	7.4	34	0.288	0.288
behavior	6	3	1.93	35	0.293	0.293
oviposition	6	3	1.93	36	0.293	0.293
reproductive behavior	6	3	1.93	37	0.293	0.293
multicellular organism reproduction	6	3	1.93	38	0.293	0.293
reproductive behavior in a multicellular...	6	3	1.93	39	0.293	0.293
reproductive process in a multicellular ...	6	3	1.93	40	0.293	0.293
gastrulation with mouth forming first	1	1	0.32	41	0.322	0.322
monosaccharide metabolic process	1	1	0.32	42	0.322	0.322
fucose metabolic process	1	1	0.32	43	0.322	0.322
L-fucose biosynthetic process	1	1	0.32	44	0.322	0.322
mannose metabolic process	1	1	0.32	45	0.322	0.322
cellular alcohol metabolic process	1	1	0.32	46	0.322	0.322
purine base metabolic process	1	1	0.32	47	0.322	0.322
protein amino acid phosphorylation	1	1	0.32	48	0.322	0.322
protein amino acid glycosylation	1	1	0.32	49	0.322	0.322
asparagine metabolic process	1	1	0.32	50	0.322	0.322
asparagine biosynthetic process	1	1	0.32	51	0.322	0.322
group transfer coenzyme metabolic proces...	1	1	0.32	52	0.322	0.322
folic acid and derivative metabolic proc...	1	1	0.32	53	0.322	0.322
sodium ion transport	1	1	0.32	54	0.322	0.322
phosphate transport	1	1	0.32	55	0.322	0.322
anion transport	1	1	0.32	56	0.322	0.322
cell adhesion	1	1	0.32	57	0.322	0.322
cell-matrix adhesion	1	1	0.32	58	0.322	0.322
gastrulation	1	1	0.32	59	0.322	0.322
aspartate family amino acid metabolic pr...	1	1	0.32	60	0.322	0.322
aspartate family amino acid biosynthetic...	1	1	0.32	61	0.322	0.322
glycoprotein metabolic process	1	1	0.32	62	0.322	0.322
glycoprotein biosynthetic process	1	1	0.32	63	0.322	0.322
coenzyme biosynthetic process	1	1	0.32	64	0.322	0.322
nucleobase metabolic process	1	1	0.32	65	0.322	0.322
purine base biosynthetic process	1	1	0.32	66	0.322	0.322
nucleotide-sugar metabolic process	1	1	0.32	67	0.322	0.322
nucleotide-sugar biosynthetic process	1	1	0.32	68	0.322	0.322
folic acid and derivative biosynthetic p...	1	1	0.32	69	0.322	0.322
monovalent inorganic cation transport	1	1	0.32	70	0.322	0.322
inorganic anion transport	1	1	0.32	71	0.322	0.322
carbohydrate biosynthetic process	1	1	0.32	72	0.322	0.322
phosphorylation	1	1	0.32	73	0.322	0.322
hexose metabolic process	1	1	0.32	74	0.322	0.322
hexose biosynthetic process	1	1	0.32	75	0.322	0.322

Table 9

Term	Annotated	Significant	Expected	Rank in classic Fisher	classic Fisher	elim Fisher
GDP-mannose metabolic process	1	1	0.32	76	0.322	0.322
biological adhesion	1	1	0.32	77	0.322	0.322
cell-substrate adhesion	1	1	0.32	78	0.322	0.322
nucleobase, nucleoside and nucleotide bi...	1	1	0.32	79	0.322	0.322
cellular carbohydrate biosynthetic proce...	1	1	0.32	80	0.322	0.322
nucleobase, nucleoside, nucleotide and n...	1	1	0.32	81	0.322	0.322
GDP-L-fucose biosynthetic process	1	1	0.32	82	0.322	0.322
fucose biosynthetic process	1	1	0.32	83	0.322	0.322
L-fucose metabolic process	1	1	0.32	84	0.322	0.322
pigment metabolic process	1	1	0.32	85	0.322	0.322
biopolymer glycosylation	1	1	0.32	86	0.322	0.322
cellular carbohydrate metabolic process	1	1	0.32	87	0.322	0.322
nucleobase biosynthetic process	1	1	0.32	88	0.322	0.322
pigment biosynthetic process	1	1	0.32	89	0.322	0.322
alcohol biosynthetic process	1	1	0.32	90	0.322	0.322
monosaccharide biosynthetic process	1	1	0.32	91	0.322	0.322
GDP-L-fucose metabolic process	1	1	0.32	92	0.322	0.322
embryonic morphogenesis	1	1	0.32	93	0.322	0.322
cofactor biosynthetic process	1	1	0.32	94	0.322	0.322
glycosylation	1	1	0.32	95	0.322	0.322
cellular component organization	4	2	1.29	96	0.387	0.387
reproductive process	7	3	2.25	97	0.402	0.402
regulation of growth rate	19	7	6.11	98	0.41	0.41
positive regulation of growth rate	19	7	6.11	99	0.41	0.41
cellular metabolic process	35	12	11.26	100	0.455	0.455
regulation of biological process	23	8	7.4	101	0.473	0.473
regulation of growth	20	7	6.43	102	0.478	0.478
positive regulation of growth	20	7	6.43	103	0.478	0.478
positive regulation of biological proces...	20	7	6.43	104	0.478	0.478
transport	17	6	5.47	105	0.484	0.484
localization	17	6	5.47	106	0.484	0.484
establishment of localization	17	6	5.47	107	0.484	0.484
nematode larval development	5	2	1.61	108	0.519	0.519
larval development	5	2	1.61	109	0.519	0.519
macromolecule biosynthetic process	5	2	1.61	110	0.519	0.519
anatomical structure morphogenesis	5	2	1.61	111	0.519	0.519
post-embryonic development	5	2	1.61	112	0.519	0.519
cellular macromolecule biosynthetic proc...	5	2	1.61	113	0.519	0.519
biological regulation	24	8	7.72	114	0.536	0.536
peptidoglycan metabolic process	2	1	0.64	115	0.542	0.542
DNA packaging	2	1	0.64	116	0.542	0.542
establishment or maintenance of chromati...	2	1	0.64	117	0.542	0.542
chromatin assembly or disassembly	2	1	0.64	118	0.542	0.542
nucleosome assembly	2	1	0.64	119	0.542	0.542
cellular amino acid and derivative metab...	2	1	0.64	120	0.542	0.542
amino acid metabolic process	2	1	0.64	121	0.542	0.542
coenzyme metabolic process	2	1	0.64	122	0.542	0.542
nitrogen compound metabolic process	2	1	0.64	123	0.542	0.542
cation transport	2	1	0.64	124	0.542	0.542
organelle organization	2	1	0.64	125	0.542	0.542
cell wall organization	2	1	0.64	126	0.542	0.542
amino acid biosynthetic process	2	1	0.64	127	0.542	0.542
peptidoglycan catabolic process	2	1	0.64	128	0.542	0.542
cellular amine metabolic process	2	1	0.64	129	0.542	0.542
amine biosynthetic process	2	1	0.64	130	0.542	0.542
cell wall catabolic process	2	1	0.64	131	0.542	0.542
cellular component assembly	2	1	0.64	132	0.542	0.542
metal ion transport	2	1	0.64	133	0.542	0.542
chromatin assembly	2	1	0.64	134	0.542	0.542
cellular macromolecular complex subunit ...	2	1	0.64	135	0.542	0.542
cellular macromolecular complex assembly	2	1	0.64	136	0.542	0.542
cellular nitrogen compound metabolic pro...	2	1	0.64	137	0.542	0.542
nucleosome organization	2	1	0.64	138	0.542	0.542
post-translational protein modification	2	1	0.64	139	0.542	0.542
macromolecular complex subunit organizat...	2	1	0.64	140	0.542	0.542
cell wall metabolic process	2	1	0.64	141	0.542	0.542
nitrogen compound biosynthetic process	2	1	0.64	142	0.542	0.542
external encapsulating structure organiz...	2	1	0.64	143	0.542	0.542
cofactor metabolic process	2	1	0.64	144	0.542	0.542
chromosome organization	2	1	0.64	145	0.542	0.542
macromolecular complex assembly	2	1	0.64	146	0.542	0.542
protein-DNA complex assembly	2	1	0.64	147	0.542	0.542
cellular process	46	15	14.8	148	0.547	0.547
organic acid metabolic process	6	2	1.93	149	0.631	0.631
lipid transport	6	2	1.93	150	0.631	0.631
carboxylic acid metabolic process	6	2	1.93	151	0.631	0.631

Table 9 (contd)

Term	Annotated	Significant	Expected	Rank in classic Fisher	classic Fisher	elim Fisher
multicellular organismal process	39	12	12.55	152	0.668	0.668
response to stimulus	10	3	3.22	153	0.683	0.683
transcription	3	1	0.97	154	0.692	0.692
transcription, DNA-dependent	3	1	0.97	155	0.692	0.692
regulation of transcription, DNA-depende...	3	1	0.97	156	0.692	0.692
regulation of biosynthetic process	3	1	0.97	157	0.692	0.692
body morphogenesis	3	1	0.97	158	0.692	0.692
regulation of gene expression	3	1	0.97	159	0.692	0.692
regulation of macromolecule biosynthetic...	3	1	0.97	160	0.692	0.692
carbohydrate catabolic process	3	1	0.97	161	0.692	0.692
RNA metabolic process	3	1	0.97	162	0.692	0.692
regulation of nucleobase, nucleoside, nu...	3	1	0.97	163	0.692	0.692
regulation of metabolic process	3	1	0.97	164	0.692	0.692
regulation of cellular metabolic process	3	1	0.97	165	0.692	0.692
regulation of cellular biosynthetic proc...	3	1	0.97	166	0.692	0.692
RNA biosynthetic process	3	1	0.97	167	0.692	0.692
regulation of transcription	3	1	0.97	168	0.692	0.692
regulation of RNA metabolic process	3	1	0.97	169	0.692	0.692
regulation of macromolecule metabolic pr...	3	1	0.97	170	0.692	0.692
anatomical structure development	7	2	2.25	171	0.723	0.723
embryonic development	18	5	5.79	172	0.757	0.757
primary metabolic process	41	12	13.19	173	0.758	0.758
multicellular organismal development	35	10	11.26	174	0.776	0.776
gene expression	4	1	1.29	175	0.794	0.794
monocarboxylic acid metabolic process	4	1	1.29	176	0.794	0.794
cellular biopolymer biosynthetic process	4	1	1.29	177	0.794	0.794
biopolymer biosynthetic process	4	1	1.29	178	0.794	0.794
developmental process	37	10	11.9	179	0.848	0.848
catabolic process	5	1	1.61	180	0.862	0.862
regulation of cellular process	5	1	1.61	181	0.862	0.862
embryonic development ending in birth or...	17	4	5.47	182	0.867	0.867
cellular biopolymer metabolic process	22	5	7.08	183	0.908	0.908
cellular macromolecule metabolic process	22	5	7.08	184	0.908	0.908
metabolic process	56	15	18.02	185	0.92	0.92
aging	11	2	3.54	186	0.924	0.924
determination of adult life span	11	2	3.54	187	0.924	0.924
multicellular organismal aging	11	2	3.54	188	0.924	0.924
locomotion	11	2	3.54	189	0.924	0.924
protein metabolic process	19	4	6.11	190	0.924	0.924
cellular protein metabolic process	19	4	6.11	191	0.924	0.924
macromolecule metabolic process	23	5	7.4	192	0.93	0.93
biopolymer metabolic process	23	5	7.4	193	0.93	0.93
proteolysis	15	2	4.83	194	0.982	0.982
biological_process	115	37	37	195	1	1
transition metal ion transport	1	0	0.32	196	1	1
M phase	1	0	0.32	197	1	1
cell fate specification	1	0	0.32	198	1	1
morphogenesis of an epithelium	1	0	0.32	199	1	1
reproductive developmental process	2	0	0.64	200	1	1
polysaccharide metabolic process	1	0	0.32	201	1	1
chitin metabolic process	1	0	0.32	202	1	1
chitin catabolic process	1	0	0.32	203	1	1
amino sugar metabolic process	1	0	0.32	204	1	1
glucosamine metabolic process	1	0	0.32	205	1	1
glucosamine catabolic process	1	0	0.32	206	1	1
N-acetylglucosamine metabolic process	1	0	0.32	207	1	1
N-acetylglucosamine catabolic process	1	0	0.32	208	1	1
translation	1	0	0.32	209	1	1
protein folding	1	0	0.32	210	1	1
glutamine metabolic process	1	0	0.32	211	1	1
glutamine biosynthetic process	1	0	0.32	212	1	1
lipid metabolic process	9	0	2.9	213	1	1
fatty acid metabolic process	3	0	0.97	214	1	1
fatty acid biosynthetic process	1	0	0.32	215	1	1
acyl-CoA metabolic process	1	0	0.32	216	1	1
membrane lipid metabolic process	2	0	0.64	217	1	1
phospholipid metabolic process	2	0	0.64	218	1	1
sphingolipid metabolic process	2	0	0.64	219	1	1
sphingomyelin metabolic process	2	0	0.64	220	1	1
sphingomyelin catabolic process	2	0	0.64	221	1	1
iron ion transport	1	0	0.32	222	1	1
cellular ion homeostasis	1	0	0.32	223	1	1
cellular iron ion homeostasis	1	0	0.32	224	1	1
response to stress	4	0	1.29	225	1	1
defense response	2	0	0.64	226	1	1
ER-nuclear signaling pathway	2	0	0.64	227	1	1

Table 9 (contd)

Term	Annotated	Significant	Expected	Rank in classic Fisher	classic Fisher	elim Fisher
response to unfolded protein	2	0	0.64	228	1	1
cell cycle	1	0	0.32	229	1	1
chromosome segregation	1	0	0.32	230	1	1
meiosis	1	0	0.32	231	1	1
cell communication	3	0	0.97	232	1	1
signal transduction	2	0	0.64	233	1	1
intracellular signaling cascade	2	0	0.64	234	1	1
pattern specification process	1	0	0.32	235	1	1
sex differentiation	2	0	0.64	236	1	1
protein-based cuticle development	1	0	0.32	237	1	1
lipid biosynthetic process	1	0	0.32	238	1	1
glutamine family amino acid metabolic pr...	1	0	0.32	239	1	1
glutamine family amino acid biosynthetic...	1	0	0.32	240	1	1
phospholipid catabolic process	2	0	0.64	241	1	1
response to biotic stimulus	2	0	0.64	242	1	1
embryonic pattern specification	1	0	0.32	243	1	1
gas transport	1	0	0.32	244	1	1
oxygen transport	1	0	0.32	245	1	1
di-, tri-valent inorganic cation transpo...	1	0	0.32	246	1	1
lipid catabolic process	2	0	0.64	247	1	1
organic acid biosynthetic process	1	0	0.32	248	1	1
protein processing	1	0	0.32	249	1	1
intein-mediated protein splicing	1	0	0.32	250	1	1
molting cycle, protein-based cuticle	1	0	0.32	251	1	1
molting cycle, collagen and cuticulin-ba...	1	0	0.32	252	1	1
cellular homeostasis	1	0	0.32	253	1	1
cell cycle process	1	0	0.32	254	1	1
cell cycle phase	1	0	0.32	255	1	1
cellular cation homeostasis	1	0	0.32	256	1	1
cellular di-, tri-valent inorganic catio...	1	0	0.32	257	1	1
sphingolipid catabolic process	2	0	0.64	258	1	1
cell differentiation	1	0	0.32	259	1	1
protein splicing	1	0	0.32	260	1	1
endoplasmic reticulum unfolded protein r...	2	0	0.64	261	1	1
cellular response to stress	2	0	0.64	262	1	1
cellular response to unfolded protein	2	0	0.64	263	1	1
response to endoplasmic reticulum stress	2	0	0.64	264	1	1
multicellular organism growth	1	0	0.32	265	1	1
collagen and cuticulin-based cuticle dev...	1	0	0.32	266	1	1
regulation of locomotion	1	0	0.32	267	1	1
regulation of multicellular organism gro...	1	0	0.32	268	1	1
positive regulation of locomotion	1	0	0.32	269	1	1
positive regulation of multicellular org...	1	0	0.32	270	1	1
hermaphrodite genitalia development	2	0	0.64	271	1	1
response to chemical stimulus	2	0	0.64	272	1	1
molting cycle	1	0	0.32	273	1	1
cuticle development	1	0	0.32	274	1	1
homeostatic process	1	0	0.32	275	1	1
cellular lipid catabolic process	2	0	0.64	276	1	1
cellular catabolic process	2	0	0.64	277	1	1
cellular lipid metabolic process	5	0	1.61	278	1	1
meiotic chromosome segregation	1	0	0.32	279	1	1
cell fate commitment	1	0	0.32	280	1	1
amino sugar catabolic process	1	0	0.32	281	1	1
carboxylic acid biosynthetic process	1	0	0.32	282	1	1
membrane lipid catabolic process	2	0	0.64	283	1	1
organ development	2	0	0.64	284	1	1
system development	2	0	0.64	285	1	1
genitalia development	2	0	0.64	286	1	1
cellular developmental process	1	0	0.32	287	1	1
chemical homeostasis	1	0	0.32	288	1	1
ion homeostasis	1	0	0.32	289	1	1
regulation of multicellular organismal p...	1	0	0.32	290	1	1
positive regulation of multicellular org...	1	0	0.32	291	1	1
meiotic cell cycle	1	0	0.32	292	1	1
M phase of meiotic cell cycle	1	0	0.32	293	1	1
cellular response to stimulus	2	0	0.64	294	1	1
response to protein stimulus	2	0	0.64	295	1	1
di-, tri-valent inorganic cation homeost...	1	0	0.32	296	1	1
iron ion homeostasis	1	0	0.32	297	1	1
cation homeostasis	1	0	0.32	298	1	1
cellular chemical homeostasis	1	0	0.32	299	1	1
transmembrane transport	1	0	0.32	300	1	1
oxidation reduction	1	0	0.32	301	1	1
regulation of biological quality	1	0	0.32	302	1	1

Table 9 (contd)
